# Species diversity driven by morphological and ecological disparity: a case study of comparative seed morphology and anatomy across a large monocot order

**DOI:** 10.1093/aobpla/plw063

**Published:** 2016-10-26

**Authors:** John C. Benedict, Selena Y. Smith, Chelsea D. Specht, Margaret E. Collinson, Jana Leong-Škorničková, Dilworth Y. Parkinson, Federica Marone

**Affiliations:** 1 Department of Earth and Environmental Sciences, University of Michigan, Ann Arbor, MI 48109-1005, USA; 2 Museum of Paleontology, University of Michigan, Ann Arbor, MI 48109-1079, USA; 3 Department of Plant and Microbial Biology, Integrative Biology and the University and Jepson Herbaria, University of California, Berkeley, CA 94750-2465, USA; 4 Department of Earth Sciences, Royal Holloway University of London, London TW20 0EX, UK; 5 Herbarium, Singapore Botanic Gardens, National Parks Board, 259569 Singapore; 6 Advanced Light Source, Lawrence Berkeley National Labs, Berkeley, CA 94720, USA; 7 Swiss Light Source, Paul Scherrer Institut, 5232 Villigen PSI, Switzerland

**Keywords:** Cannaceae, Costaceae, Heliconiaceae, Lowiaceae, Marantaceae, Musaceae, Strelitziaceae, Zingiberaceae

## Abstract

Phenotypic variation can be attributed to genetic heritability as well as biotic and abiotic factors. Across Zingiberales, there is a high variation in the number of species per clade and in phenotypic diversity. Factors contributing to this phenotypic variation have never been studied in a phylogenetic or ecological context. Seeds of 166 species from all eight families in Zingiberales were analyzed for 51 characters using synchrotron based 3D X-ray tomographic microscopy to determine phylogenetically informative characters and to understand the distribution of morphological disparity within the order. All families are distinguishable based on seed characters. Non-metric multidimensional scaling analyses show Zingiberaceae occupy the largest seed morphospace relative to the other families, and environmental analyses demonstrate that Zingiberaceae inhabit both temperate and tropical regions, while other Zingiberales are almost exclusively tropical. Temperate species do not cluster in morphospace nor do they share a common suite of character states. This suggests that the diversity seen is not driven by adaptation to temperate niches; rather, the morphological disparity seen likely reflects an underlying genetic plasticity that allowed Zingiberaceae to repeatedly colonize temperate environments. The notable morphoanatomical variety in Zingiberaceae seeds may account for their extraordinary ecological success and high species diversity as compared to other Zingiberales.

## Abstract (precis)

The banana and ginger group, order Zingiberales, comprises of exceptionally diverse, primarily tropical, plants. Within Zingiberales, the ginger family (Zingiberaceae) is unique in being not only the most species-rich but also the most disparate in terms of their morphology and anatomy, and the only group with a substantial number of species in temperate environments. Multiple radiations into temperate habitats were found to not be driven by morphological change, but instead may point to a genetic plasticity in the family that provided opportunities for speciation events not found anywhere else in the order.

## Introduction

Understanding what processes account for the diversity of life on Earth is a fundamental question in biology. There are a myriad of factors and influences that contribute to the genotypic and phenotypic diversity of a taxon, including the complex evolutionary histories within and between species, the array of ecological space that a taxon inhabits and the overall developmental and genetic variation that provide the raw material for the evolution of new forms and functions (Cowling *et al.*, 1996; Baldwin and Sanderson, 1998; Barrier *et al.*, 1999). Documenting the morphological and anatomical diversity of organisms through time, incorporating data from both extant organisms and their extinct ancestors preserved in the fossil record, is fundamental to understanding diversity. By drawing correlations between current mechanisms of selection and those that may have been acting in the past, such studies can begin to address the tempo and mode of phenotypic changes that have occurred from deep time through to the present. This includes how past organisms may have responded to environmental variables or have developed ecological tolerances.

Within angiosperms, the Zingiberales (bananas, gingers and relatives) are a large monophyletic order of monocotyledonous plants that serve as a model group for understanding the mechanisms underlying diversity through time (Kress and Specht, 2005). Based on molecular sequence data, the Zingiberales underwent a proposed rapid radiation in the Cretaceous (Kress and Specht, 2006; Sass *et al.*, 2016), resulting in the eight families of the order. Out of ca. 2500 extant species in the order, the number of species varies substantially from seven in Strelitziaceae to ca. 1600 in Zingiberaceae (The Plant List, 2013). Likewise, the phenotypic diversity of the eight families varies widely with respect to floral, vegetative and anatomical characters as well as diversity of life history strategies and environmental/ecological ranges (Kress and Specht, 2005, 2006).

**Figure 1:**
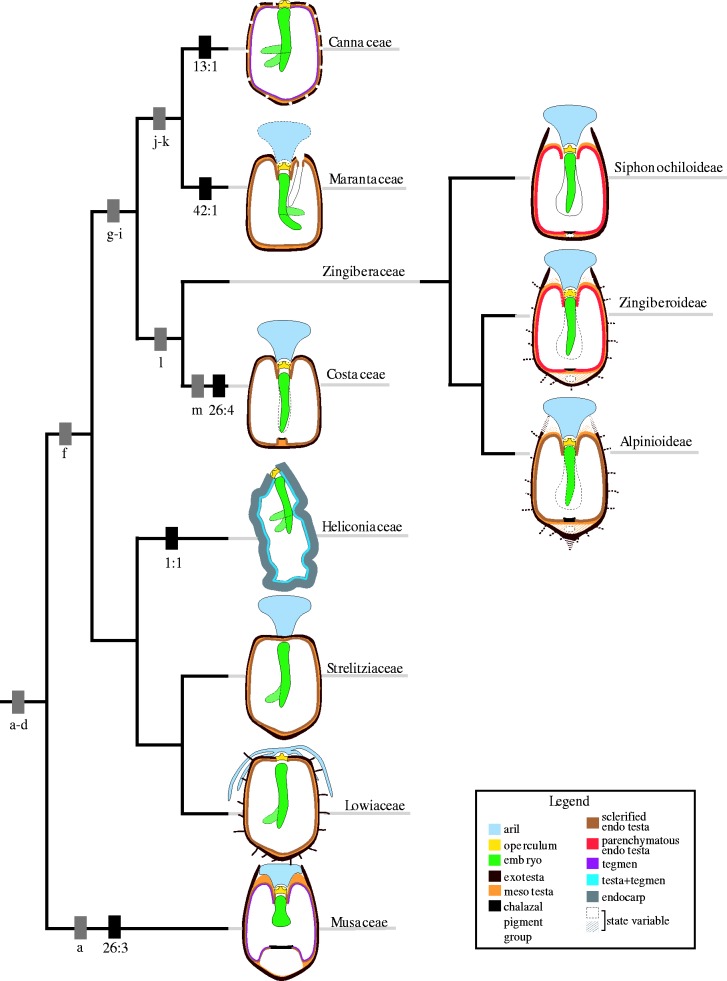
Zingiberales phylogeny based on Sass *et al.* (2016) illustrating apomorphic characters and generalized seed diagrams. Seed characters are denoted by black rectangles with character: character state underneath (defined in Table 1). Non-seed characters are denoted by grey rectangles with letters beneath: (a) leaves penni-parallel with air chambers, (b) supervolute ptyxis (one half of the margin of a developing leaf curled inside the other half of the margin), (c) silica bodies, (d) ovary inferior, (e) spirally arranged leaves, (f) leaves distichous or monostichous, (g) raphides absent, (h) one fertile stamen, (i) staminodes petaloid, (j) flowers asymmetric, (k) anther bisporangiate, (l) sepals connate, (m) leaves monostichous [amended from Simpson (2010)].

The Zingiberales are found primarily in the tropics and subtropics worldwide (Kress *et al.*, 2001) and form a well-supported clade based on molecular and morphological characters. The order has been informally divided into two groups, the monophyletic ‘ginger group’ (Zingiberaceae, Costaceae, Marantaceae and Cannaceae) which is supported by several apomorphies, and the paraphyletic ‘banana group’ (Musaceae, Strelitziaceae, Lowiaceae and Heliconiaceae; Kress and Specht, 2005; Simpson, 2010; Sass *et al.*, 2016; Fig. 1). Previous studies have addressed the genetic basis for floral diversity in the group (Specht and Bartlett, 2009; Bartlett and Specht, 2010, 2011; Specht *et al.*, 2012). In addition, the family Zingiberaceae has been shown to possess very morphologically diverse seed and embryo structures (Benedict *et al.*, 2015a, b), but less is known about seed diversity in the other families of the order. An understanding of seed structural diversity will contribute to our ability to untangle the complex evolutionary history of this economically and ecologically important group of plants by allowing inclusion and re-evaluation of fossils, and, more broadly, to explore what factors independently influence the diversity of different lineages.

While many anatomical and developmental studies on Zingiberales seeds exist (e.g. Cannaceae: Grootjen and Bouman, 1988; Costaceae: Grootjen and Bouman, 1981; Heliconiaceae: Simão *et al.*, 2006; Lowiaceae: Wen *et al.*, 1997; Marantaceae: Grootjen, 1983; Musaceae: McGahan, 1961; Bouharmont, 1963; Strelitziaceae: Takhtajan, 1985; Zingiberaceae: Sachar and Arora, 1963; Liao and Wu, 1996, 2000), few have undertaken detailed comparisons between the families. Such comparative analyses are necessary to fully characterize the diversity of seed structures within the group, to determine which characters are plesiomorphic and which are derived by placing these structures in a phylogenetic context, and to test the placement of fossils within the appropriate lineages through time. For the few studies in which comparisons were made across families (Grootjen and Bouman, 1981; Rodríguez-de la Rosa and Cevallos-Ferriz, 1994; Liao *et al.*, 2004; Tang *et al.*, 2005; Fischer *et al.*, 2009; Panja and Maiti, 2012), few characters were considered, and these studies did not evaluate homology of characters or test for homoplasy among characters and character states. For example, while seed characters were demonstrated to be useful for distinguishing among the various tribes and subfamilies of Zingiberaceae (Benedict *et al.*, 2015a, b), high levels of homoplasy in these characters meant that the evolutionary history of the clades could not be recovered by seed characters alone (Benedict *et al.*, 2015b). Adding to the difficulty of an ordinal-wide evaluation are studies that include incorrect interpretation of tissues in an ontogenetic context, or use descriptive rather than developmental terminology [e.g. *Heliconia* diaspores described as ‘seeds’ (e.g. Rodríguez-de la Rosa and Cevallos-Ferriz, 1994; Panja and Maiti, 2012); Cannaceae exotesta described as Malpighian cells (Graven *et al.*, 1997)].

This paper aims to: (1) resolve inconsistencies in various interpretations of structures previously reported in the literature, (2) determine if seed characters can be used to differentiate the eight families of Zingiberales and (3) explore how species diversity may be driven by morphological and ecological disparity and what processes may account for variation in diversity between families of Zingiberales.

## Methods

One hundred and sixty-six taxa from all eight families of Zingiberales [**Supporting Information**—Table S1] were sampled from various herbaria, botanical gardens or commercial growers and analyzed for 51 internal and external seed characters [Table 1 and **Supporting Information**—Table S2, and Notes S1 for detailed description of characters]. The number of seeds studied per taxon ranged from one to more than 50 [**Supporting Information**—Table S1]. Specimens scanned using SRXTM are deposited at the University of Michigan Herbarium (MICH) excluding specimens on non-destructive loans (*Alpinia purpurata*, *Siphonochilus aethiopicus* and *S. kirkii*), which will be returned to the institutions from which they were loaned.

**Table 1.** List of seed characters and character states used in this study and their homoplasy indices.

Characters	Character states	Homoplasy index (HI)
1. Sclerenchymatous endocarp	0: absent; 1: present	0.00
2. Natural seed/endocarp color	0: white/ cream/ grey; 1: tan/ red/ light brown; 2: dark brown/ black	0.93
3. Seed surface	0: smooth; 1: striate; 2: verrucose; 3: rugose; 4: ruminate	0.62
4. Trichomes on seed coat or aril	0: absent; 1: present	0.86
5. Aril	0: absent; 1: present only at micropylar end of seed; 2: present covering part or most of seed, lacinate or not, easily separated from dry seeds; 3. Present covering entire seed, often slimy or juicy when fresh and as a thin layer on dried material.	0.77
6. Seed shape	0: ellipsoid; 1: ovoid; 2: oblate; 3: polyhedral; 4: irregular	0.92
7. Seeds contorted from arrangement in fruit	0: absent; 1: present	0.96
8. Seed length	0: less than twice as long as wide; 1: twice as long as wide	0.96
9. Seed body taper at micropyle	0: absent; 1: present	0.97
10. Seed body taper at chalaza	0: absent; 1: present	0.97
11. Externally visible raphe	0: absent; 1: present, single; 2: present, double	0.87
12. External chalazal indentation	0: absent; 1: present	0.92
13. Stomata in seed coat	0: absent; 1: present	0.00
14. Micropylar region shape	0: absent/ not clearly defined; 1: present, conical; 2: cylindrical	0.89
15. Operculum	0: absent; 1: present	0.75
16. Operculum layering	0: absent; 1: homogenous; 2: multilayered	0.95
17. Operculum position	0: absent; 1: apical; 2: subapical	0.67
18. Micropylar collar	0: absent; 1: present	0.80
19. Micropylar collar layering	0: absent; 1: formed from endotesta; 2: formed from endotesta and additional layers	0.96
20. Thickened micropylar collar	0: absent; 1: present	0.94
21. Recurved micropylar collar	0: absent; 1: weakly recurved; 2: strongly recurved	0.94
22. Hilar rim	0: absent; 1: present	0.91
23. Hilar rim layering	0: absent; 1: formed from exotesta; 2: formed from exotesta and mesotesta	0.83
24. Micropylar mesotestal proliferation	0: absent; 1: present	0.91
25. Massive chalazal testal proliferations	0: absent; 1: present	0.94
26. Columnar chalazal testal proliferations	0: absent; 1: present	0.00
27. Chalazal chamber	0: absent; 1: *Alpinia*-type; 2: *Amomum*-type; 3: *Musa*-type; 4: *Costus*-type	0.87
28. Chalazal chamber column	0: absent; 1: present	0.67
29. Chalazal mucro	0: absent; 1: present	0.50
30. Mechanical layer thickness	0: 1-99 µm; 1: 100-199 µm; 2: 200+ µm	0.92
31. Mechanical layer	0: outer integument; 1: inner and outer integument; 2: endocarp	0.97
32. Exotesta cell type	0: palisade; 1: isodiametric/cuboidal; 2: poorly developed or destroyed; 3: other	0.93
33. Uniform exotesta	0: absent; 1: present	0.97
34. Multiseriate exotesta	0: absent; 1: present	0.75
35. Portion of exotesta palisade at chalaza	0: absent; 1: present	0.67
36. Mesotesta layer number	0: absent; 1: 1 type; 2: 2 types; 3: 3 types	0.91
37. Endotestal shape and thickness	0: absent; 1: thin parenchyma 0-14 µm; 2: short sclerenchyma 15-29 µm; 3: elongate sclerenchyma 30+ µm	0.83
38. Endotestal gap location	0: absent; 1: base; 2: side	0.87
39. Chalazal pigment group	0: absent; 1: present, discoid; 2: present, non-discoid	0.50
40. Chalazal endotestal thickening	0: absent; 1: present	0.67
41. Inner integument	0: absent; 1: present	0.50
42. Raphe canal	0: absent; 1: present	0.96
43. Perisperm canal	0: absent; 1: present	0.00
44. Perisperm canal shape	0: simple; 1: basally branched; 2: branched throughout	0.67
45. Embryo length	0: elongate; 1: short	0.80
46. Embryo shape	0: straight; 1: L-shaped; 2: J-shaped	0.96
47. Embryo base	0: not differentiated; 1: bulbous; 2: forked	0.83
48. Embryo-seed coat contact	0: absent; 1: present	0.75
49. Well-developed perisperm	0: absent; 1: present	0.83
50. Endosperm composition	0: absent; 1: helobial; 2: nuclear	0.00
51. Basally abundant endosperm	0: absent; 1: present, weak; 2: present, strong	0.83

### Microscopy and photography

External features of the seeds were observed using a Leica MZ6 (Leica Microsystems Inc., Buffalo Grove, Illinois, USA) or Nikon SMZ1500 (Nikon Instruments Inc., New York, USA) stereomicroscope and photographed using a Macropod Pro (Macroscopic Solutions LLC, Coventry, Connecticut, USA), and images were stitched into a single image using Zerene Stacker version 1.04 software (Zerene Systems LLC, Richland, Washington, USA).

Representative seeds were embedded in Ward's Bio-plastic Synthetic Resin (Ward’s Natural Science, Rochester, New York, USA) and wafered using a Buhler Isomet low-speed lapidary saw or Buehler Isomet 1000 precision lapidary saw with a diamond blade (BUEHLER, a division of Illinois Tool Works Inc., Lake Bluff, Illinois, USA; Hass and Rowe, 1999; Benedict *et al.*, 2008; Benedict, 2015). Wafers were mounted on standard microscope slides using U-154 adhesive (The Company, Lakewood, Colorado, USA) and ground down to a minimal thickness using various grades of carborundum powder or sand paper to view anatomical features. Specimens were photographed using a Nikon D70s or D90 camera body (Nikon Inc. Melville, New York, USA) attached to a Nikon Eclipse E800 compound scope, or a Nikon Eclipse LV100ND compound scope with dedicated Nikon DS-Ri1 camera attachment.

Some seeds were fractured in transverse or longitudinal section, mounted to an aluminum stub using clear nail polish and sputter-coated with gold using a Cressington 108 Auto Sputter Coater (Cressington Scientific Instruments Ltd, Watford, England). Samples were sputter-coated for 60 s at 30 mA, and examined with a JEOL JSM-5510 Scanning Electron Microscope (JEOL USA, Inc., Peabody, Massachusetts, USA) at 5 kV. Both wafering and scanning electron microscopy were done on representative individuals to compare anatomical features previously described based on these methods to the data obtained using synchrotron based X-ray tomography.

### Synchrotron based X-ray tomographic microscopy

Samples were mounted onto brass stubs or toothpicks using a PVA glue or epoxy and imaged using standard absorption contrast at the TOMCAT beamline at the Swiss Light Source (SLS; Stampanoni *et al.*, 2006; Paul Scherrer Institut, Villigen, Switzerland); the 2-BM beamline at the Advanced Photon Source (APS; Argonne National Laboratory, Lemont, Illinois, USA); or the 8.3.2 beamline at the Advanced Light Source (ALS; MacDowell *et al.*, 2012; Lawrence Berkeley National Laboratory, Berkeley, California).

At TOMCAT, specimens were scanned in 2009, 2010, 2011, 2013 and 2015. Projection data were magnified by 2×, 4× or 20× microscope objectives and digitized by a high-resolution CCD camera (pco.2000; PCO GmbH, Kelheim, Germany; 2009–2011) or sCMOS camera (pco.edge 5.5; PCO GmbH, Kelheim, Germany; 2013 and 2015). Samples were scanned using 10 or 13 keV and an exposure time per projection of 50, 125, 150 or 200 milliseconds. For each scan, a total of 1501 projections were acquired over 180°. Reconstruction of the tomographic data was performed on a multi-node Linux PC cluster using a highly optimized routine based on the Fourier transform method and a gridding procedure (Marone *et al.* 2010; Marone and Stampanoni 2012), resulting in a theoretical pixel size of 3.7 µm at 2× and 1.85 µm at 4× (2009–2011) or 3.25 µm at 2× and 1.625 µm at 4× (2013–2015) for reconstructed images.

At 2-BM, specimens were scanned during sessions in 2011 and 2012. 2.5×, 4× or 5× microscope objectives were used to magnify the projection data, and a Coolsnap K4 camera (Photometrics, Tucson, Arizona, 2011 and February 2012) or pco.dimax high-speed camera (PCO GmbH, Kelheim, Germany, June 2012) was used to digitize the data. Samples were scanned at 16.1 or 21 keV with an exposure time of 280–700 ms. For each scan, a total of 1500 projections were acquired over 180°. The tomographic reconstructions were conducted with a 64-node cluster at APS using a gridrec reconstruction algorithm (Dowd *et al.*, 1999). Reconstructed images taken with the Coolsnap K4 had a theoretical pixel size of 3.7 µm at 2×, 2.96 µm at 2.5×, 1.85 µm at 4× and 1.48 µm at 5×, and those taken with the pco.dimax had a theoretical pixel size of 5.5 µm at 2×, 4.4 µm at 2.5×, 2.75 µm at 4x× and 2.2 µm at 5×.

At the 8.3.2 beamline, specimens were scanned during sessions in 2013, 2014 and 2015. Samples were magnified with either a 2× or 5× microscope objective and digitized using a sCMOS camera (pco.edge; PCO GmbH, Kelheim, Germany). Samples were scanned at 15 keV with an exposure time of 90, 500 or 950 ms. For each scan, a total of 2049 projections were acquired over 180°. Reconstruction was carried out using a custom ImageJ (Rasband 1997–2016) plugin for image preprocessing and Octopus (Inside Matters, Aalst, Belgium) for tomographic reconstruction. Reconstructed images had a theoretical pixel size of 3.25 µm at 2× and 1.3 µm at 5×.

Reconstructed images were processed at the University of Michigan using Avizo 7.0 or 8.0 (FEI Visualization Science Group, Burlington, Massachusetts, USA) for Windows 7. Images were captured in Avizo 7.0 or 8.0 and edited uniformly for contrast using Adobe Photoshop CS2 or CS6 (Adobe Systems Incorporated, San Jose, California, USA).

### Character analysis

The character matrix [**Supporting Information**—Table S2] was imported into Mesquite v.3.03 (Maddison and Maddison, 2015) and characters were traced using parsimony onto topologies based on the topology recovered by Sass *et al.* (2016). To extrapolate consistency, homoplasy and retention indices, intrafamiliar relationships were established based on the following studies: Cannaceae (Prince, 2010), Costaceae (Specht, 2006), Marantaceae (Prince and Kress, 2006), Musaceae (Liu *et al.*, 2010), Strelitziaceae (Cron *et al.*, 2012), Zingiberaceae (Wood *et al.*, 2000; Kress *et al.*, 2002, 2007; Leong-Škorničková et al., 2011).

### Non-metric multidimensional scaling (NMDS) analysis

The character matrix also was imported into the program Past 3.08 (Hammer *et al.*, 2001) and used for a NMDS analysis. NMDS analysis in Past cannot be executed with polymorphic character states, so these were added as additional character states to the matrix [e.g. if a taxon had 1and3 for character X with three character states, then a fourth character was added into the matrix representing 1and3]. Three similarity indices were tested (Euclidean, Gower and Simpson), and Gower’s was used because it resulted in the lowest stress index (Euclidean = 0.2993, Gower = 0.2769, Simpson = 0.4322).

### Ecological/environmental data mapping

Occurrence data for all taxa in Zingiberales were downloaded from GBIF (www.gbif.org). Duplicated occurrences, occurrences with georeferences outside natural distributions, in oceans, or in urban areas were removed. A subset of occurrences was also created, with seed data described in the current study. Data were imported into ArcGIS 10.1 and joined with WorldClim Global Climate Data at 30 s resolution (www.worldclim.org, Hijmans *et al.*, 2005) and Terrestrial Ecoregions of the Worlds (TEOW) biome data (Olson *et al.*, 2001) to extrapolate biome and environmental data correlations with taxon occurrences [Table 2, **Supporting Information**—Tables S3 and S4].

**Table 2.** Environmental data based on GBIF occurrence data for extant taxa in Zingiberales and the subset of Zingiberales in this current study (accessed 31 August 2015), combined with WorldClim Global Climate variables (Hijmans *et al.*, 2005) and Terrestrial Ecoregions of the World (TEOW) biome categories (Olson *et al.*, 2001).

	Occurrences	Tropical and subtropical (%)	Temperate (%)	Other (%)	Minimum altitude (m)	Maximum altitude (m)	Average annual precipitation (mm)	Minimum coldest month temperature(°C)	Average mean annual temperature(°C)
*All data included*									
Cannaceae	1490	92	1	7	−3	4596	1747	−8.9	22
Costaceae	7534	98	0	2	−1	4739	2535	−6.1	24.2
Heliconiaceae	9652	98	0	2	−1	4849	2540	−12.4	23.8
Lowiaceae	18	100	0	0	13	1463	2456	9.8	24.8
Marantaceae	20256	97	0	3	−17	4600	2391	−10	24.4
Musaceae	363	94	4	2	5	2483	2343	−2.4	22.7
Strelitziaceae	156	89	0	11	5	1661	1940	1.4	23.5
Zingiberaceae	15840	86	12	2	−17	4624	2305	−14.7	22.1
*Subset only*									
Cannaceae	935	95	0	5	−3	4596	1834	−8.9	22.2
Costaceae	2234	97	0	3	0	4739	2466	−6.1	24.3
Heliconiaceae	2134	98	0	2	−1	4596	2569	−8.2	24.2
Lowiaceae	3	100	0	0	278	291	2363	21.4	25.6
Marantaceae	3274	96	0	4	−17	4516	2202	−10	24.8
Musaceae	87	94	1	5	7	2370	2001	2.1	22.2
Strelitziaceae	130	94	0	6	5	1661	2107	1.4	24.4
Zingiberaceae	3806	64	34	2	−17	4624	2094	−12.4	20.5

The biome category “other” is a combination of desert and xeric shrublands, flooded grasslands and savannas, mangroves, mediterranean forests, woodlands and scrubs, as well as montane grasslands and shrublands.

## Results

### Zingiberales seed structure in a systematic context

Previously described and newly discovered synapomorphies were mapped onto the most recent phylogeny based on molecular data (Sass *et al.*, 2016; Fig. 1). Results are summarized in Table 3, and complete results for all species studied are in **Supporting Information**—Table S2. Character numbers (Table 1) are given in parentheses in the following text.

**Table 3.** Significant seed characters and character states shared by each family in Zingiberales.

Families	Number of shared characters	Autapomorphic seed characters: character state	Distinguishing suite of characters: character state
Cannaceae	36	13:1	2:2, 31:1, 32:0, 42:0
Costaceae	29	27:4	21:1, 35:1, 45:0, 46:0, 47:0
Heliconiaceae	35	1:1	3:4, 15:1, 16:1, 17:1
Lowiaceae	30	None	4:1, 5:4, 18:0, 27:0, 30:0
Marantaceae	31	43:1	12:0, 15:1, 18:1, 20:0, 21:1, 22:0, 27:0, 39:0
Musaceae	27	27:3	14:2, 31:1, 42:1, 45:1, 47:1
Strelitziaceae	29	None	2:2, 4:0, 15:0, 18:0, 27:0, 40:1
Zingiberaceae	9	None	1:0, 13:0, 31:0, 35:0, 43:0

**Figure 2:**
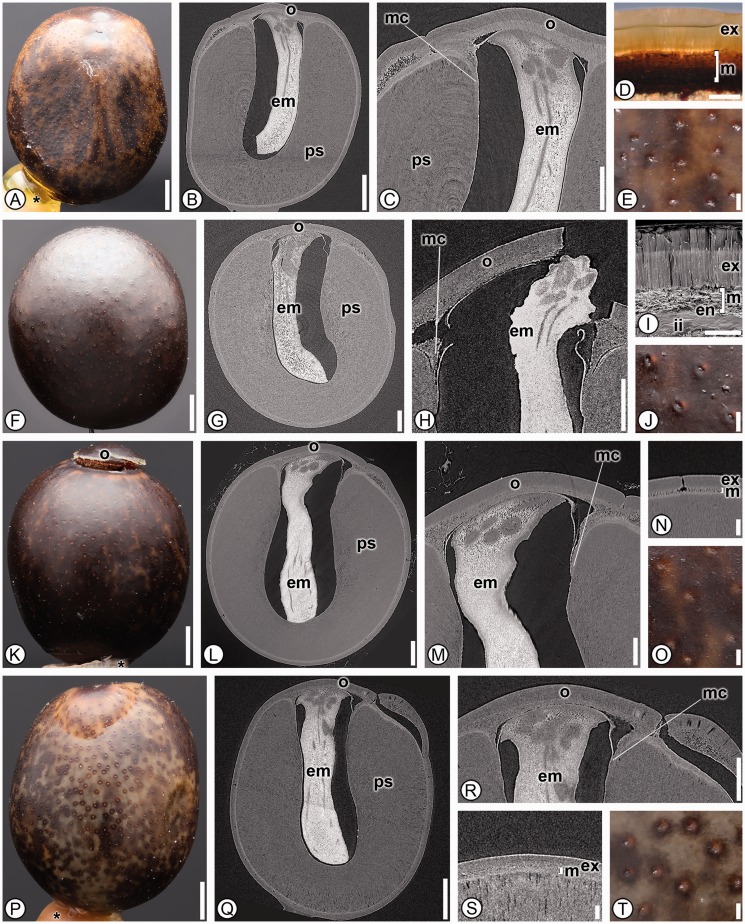
Seed anatomy in Cannaceae. (A, D–F, J–K, O–P, T): light micrographs; (I): scanning electron micrograph; (B–C, G–H, L–M, Q–R): digital longitudinal sections; (N, S): digital transverse sections. (A–E) *Canna paniculata*. (A) Overview of dark brown seed. (B) Internal morphology with operculum (o), elongate J-shaped embryo (em) and abundant perisperm (ps). (C) Micropylar region showing micropylar collar (mc), multilayered operculum (o), perisperm (ps) and embryo (em). (D) Seed coat with palisade exotesta (ex) and mesotesta (m) of one cell type. (E) Detail of smooth seed surface with stomata. (F–J) *Canna indica*. (F) Overview of dark brown seed. (G) Internal morphology with operculum (o), elongate J-shaped embryo (em) and abundant perisperm (ps). H. Micropylar region with dehisced operculum (o), emerging embryo (em) and micropylar collar (mc). (I) Seed coat with palisade exotesta (ex), mesotesta of one cell type (m), endotesta (en) and inner integument (ii). (J) Detail of smooth seed surface with stomata. (K–O) *Canna × generalis*. (K) Overview of dark brown seed with dehisced operculum (o). (L) Internal morphology with straight embryo (em), operculum (o) and perisperm (ps). (M) Micropylar region with micropylar collar (mc), operculum (o) and embryo (em). (N) Seed coat with palisade exotesta (ex) and mesotesta with one cell type (m). (O) Detail of smooth seed coat with stomata. (P–T) *Canna tuerckheimii*. (P) Overview of dark brown seed. (Q) Internal morphology with operculum (o), straight embryo (em) and perisperm (ps). (R) Micropylar region with multilayered operculum (o), micropylar collar (mc) and embryo (em). (S) Seed coat with palisade exotesta (ex) and mesotesta of one cell type (m). (T) Detail of smooth seed surface with stomata. *indicates mounting glue and/or specimen stub. Scale bars: A–B, F, K, P–Q = 1 mm; C, G–H, L, R = 500 µm; M = 250 µm; E, J, N–O, S–T = 100 µm; D = 50 µm.

***Cannaceae*.** Six species from the single genus *Canna* in Cannaceae were analyzed and shared 36 character states; one character, the presence of stomata on the seed coat (13), was unique to the family and thus is an autapomorphy (Fig. 2A–T). *Canna* seeds were further distinguished from other members in the order by possessing a dark brown or black colored seed (2), a mechanical layer derived from both integuments (31), a palisade exotesta (32) and in lacking a raphe canal (42; Table 3).

**Figure 3:**
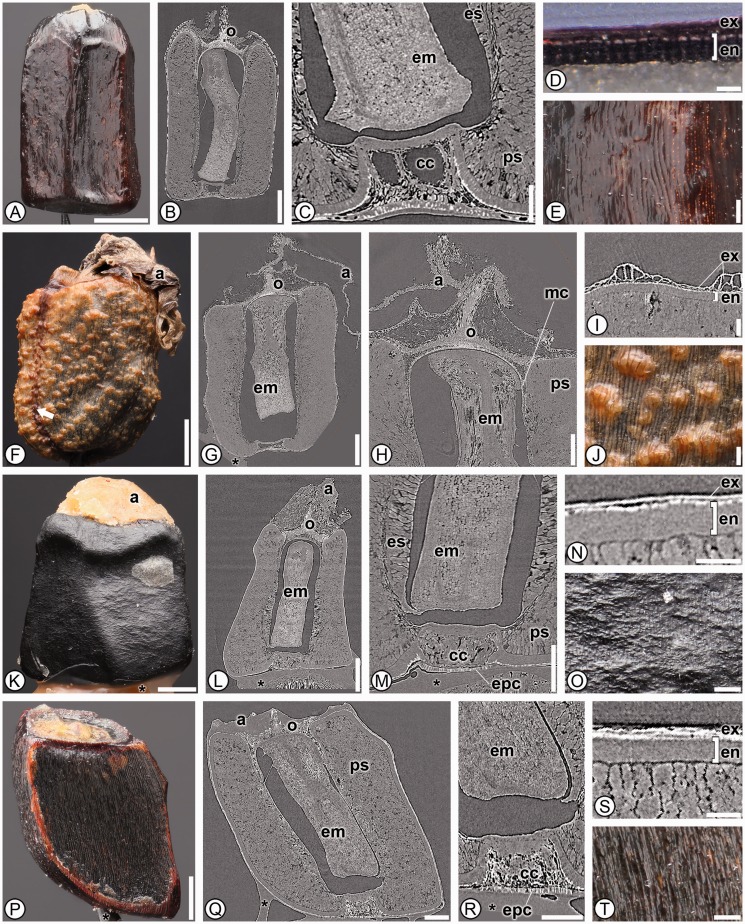
Seed anatomy in Costaceae. (A, D–F, J–K, O–P, T): light micrographs; (B–C, G–H, L–M, Q–R): SRTXM digital longitudinal sections; (I, N, S): SRTXM digital transverse sections. (A–E) *Dimerocostus argenteus*. (A) Overview of dark brown seed. (B) Internal morphology with a single layered operculum (o). (C) Chalazal region with an elongate, straight embryo (em), weak basally proliferated endosperm (es), perisperm (ps) and *Costus*-type chalazal chamber (cc). (D) Seed coat with isodiametric exotesta (ex) and sclerenchymatous endotesta (en). (E) Detail of striate seed surface. (F–J) *Monocostus uniflorus*. (F) Overview of light brown seed with solid aril (a); note externally visible raphe that is darker brown in color. (G) Internal morphology with single layered operculum (o), aril (a) and elongate straight embryo (em). (H) Micropylar region with aril (a), single layered operculum (o), micropylar collar (mc), perisperm (ps) and embryo (em). (I) Seed coat with alternating palisade and isodiametric exotesta (ex) and sclerenchymatous endotesta (en). (J) Detail of striate and verrucose seed surface. (K–O) *Costus guanaiensis*. (K) Overview of black seed with solid aril (a). (L) Internal morphology with aril (a), operculum (o) and elongate, straight embryo (em). (M) Chalazal region with embryo (em), weak basally proliferated endosperm (es), perisperm (ps), *Costus*-type chalazal chamber (cc) and portion of palisade exotesta at chalaza (epc). (N) Seed coat with poorly developed exotesta (ex) and sclerenchymatous endotesta (en). (O) Detail of striate seed surface. P–T *Tapeinochilus* sp. (P) Overview of dark brown seed. (Q) Internal morphology with aril (a), multilayered operculum (o), perisperm (ps) and elongate, straight embryo (em). (R) Chalazal region with embryo (em), *Costus*-type chalazal chamber (cc) and portion of exotesta palisade at chalaza (epc). (S) Seed coat with isodiametric exotesta (ex) and sclerenchymatous endotesta (en). (T) Detail of striate seed surface. * indicates mounting glue and/or specimen stub. Scale bars: A–B, F–G, K–M, P = 1 mm; C, H, N, Q = 500 µm; E, O, R, T = 250 µm; I–J, S =  100 µm; D = 50 µm.

***Costaceae*.** Members shared 29 character states between the 15 taxa analyzed and a single autapomorphic character state, the presence of a *Costus*-type chalazal chamber was found only in members of the family (27; a circular or square chamber in LS that is tightly surrounded by endotesta; Fig. 3A–T). They can be further distinguished from other families of the order by the combined presence of a strongly recurved micropylar collar (21), a portion of the exotesta notably palisade in the chalazal region (35) and by having an elongate, straight embryo with no differentiation at the base (45–47; Table 3). These characters in combination are not found in any other member in the order.

**Figure 4:**
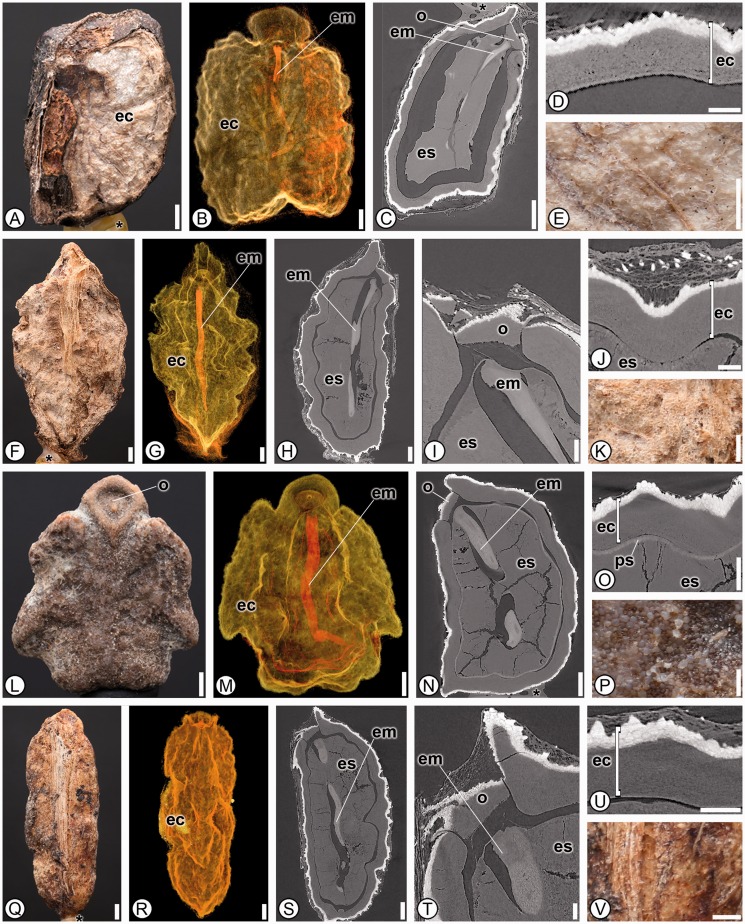
Seed anatomy in Heliconiaceae. (A, E–F, K–L, P–Q, V): light micrographs; (B, G, M, R): SRTXM 3D volume renderings; (C, H–I, N, S–T): SRTXM digital longitudinal sections; (D, J, O, U): SRTXM digital transverse sections. (A–E) *Heliconia velutina*. (A) Overview of pyrene with rugose tan endocarp (ec). (B) General shape of endocarp (ec) with J-shaped embryo (em). (C) Internal morphology of pyrene with operculum (o), embryo (em) and endosperm (es). (D) Endocarp (ec) with three distinctive layers. (E) Detail of ruminate tan endocarp surface with veins. (F–K) *Heliconia bihai*. (F) Overview of pyrene with tan, ruminate endocarp. (G) General shape of endocarp (ec), with embryo (em). (H) Internal morphology pyrene with embryo (em) and endosperm (es). (I) Micropylar region with multilayered operculum (o), embryo (em) and endosperm (es). (J) Detail of fruit wall with endocarp (ec) above endosperm (es). (K) Detail of ruminate endocarp surface. (L–P) *Heliconia griggsiana*. (L) Overview of pyrene with ruminate, grey endocarp and operculum (o). (M) General shape of endocarp (ec) with J-shaped embryo (em). (N) Internal morphology of pyrene with operculum (o), embryo (em) and endosperm (es). (O) Detail of endocarp layer (ec) above perisperm (ps) and endosperm (es). Seed coat is poorly developed and not distinguishable. (P) Detail of ruminate endocarp surface. (Q–V) *Heliconia papuana*. (Q) Overview of pyrene with ruminate, light brown endocarp. (R) General shape of endocarp (ec). (S) Internal morphology of pyrene with embryo (em) and endosperm (es). (T) Micropylar region with multilayered operculum (o), embryo (em) and endosperm (es). (U) Detail of fruit wall with endocarp (ec). (V) Detail of ruminate endocarp surface. * indicates mounting glue and/or specimen stub. Scale bars: A, F–H, J, L–M, P–R = 1 mm; B, I, K, R–S = 500 µm; C, N = 250 µm; D–E, O, T = 100 µm; U = 50 µm.

***Heliconiaceae*.** Seventeen species from the single genus *Heliconia* were analyzed and 35 character states were found in common in all taxa (Fig. 4A–V). Seeds can be differentiated from the other families by having a thick, sclerified endocarp surrounding the seed (1; Fig. 4C–D, J, O and U). The thickened sclerified endocarp has been misinterpreted by authors previously as seed coat (Rodríguez-de la Rosa and Cevallos-Ferriz, 1994; Panja and Maiti, 2012), but also correctly interpreted by others and our personal observations of *Heliconia vellerigera* (Simão *et al.*, 2006, and J.C. Benedict and S.Y. Smith pers. obs.). Additional characters to help differentiate Heliconiaceae from the other families include a ruminate surface (3; Fig. 4A, F, L and Q), a distinctive multilayered operculum that is located off-center on the proximal side of the seed (15, 16 and 17; Fig. 4C, I, N and T), and always lacking an aril or any evidence of arillate tissue (5; Table 3).

**Figure 5:**
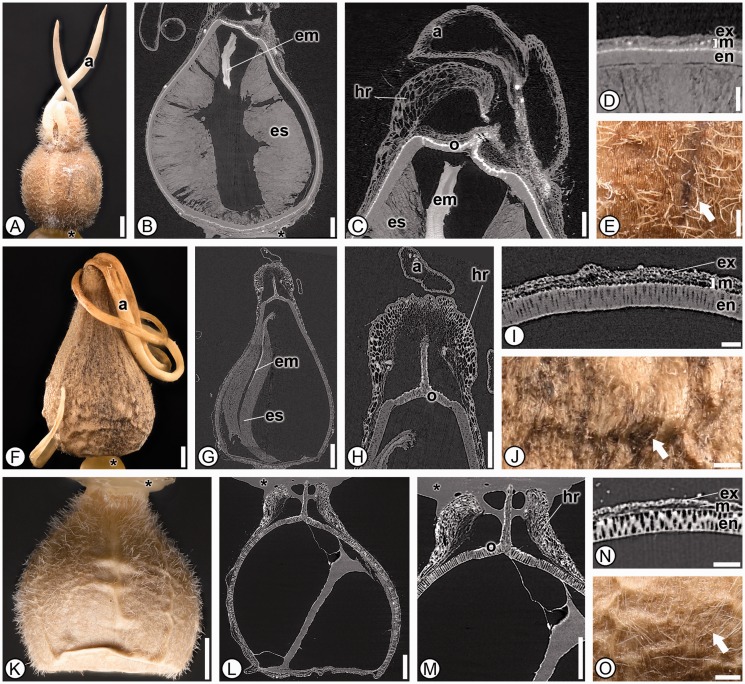
Seed anatomy in Lowiaceae. (A, E–F, J–K, O): light micrographs; (B–C, G–H, L–M): SRTXM digital longitudinal sections; (D, I, N): SRTXM digital transverse sections. (A–E) *Orchidantha maxillarioides*. (A) Overview of light brown seed covered in trichomes with two-stranded thick lobed aril (a). (B) Internal morphology showing embryo (em) and endosperm (es). (C) Micropylar region with aril (a), multilayered operculum (o), hilar rim (hr) of mesotesta and exotesta, embryo (em) and endosperm (es). (D) Seed coat with isodiametric exotesta (ex), mesotesta (m) of two cell types and elongate, sclerenchymatous endotesta (en). (E) Detail of striate seed surface with numerous trichomes (one at arrow). (F–J) *Orchidantha sabahensis*. (F) Overview of light brown seed with thick lobed aril (a). (G) Internal morphology showing embryo (em) and endosperm (es). (H) Micropylar region with single layered operculum (o), hilar rim (hr) of exotesta and mesotesta and aril (a). (I) Seed coat with isodiametric exotesta (ex), mesotesta (m) of two cell types and elongate, sclerenchymatous endotesta (en). (J) Detail of striate and verrucose seed surface with trichomes (arrow). (K–O) *Orchidantha vietnamica*. (K) Overview of tan seed. (L) Internal morphology; no embryo or nutritive tissues were present. (M) Micropylar region with hilar rim (hr) of exotesta and mesotesta and single layered operculum (o). (N) Seed coat with isodiametric exotesta (ex), mesotesta (m) and endotesta (en) of elongate sclerenchyma. (O) Detail of striate and verrucose seed surface with trichomes. * indicates mounting glue and/or specimen stub. Scale bars: A, F, G, K = 1 mm; B, H, L, M = 500 µm; C, E, J, O = 250 µm; D, I, N = 100 µm.

***Lowiaceae*.** Four species from the single genus *Orchidantha* in Lowiaceae were available for study and although no single character was found to be unique to seeds of Lowiaceae, 30 characters states were shared between all members examined (Fig. 5A–O). Furthermore, the combination of the following character states is unique to Lowiaceae seeds: trichomes present on seed coats (4), arils that are present only at the micropyle and made of a few thick lobes (5), the absence of a micropylar collar (18), the absence of a chalazal chamber (27) and a seed coat composed of only outer integument (31; Table 3).

**Figure 6:**
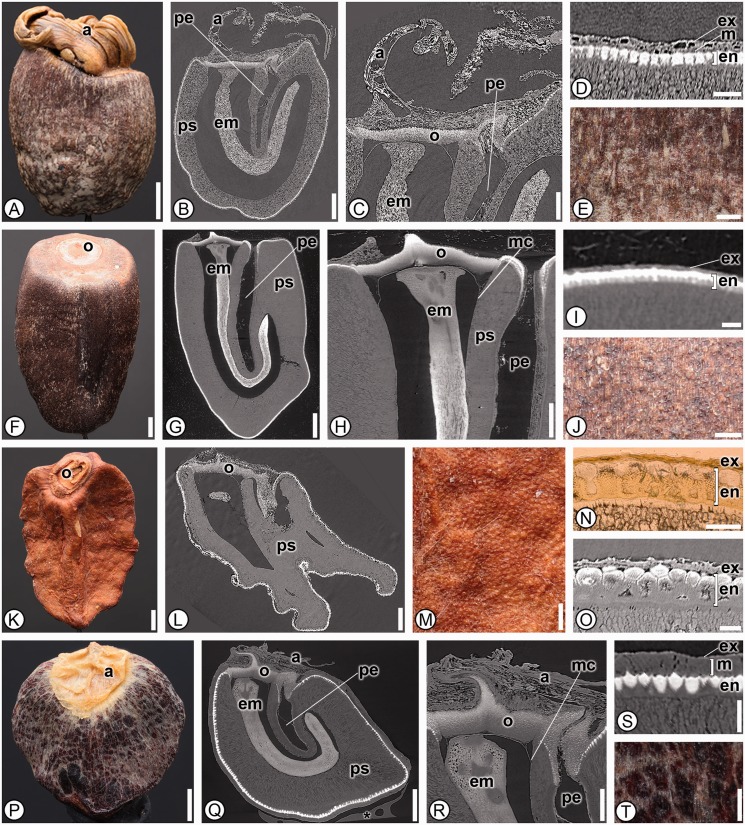
Seed anatomy in Marantaceae. (A, E–F, J–K, M–N, P, T): light micrographs; (B–C, G–H, L, Q–R): SRTXM digital longitudinal sections; (D, I, O, S): SRTXM digital transverse sections. (A–E) *Ctenanthe lanceolata*. (A) Overview of seed with aril (a). (B) Internal morphology showing aril (a), embryo (em) and perisperm canal (pe) within perisperm (ps). (C) Micropylar region showing aril (a), homogenous operculum (o), embryo (em) and perisperm canal (pe). (D) Seed coat with isodiametric exotesta (ex), mesotesta of a single cell type (m) and endotesta of elongate sclerenchyma (en). (E) Detail of seed surface. (F–J) *Calathea inocephala*. (F) Overview of dark brown seed with operculum (o). (G) Internal morphology showing J-shaped embryo (em) and perisperm canal (pe) within perisperm (ps). (H) Micropylar region with homogenous operculum (o), micropylar collar (mc), embryo (em) and perisperm canal (pe) within perisperm (ps). (I) Detail of seed coat with poorly developed exotesta (ex) and endotesta (en) of elongate sclerenchyma. (J) Detail of verrucose seed surface. (K–O) *Donax canniformis*. (K) Overview of rugose, reddish seed with off-center operculum (o). (L) Internal morphology showing homogenous operculum (o) and abundant perisperm (ps). (M) Detail of rugose seed surface. (N, O) Seed coat with poorly developed exotesta (ex) and endotesta (en) of elongate sclerenchyma. (P–T) *Marantochloa leucantha*. (P) Overview of dark brown, smooth seed with aril (a). (Q) Internal morphology showing aril (a), homogenous operculum (o), J-shaped embryo (em) and perisperm canal (pe) within perisperm (ps). (R) Micropylar region with aril (a), homogenous operculum (o), micropylar collar (mc), embryo (em) and perisperm canal (pe). (S) Seed coat with poorly developed exotesta (ex), mesotesta (m) of a single cell type and elongate, sclerenchymatous endotesta (en). (T) Detail of seed surface. * indicates mounting glue and/or specimen stub. Scale bars: A–B, F–G, K–L, P = 1 mm; C, H, Q = 500 µm; E, J, M, R = 250 µm; D, I, N–O, S–T = 100 µm.

**Figure 7:**
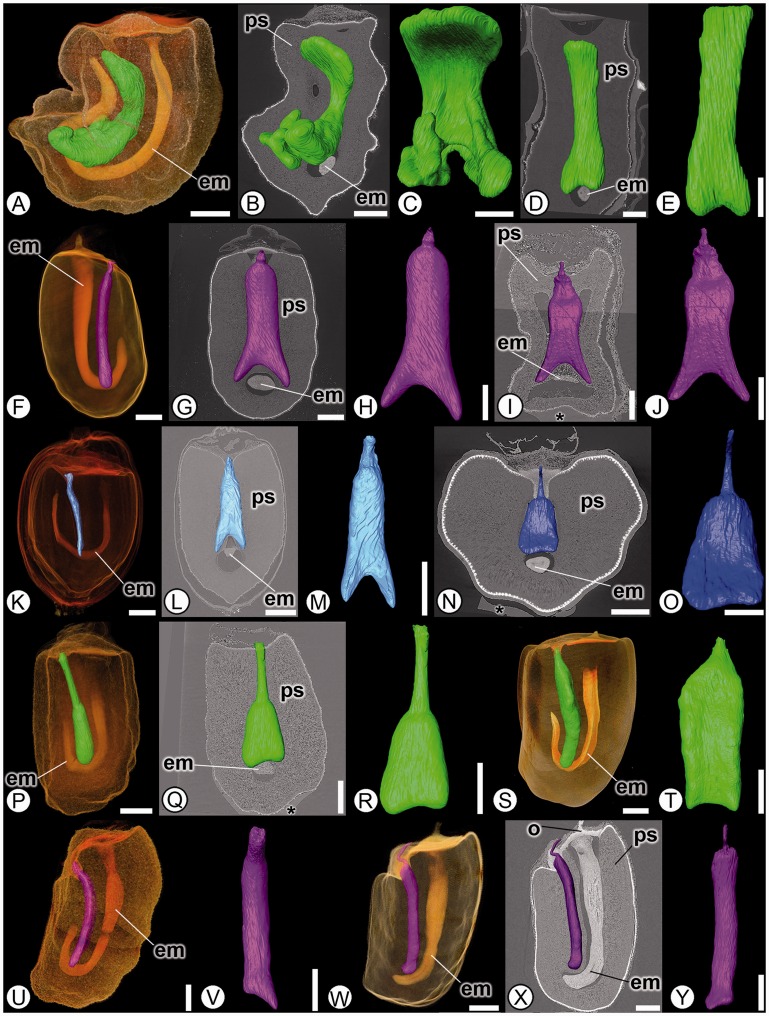
Perisperm canal variation in Marantaceae. (A, F, K, P, S, U, W): SRTXM 3D volume renderings; (B, D, G, I, L, N, Q, X): SRTXM digital longitudinal sections. (C, E, H, J, M, O, R, T, V, Y): SRTXM digital infills. Perisperm canals are infilled either green, fuchsia or blue. (A–C) *Sarcophrynium brachystachym* (*Sarcophrynium* clade) showing basally branched digitally infilled perisperm canal above embryo (em) and nested within perisperm (ps). (D–E) *Sarcophrynium prionogonium* (*Sarcophrynium* clade) showing basally bifurcated perisperm canal above embryo (em) and nested within perisperm (ps). (F–H) *Indianthus virgatus* (*Maranta* clade) showing basally bifurcated perisperm canal above embryo (em) and nested within perisperm (ps). (I–J) *Stromanthe stromanthoides* (*Maranta* clade) with basally bifurcated perisperm canal above embryo (em) and nested within perisperm (ps). (K–M) *Marantochloa leucantha* (*Stachyphrynium* clade) with basally bifurcated perisperm canal above embryo (em) and nested within perisperm (ps). (N–O) *Stachyphrynium sumatranum* (*Stachyphrynium* clade) with simple, unbranched perisperm canal above embryo (em) and nested within perisperm (ps). (P–R) *Calathea macrosepala* (*Calathea* clade) showing simple, unbranched perisperm canal above embryo (em) and nested within perisperm (ps). (S–T) *Calathea inocephala* (*Calathea* clade) with simple, unbranched perisperm canal above embryo (em) and within perisperm (ps). (U–V) *Phrynium* sp. (*Donax* clade) with simple, straight, unbranched perisperm canal. (W–Y) *Phrynium imbricatum* (*Donax* clade) showing simple, straight, unbranched perisperm canal above embryo (em) and nested within perisperm (ps). Scale bars: A–N, P–R, W–Y = 1 mm; O, S–V = 500 µm.

***Marantaceae*.** Twenty-three species from 20 genera were analyzed and found to share 31 character states and the presence of a perisperm canal (43) was found to be an autapomorphy for the family (Figs. 6A–T and 7A–Y). They also lack external chalazal indentations (12), are operculate (15), have thin, strongly recurved micropylar collars (18, 20 and 21), lack hilar rims (22), lack chalazal chambers (27) and chalazal pigment groups, which in combination also separates them from any other family in the order (Table 3).

The perisperm canal is one of the most striking anatomical features in Marantaceae seeds, and is quite variable in shape (Fig. 7A–Y). Schumann (1902) originally described two types of perisperm canals: branched and simple (unbranched). Andersson (1981) subsequently described three types: simple, branched at the base and branched more or less throughout the seed (also see Andersson and Chase [2001] for a larger sample of taxa). Our results are consistent with the three character states of Andersson (1981), although the branched-throughout state is much less common (Fig. 7A–E). When incorporated into the most recent published phylogeny of Marantaceae (Prince and Kress, 2006), it is clear that perisperm canal shape is homoplasious and is not apomorphic for any of the five informal clades proposed by Prince and Kress (2006). It should be noted, however, that the two sister clades, the *Calathea* clade (sensu Andersson and Chase [2001] and Prince and Kress [2006]) and *Maranta* clade (sensu Prince and Kress [2006], the combined *Maranta* clade and *Myrosma* clade of Andersson and Chase [2001]) are the only two clades with members with basally branched perisperm canals (Fig. 7F–J). Although perisperm canal shape is not a synapomorphic character state for any currently recognized clade in Marantaceae, the character is still a helpful morphological tool that could be used in tandem with other characters when formally revising relationships within the family (Fig. 7A–Y).

**Figure 8:**
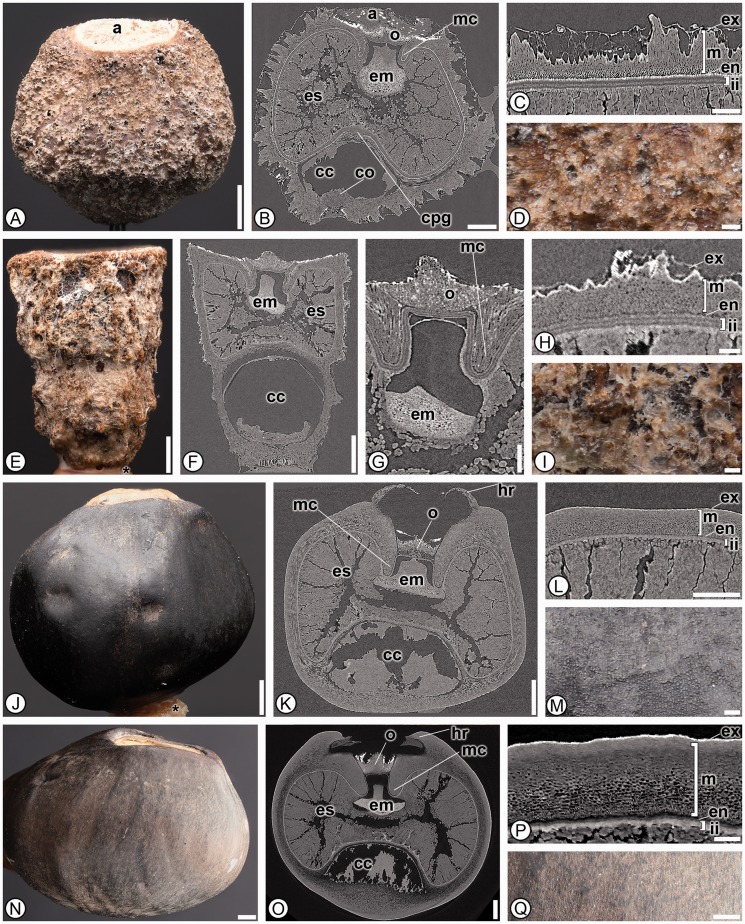
Seed anatomy in Musaceae. (A, D–E, I–J, M–N, Q): light micrographs; (B, F–G, K, O): SRTXM digital longitudinal sections: (C, H, L, P): SRTXM digital transverse sections. (A–D) *Musa textilis*. (A) Overview of dark brown, verrucose seed with aril (a). (B) Internal morphology showing aril (a), multilayered operculum (o), micropylar collar (mc), short bulbous embryo (em), endosperm (es), non-discoid chalazal pigment group (cpg), chalazal chamber column (co) and *Musa*-type chalazal chamber (cc). (C) Seed coat with poorly developed exotesta (ex), mesotesta (m) of two cell types, endotesta (en) of short sclerenchyma and inner integument (ii). (D) Detail of verrucose seed surface. (E–I) *Musa coccinea*. (E) Overview of light brown, rugose seed. (F) Internal morphology showing short, bulbous embryo (em), endosperm (es) and *Musa*-type chalazal chamber (cc). (G) Micropylar region with multilayered operculum (o), micropylar collar (mc) and embryo (em). (H) Seed coat with poorly developed exotesta (ex), mesotesta (m) of two cell types, short sclerenchymatous endotesta (en) and inner integument (ii). (I) Detail of rugose seed surface. (J–M) *Ensete lasiocarpum*. (J) Overview of black, smooth seed. (K) Internal morphology showing hilar rim (hr) formed from exotesta and mesotesta, multilayered operculum (o), short bulbous embryo (em), micropylar collar (mc), copious endosperm (es) and *Musa*-type chalazal chamber (cc). (L) Seed coat with poorly developed exotesta (ex), mesotesta (m) of two cell types, endotesta (en) and inner integument (ii). (M) Detail of smooth seed surface. (N–Q) *Ensete ventricosum*. (N) Overview of smooth, black-brown seed. (O) Internal morphology showing hilar rim (hr) formed from exotesta and mesotesta, multilayered operculum (o), micropylar collar (mc), short bulbous embryo (em), copious endosperm (es) and *Musa*-type chalazal chamber (cc). (P) Seed coat with poorly developed exotesta (ex), mesotesta (m) with two cell types, endotesta (en) and inner integument (ii). * indicates mounting glue and/or specimen stub. Scale bars: A–B, E–F, J–K, N–O = 1 mm; L, Q = 500 µm; C, G = 250 µm; D, H–I, M, P = 100 µm.

***Musaceae*.** The 17 species from the two genera of Musaceae shared 27 characters, one of which, a *Musa*-type chalazal chamber (27; a large, cylindrical or slightly conical chamber that can contain a mass of gelatinous cells) was found in all members of the family and no other taxa in the order, and is therefore considered an autapomorphy for the family (Fig. 8A–Q). Musaceae seeds also possess a conical micropylar region (14), a seed coat composed of both integuments (31), a raphe canal extending from the micropyle to the chalaza (42) and short, bulbous embryos (45, 47), which in combination are not found within any other group surveyed (Table 3). Interestingly, a chalazal chamber column (28), which was reported present in many previous studies on Musaceae seeds (McGahan, 1961; Bouharmont, 1963), was absent in *Ensete lasiocarpum, Ensete ventricosum*, *Musa peekelii*, *Musa sakaiana* and thus not diagnostic for distinguishing seeds within the family from other zingiberalean families.

**Figure 9:**
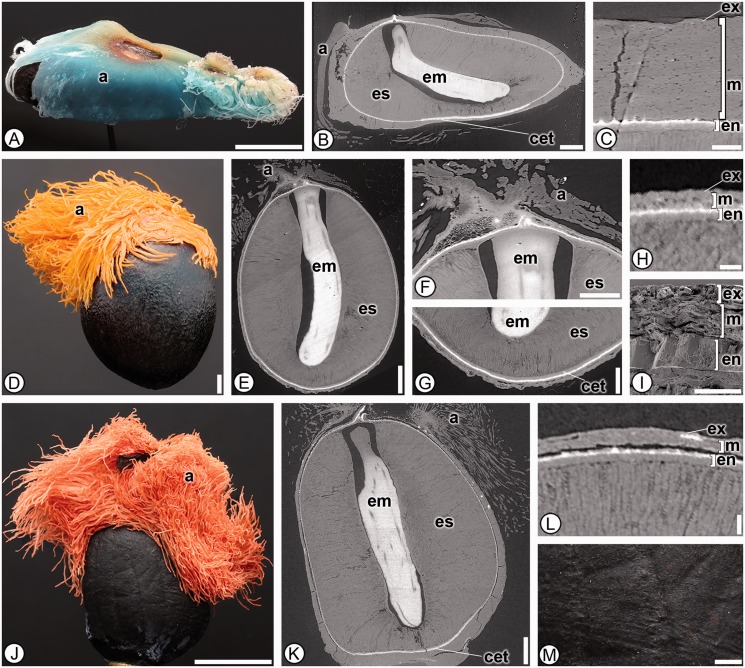
Seed anatomy in Strelitziaceae. (A, D, J, M) light micrographs; (B, E–G, K): SRTXM digital longitudinal sections; (C, H, L): SRTXM digital transverse sections; (I) SEM micrograph. (A–C) *Ravenala madagascariensis*. (A) Overview of black, rugose seed enveloped by blue aril (a). (B) Internal morphology showing aril (a), L-shaped embryo (em), copious endosperm (es) and chalazal endotestal thickening (cet). (C) Seed coat with isodiametric exotesta (ex), mesotesta (m) of one cell type and elongate sclerenchymatous endotesta (en). (D–I) *Strelitzia reginae*. Overview of black, striate seed with orange, hair-like aril (a). (E) Internal morphology showing aril (a), endosperm (es) and straight, elongate embryo (em). (F) Micropylar region with aril (a), embryo (em) and endosperm (es). (G) Chalazal region with embryo (em), endosperm (es) and chalazal endotestal thickening (cet). (H, I) Seed coat with isodiametric exotesta (ex), mesotesta (m) of one cell type and elongate sclerenchymatous endotesta (en). (J–M) *Phenakospermum guyannense*. (J) Overview of black, rugose seed with red-orange, hair-like aril (a). (K) Internal morphology showing aril (a), straight embryo (em), copious endosperm (es) and chalazal endotestal thickening (cet). (L) Seed coat with isodiametric exotesta (ex), mesotesta (m) and elongate sclerenchymatous endotesta (en). (M) Detail of black, rugose seed surface. Scale bars: A, J = 5 mm; B, D–E, K = 1 mm; F–G = 500 µm; C = 250 µm; H, L, M = 100 µm; I = 50 µm.

***Strelitziaceae*.** Four species representing all three genera in Strelitziaceae were analyzed and 29 character states were found to be shared among them, although none were found to be autapomorphic (Fig. 9A–M). Seeds are dark brown or black (2), lack trichomes (4), lack an operculum (15), lack a micropylar collar and chalazal chamber (18, 27) and have a conspicuously thickened endotesta at the chalaza (40) and this combination of character states is distinctive for the family. Strelitziaceae seeds are most similar to those of Lowiaceae in shape and the absence of a micropylar collar, but are easily differentiated as Strelitziaceae seeds also lack a hilar rim (22), an operculum (15), trichomes on their seed coats (4) and have a large conspicuous and colorful (red, blue or orange) aril.

**Figure 10:**
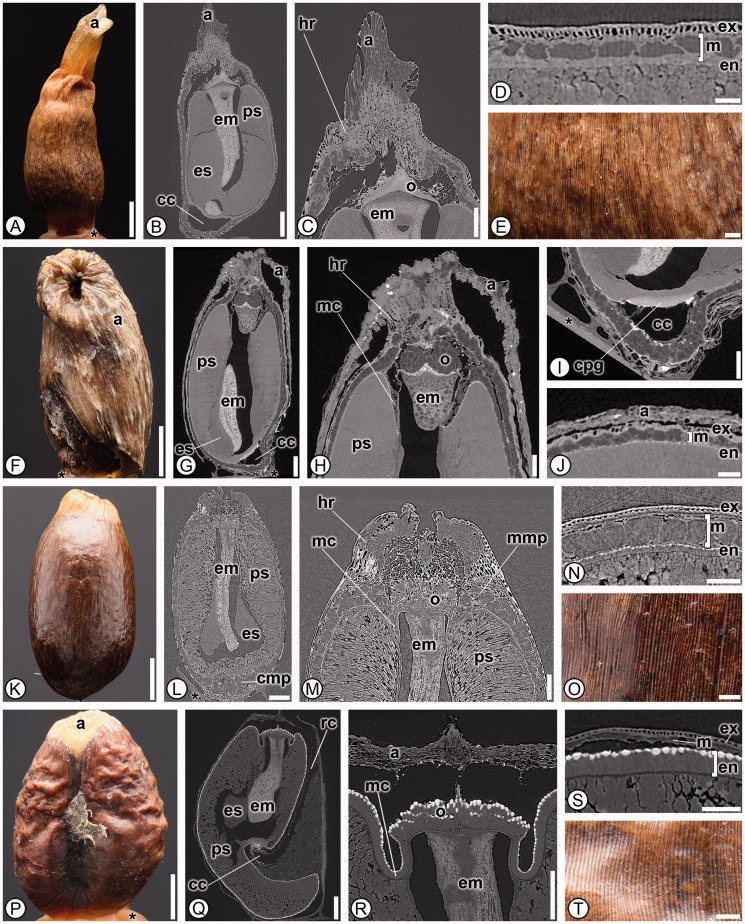
Seed anatomy in Zingiberaceae. (A, E–F, K, O–P, T): light micrographs. (B–C, G–I, L–M, Q–R): SRTXM digital longitudinal sections. (D, J, N, S): SRTXM digital transverse sections. (A–E) *Siphonochilus aethiopicus*. (A) Overview of striate, light brown seed with solid aril (a). (B) Internal morphology showing solid aril (a), embryo (em), perisperm (ps), basally proliferated endosperm (es) and *Amomum*-type chalazal chamber (cc). (C) Micropylar region with aril (a), hilar rim (hr) of exotesta and mesotesta, homogenous operculum (o) and embryo (em). (D) Seed coat with palisade exotesta (ex), mesotesta (m) of two cell types and thin parenchymatous endotesta (en). (E) Detail of striate surface. (F–J) *Zingiber larsenii*. (F) Overview of dark brown seed with tan aril covering a majority of seed (a). (G) Internal morphology showing aril (a), embryo (em), perisperm (ps), basally proliferated endosperm (es) and *Alpinia*-type chalazal chamber (cc). (H) Micropylar region with aril (a), hilar rim (hr), multilayered operculum (o), micropylar collar (mc), perisperm (ps) and embryo (em). (I) Chalazal region with discoid chalazal pigment group (cpg) and *Alpinia*-type chalazal chamber (cc). (J) Seed coat with non-uniform palisade exotesta (ex), mesotesta (m) of two cell types and thin parenchymatous endotesta (en), with aril (a). (K–O) *Aframomum angustifolium*. (K) Overview of brown, striate seed with the aril removed. (L) Internal morphology showing embryo (em), perisperm (ps), basally proliferated endosperm (es) and chalazal mesotestal proliferation of cells (cmp). (M) Micropylar region with hilar rim (hr) of exotesta and mesotesta, multilayered operculum (o), micropylar collar (mc), perisperm (ps) and embryo (em). (N) Seed coat with palisade exotesta (ex), mesotesta (m) of three layers and endotesta (en) of short, sclerenchymatous cells. (O) Detail of striate seed surface. (P–T) *Amomum sericeum*. (P) Overview of reddish-brown seed with remnant of aril (a). (Q) Internal morphology showing embryo (em), perisperm (ps), basally proliferated endosperm (es), raphe canal (rc) and *Amomum*-type chalazal chamber (cc). (R) Micropylar region with aril (a), micropylar collar (mc), homogenous operculum (o) and embryo (em). (S) Seed coat with palisade exotesta (ex), mesotesta (m) of one cell type and endotesta (en) of elongate sclerenchyma. (T) Detail of striate and verrucose surface. * indicates mounting glue and/or specimen stub. Scale bars: A, F, K, P = 1 mm; B, G, L, Q = 500 µm; C, H–I, M, R = 250 µm; E, J, N–O, S–T = 100 µm; D = 50 µm.

***Zingiberaceae*.** Eighty species from 33 genera from three of the four subfamilies (the monospecific Tamijioideae was not available for study) were found to be shared by all members of the family (Fig. 10A–T). Zingiberaceae seeds are remarkably diverse morphologically, and have been described in detail previously (Benedict *et al.*, 2015a, b; Fig. 10A–T). Although none of the characters found here are autapomorphic for Zingiberaceae, nine characters were found in all members of the family [Table 3 and **Supporting Information**—Table S2]. Seeds lack a sclerenchymatous endocarp (1), lack stomata (13), have a mechanical layer of outer integument only (31), the testa is not notably palisade in a small portion of the chalaza (35) and seeds lack a perisperm canal (43) and, in combination, these five characters are unique to Zingiberaceae (Table 3). Seeds of Costaceae are the most similar to those of Zingiberaceae with respect to the character states described above, but differ in having a *Costus*-type chalazal chamber (27; Fig. 3C, M and R), and always having a small portion of the seed coat that is distinctly palisade at the chalaza (35).

### NMDS analyses

**Figure 11:**
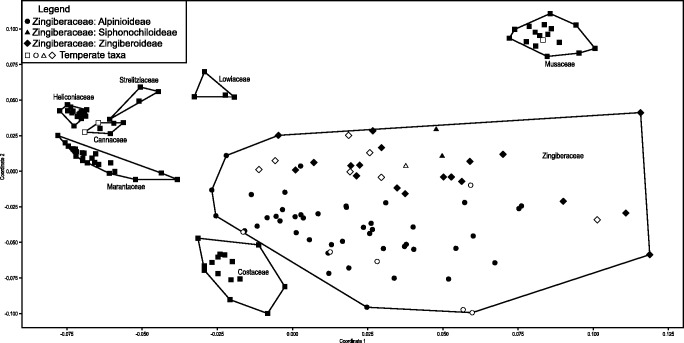
Non–metric multidimensional scaling analysis using Gower’s similarity index, based on 51 seed characters. Note each family occupies a distinct area of seed morphospace. Zingiberaceae occupy the largest region, and within Zingiberaceae, subfamilies generally cluster together, but temperate taxa are scattered.

Every family occupies a distinctive region of morphospace (Fig. 11). Cannaceae, Heliconiaceae and Lowiaceae occupy the smallest morphospace of the eight families, which reflects the generally limited morphological variation of seeds in these groups and the high number of characters shared by members of the families (i.e. Cannaceae: 36 shared characters, Heliconiaceae: 35 shared characters and Lowiaceae: 30 shared characters). Zingiberaceae occupy the largest region of morphospace, which is considerably larger than the combined space occupied by all other families. The subfamilies of Zingiberaceae are distributed mostly into two distinct groups, one that represents Alpinioideae and another that represents Zingiberoideae plus Siphonochiloideae; the latter two subfamilies have similar characteristics, including a thin, parenchymatous endotesta (37) and a conical micropylar region (14). Taxa found in both temperate and tropical regions were randomly distributed throughout the morphospace (Fig. 11).

***Environmental data extrapolation*.** Two versions of the GBIF georeferenced distributional data were analyzed, one with all available data on members of Zingiberales (exclusions as stated in ‘Materials’ section), referred to subsequently as ‘all data’, and a subset with only the species for which seed data were available in the current study, referred to subsequently as ‘subset’. Percentages reported are percent of occurrences for that family.

***All Zingiberales taxa*.** The most commonly occupied regions for all eight families were the tropics and subtropics [Table 2 and **Supporting Information**—Tables S3 and S4]. This region accounted for more than 90 % of all distribution data in all but Strelitziaceae and Zingiberaceae where tropical and subtropical occurrences accounted for 89 and 86 % of occurrences, respectively. Three families were found to be present in temperate regions and represented 17 % of all occurrence data: Cannaceae (1 %), Musaceae (4 %) and Zingiberaceae (12 %). The mangrove biome accounted for 8 % of all data, with 2 % of those occurrences in Cannaceae and Marantaceae, and 1 % in Costaceae, Heliconiaceae, Strelitziaceae and Zingiberaceae. Deserts and xeric shrublands accounted for 5 % of total distribution data [Cannaceae (2 %), Costaceae (1 %), Heliconiaceae (1 %), Marantaceae (1 %)]. Flooded grasslands and savannas accounted for 3 % of total distribution data (2 % in Cannaceae, and 1 % in Marantaceae), and montane grasslands and shrublands were 9 % of total distribution data (1 % in Cannaceae, 2 % in Musaceae, 5 % in Strelitziaceae and 1 % in Zingiberaceae).

Altitudinal, precipitation and temperature data were generally similar across the order with the exception of Lowiaceae, which had a much smaller range in all values (Table 2). All families except Lowiaceae and Strelitziaceae were found in altitudinal ranges from slightly below sea level (minimum value −17 m in Marantaceae and Zingiberaceae) to about 4600 m (maximum value 4849 m in Heliconiaceae), with average elevations from approximately 250–700 m. Average annual precipitation ranged greatly from 1747 mm in Cannaceae to 2540 mm in Heliconiaceae (Table 2). All families were similar in occupying areas where average maximum warmest month temperatures were between 29.4 ° C (Zingiberaceae) and 31.1 ° C (Marantaceae). Minimum coldest month data varied between families and both Lowiaceae and Strelitziaceae had no individuals occurring in regions that had a minimum coldest month with subzero temperatures. Zingiberaceae were found to occupy areas with the lowest minimum coldest month temperatures reaching −14.7 ° C.

***Subset of taxa available in the current study*.** In the subset, all families except Zingiberaceae were found in tropical and subtropical regions for 94 % or more of all occurrences. In Zingiberaceae, these regions accounted for only 64 % of the distribution data while temperate regions accounted for 34 % of occurrences (Table 2). Occurrences in non-tropical, non-temperate regions (i.e. deserts, mangroves, montane regions), accounted for less than 7 % in all families and followed a similar trend as the ‘all taxa’ sampled dataset, with the exceptions that Strelitziaceae were less commonly found outside the tropics and subtropics (only 6 % as compared to 11 %), and Musaceae were more pronounced in montane regions (5 % of subsampled dataset). Average altitudinal data, as with the entire dataset, were similar among families at 253–690 m elevation, with Cannaceae having the highest average altitude and Lowiaceae having the narrowest range (278–291 m; Table 2). Average annual precipitation between the families ranges from 1834 mm (in Cannaceae) to 2569 mm (in Heliconiaceae). All families were similar in average warmest month temperatures, which ranged from 28.5 ° C (Zingiberaceae) to 31.9 ° C (Marantaceae). Lowiaceae, Musaceae and Strelitziaceae were not found in any regions that experienced subzero temperatures, and Zingiberaceae were found in the coldest regions, with a minimum coldest month temperature of −12.4 ° C (Table 3).

## Discussion

### Seed structure in Zingiberales

The morphological and anatomical variation found in the seeds of Zingiberales is extraordinarily diverse and provides an opportunity to document a variety of characters not present in plant lineages with seeds of simpler construction. Additionally, the use of SRXTM allows for increased confidence in our interpretation of characters because the non-destructive nature of this methodology does not introduce artifacts or lose data, as is sometimes the case with traditional techniques (e.g. the identification of genuine spaces vs. artefacts; tissue mutilation or the use of hydrofluoric acid to soften seeds for microtomy, which dissolves silica bodies present in seed coats; see Smith *et al.* [2009] for a discussion). Non-destructive methods also allow for the sampling of rare specimens for which destructive sampling are prohibited (e.g. herbarium specimens, fossils). Thus SRXTM is an excellent technique to produce consistent datasets of similar quality to analyze character states for evaluating character evolution and phylogenetic relationships both within Zingiberales and for other groups of plants. Where sufficient material was available, multiple seeds per individual were analyzed using SRXTM and individual seeds were selected from the middle of the fruits as often the apical and basal seeds in Zingiberales fruits are contorted or aborted and could distort true character states of the taxon (J.C. Benedict and S.Y. Smith pers. obs.). In other monocot families, intraspecific variation of seeds occurs regularly (e.g. Cyperaceae, see Martinetto *et al.*, 2014; Commelinaceae, some genera with dimorphic seeds due to positioning of the operculum or embryotega, see Faden and Hunt, 1991), but this phenomenon does not occur in Zingiberales and of the 166 species analyzed here, seeds are notably uniform within a species.

Of the 51 characters analyzed for each taxon, 44 were phylogenetically informative, while seven were not informative in differentiating between the eight families and were found to be quite homoplasious [Tables 1 and 3; **Supporting Information**—Table S2]. In general, 19 characters had homoplasy indices (HI; Table 1) higher than 0.90, which demonstrates high homoplasy or character state disparity for seed characters in the order. The characters with the highest degree of homoplasy (HI 0.97) were seed body taper at the micropyle (9) and chalaza (10) and were distributed throughout the eight families [chalaza taper (10) absent in Lowiaceae] and not useful as synapomorphies for recognizing any clade. Seed shape [6, (HI) 0.92], notably contorted seeds (7, HI 0.96), seed length (8, HI 0.96), micropylar mesotestal proliferations of cells (24, HI 0.91) and perisperm canal shape (44, HI 0.67) were also often found to be variable within families and genera and not useful in differentiating amongst Zingiberales families (Table 1). In contrast, five character states were present in all members surveyed in individual families and are autapomorphies for these clades: *Musa*-type chalazal chambers in Musaceae (27), sclerified endocarps in Heliconiaceae (1), *Costus*-type chalazal chambers in Costaceae (27), presence of perisperm canals in Marantaceae (43) and stomata in seed coats in Cannaceae (13; Figs. 1 and 2E, J, O and T). These autapomorphies in combination with other phylogenetically informative seed characters (see earlier) are a novel source of data that can be used to distinguish the various families of the order [**Supporting Information**—Table S5], and they show that seeds are a rich source of morphological data that can be used to help understand the phylogenetic relationships within Zingiberales.

Across the order, Zingiberales seeds are highly variable in terms of their anatomical and morphological characteristics, and, as shown above, have suites of characters and apomorphies that can distinguish the various families. Interestingly, only Zingiberaceae have relatively few characters (nine) to unite the 80 species analyzed here, whereas the other seven families have 26–36 characters to unite them. When comparing the eight families using NMDS analysis (Fig. 11), each family occupies a distinctive region of morphospace, although the size of that space varies between groups. Cannaceae and Heliconiaceae occupied the smallest regions of morphospace based on seed/endocarp characters, reflecting the little variation in seed morphology in each family, that they have the largest number of shared seed characters (Cannaceae, 36 shared character states; Heliconiaceae, 35 shared character states), and each have a unique autapomorphy. In contrast, Zingiberaceae occupy the largest morphospace region compared to the other families and show the greatest morphological disparity among species.

### Quality of sampling

We observed very different patterns in seed structure diversity amongst the families, and differences in the breadth and position of seed morphospace each family occupies (see above), which lead to trying to understand what might be responsible for these different patterns amongst families. It is well known that homoplasy and/or character diversity increases in a clade as more taxa are included in a study (Sanderson and Donoghue, 1989; Wake, 1991; Foote, 1997). Because Zingiberaceae are three times more densely sampled than the other seven families that could lead to an overestimation of their diversity. However, proportional to the total species/genus diversity of each family, Zingiberaceae are not actually over-represented—in fact they are undersampled—and any sampling bias would have more likely led to an underestimation of Zingiberaceae seed diversity relative to the other families. Our taxon sampling ranges from 100 % of described genera (Cannaceae, Heliconiaceae, Lowiaceae, Musaceae and Strelitziaceae) to 61 % (Zingiberaceae), with 86 % of the genera sampled from Costaceae and 71 % in Marantaceae. Species level coverage is more variable, from 50–57 % (Cannaceae, Strelitziaceae), to 20–25 % (Lowiaceae, Musaceae), to 10 % or less in the other families, with the lowest sampling amongst the two most diverse families: 4 % of Marantaceae and 5 % of Zingiberaceae. In addition, while Marantaceae are the second largest family and proportionally represented similarly to Zingiberaceae, Marantaceae do not show nearly the same diversity in seed structure and occupy a smaller morphospace (Fig. 11).

### Ecology, diversity and habit

Presuming that the sampling has not biased our dataset (discussed above) we can ask if there are other factors—functional, ecological or structural—that might account for the relatively high levels of morphological disparity seen between seeds of Zingiberaceae species and the high species diversity in the family. Success of clades can be considered in terms of species diversity or morphological disparity, and this was addressed recently from an evo-devo perspective, with three main modes proposed to account for increases in these indicators (Minelli, 2015). Successful clades, like Zingiberaceae, with high diversity and high disparity could have come about due to: (1) increased evolvability (release of constraints or presence of genetic or developmental conditions favoring evolutionary transitions); (2) phenotypic plasticity; or (3) modularity, heterochrony or increased complexity in life cycles (Minelli, 2015). Given the data considered here, we cannot currently dismiss any of these factors, but we would suggest that Zingiberaceae most likely have an increased evolvability compared to the seven other families in the order, because of the remarkable variation in seed character states that exist for the group. Phenotypic plasticity is not likely to be the cause of high diversity among Zingiberaceae seeds due to the low variability detected within individuals or within species. Increased modularity or alternations in relative developmental timing (heterochrony) could contribute to the success of Zingiberaceae, but these avenues of diversity and disparity increase cannot be addressed with the dataset at hand. Future studies incorporating molecular and morphological data may be able to address the contribution of these modes to diversity and disparity in this group.

The ecological and geographical context of Zingiberales needs to also be addressed when considering the factors contributing to diversity and disparity within the group. It has been shown previously that Zingiberales are predominately pantropical in distribution, but are also found in subtropical and even temperate regions (Dahlgren *et al.*, 1985; Kress and Specht, 2005). Few detailed surveys exist about the ecological tolerances of members of the Zingiberales, but it is known that some species (e.g. *Roscoea*, *Cautleya*, *Alpinia*, *Canna* and *Thalia*) have geographic ranges that extend well into temperate zones (Kress and Specht, 2005) and that some Zingiberaceae can survive frost and can occupy high latitudes (e.g. *Alpinia japonica*, minimum temperature −12 °C; *Roscoea cautleyoides*, minimum temperature −25 °C, 2000–3500 m; *Zingiber mioga*, −23 °C; Branney, 2005). The ability of Zingiberaceae to repeatedly invade high-elevation and temperate environments (since such taxa are found in all three subfamilies surveyed here) could be a factor in it being the most successful family in terms of generic and species diversity. It has been demonstrated in other plant groups that species richness is strongly correlated with varied pollination and dispersal syndromes, growth forms, climate tolerances and ecological roles (Ricklefs and Renner, 1994; Givnish *et al.*, 2015), and this may well be the case in Zingiberaceae.

To compare climate disparity across the Zingiberales, occurrence data for the subset of taxa included in this study were analyzed for biome type and seven climatic variables [Table 2 and **Supporting Information**—Table S4]. In seven of the eight families, tropical biome distribution accounted for at least 94 % of total occurrences, whereas in Zingiberaceae, tropical biome occurrences only amounted to 64 % of total distribution data. Thirty-four percent of Zingiberaceae occurrences were found in temperate and montane regions, and two taxa (*Monolophus sikkimensis* and *Cautleya spicata*) are exclusively reported from these temperate regions. Within Zingiberaceae, species with distributions that include occurrences in temperate regions are found in all three subfamilies studied here (data for Tamijioideae not available for study), but only Zingiberoideae has species that are distributed exclusively in these regions. The all-Zingiberales dataset shows similar patterns to the subset, with Zingiberaceae having a notably larger proportion of individuals in temperate regions compared to the other families [12 % in Zingiberaceae, 4 % in Musaceae, 1 % in Cannaceae and <1 % or absent in Costaceae, Heliconiaceae, Lowiaceae, Marantaceae and Strelitziaceae; Table 2 and **Supporting Information**—Table S3]. These data show that Zingiberaceae are unique among the order in their expansion out of the tropics and subtropics and into more temperate regions in Asia.

When we examine which families have individuals in regions with a minimum coldest month temperature below freezing, Zingiberaceae are distinct from the other seven families. In Cannaceae, Costaceae, Heliconiaceae, Marantaceae, Musaceae and Zingiberaceae, each family has individuals that have been reported to grow in regions with minimum coldest temperatures below 0 ° C (Table 2). In all except Zingiberaceae these occurrences are a relatively small proportion of the total occurrence data and are all based on individuals that have been found on mountains (e.g. Andes, Himalayas) at relatively high altitudes. In contrast, almost 2 % of the Zingiberaceae records were located in subzero regions in both low and high altitudes (59–4624 m) in the Himalayas and in high latitudinal regions of China and Japan. It was proposed recently that some of this radiation into cooler environments could be explained by the diversification of a few Zingiberaceae genera into higher altitudes during the middle Eocene Himalayan-Tibetan Plateau uplift (Zhao et al., 2015). However, the occupation of the low altitudinal, high latitudinal regions is also an important component of the occurrence of the group in cooler environments and cannot be explained by uplift alone. It is clear that Zingiberaceae differ from all the rest of Zingiberales in occupying temperate regions and areas with subzero temperatures.

The species of Zingiberales that do occupy both temperate and tropical regions or temperate regions exclusively, do not appear to have any unique combination of seed characters, nor do they occupy one distinct region of morphospace (Fig. 11). Therefore, we cannot conclude that the high seed structural diversity in Zingiberaceae is a result of novel character states acquired through colonizing a new habitat, although there could be chemical or other differences that were not accounted for in the characters analyzed here. Possibly, the lability in seed characters reflects a general genetic lability, or evolvability in Zingiberaceae that allowed them to expand into these novel niches, which has been shown for several other species-rich families (Ricklefs and Renner, 1994; Givnish *et al.*, 2015). Indeed, Zingiberaceae are remarkably diverse in terms of growth forms as well, from epiphytes (e.g. *Hedychium* spp.), to aquatics (e.g. *Alpinia aquatica*), to vine-like and some that can achieve heights over ten meters (Williams *et al.*, 2003; Kress and Specht, 2005). The notable seed anatomical and morphological variety may account for the extraordinary ecological success and high species diversity of Zingiberaceae relative to the other seven families in the order.

## Conclusions

Zingiberales are a diverse order of monocots with high disparity in terms of seed morphoanatomical characters (19 characters with homoplasy indices higher than 0.90). Of the 51 characters analyzed here for 166 species in the order, five apomorphies were found that reinforce current familial relationships [*Musa*-type chalazal chambers in Musaceae (27, HI 0.87), sclerified endocarps in Heliconiaceae (1, HI 0.00), *Costus*-type chalazal chambers in Costaceae (27, HI 0.87), presence of perisperm canals in Marantaceae (43, HI 0.00) and stomata in seed coats in Cannaceae (13, HI 0.00)]. Families show distinctive seed structure recognizable based on a single or unique combination of characters and in NMDS analyses (Gower stress index = 0.2769). Within the order, Zingiberaceae were found to possess the most disparate combination of characters and occupied the largest morphospace, which reflects the wide range of character states and lability or evolvability of the family. Currently available distribution data showed that Zingiberaceae differ from the other seven, almost exclusively tropical, Zingiberales families, by inhabiting frost-prone regions at high altitudes and latitudes, which may account for its notably higher species diversity compared to the other families in the order. Furthermore, we propose that this lability or evolvability seen in seed morphoanatomy may reflect a general trend in genetic, ecological and habitat plasticity of Zingiberaceae that allowed for multiple independent radiations out of the tropics into cooler temperate environments and subsequent speciation events in these regions.

## Sources of Funding

This work was supported by a Heliconia Society International award to J.C.B. and National Science Foundation (USA) grants DEB 1257080 (S.Y.S) and 1257701 (C.D.S). Research of J.L.S. is supported by National Parks Board, Singapore and the Czech Science Foundation, GAČR P506–14–13541S. The research at TOMCAT beamline at the Swiss Light Source, Paul Scherrer Institut, Villigen, Switzerland received funding from Integrated Infrastructure Initiative (I3) on Synchrotrons and FELs and the European Community's Seventh Framework Programme (FP7/2007–2013) under grant agreement No. 312284 (CALIPSO) through SLS to M.E.C. and S.Y.S. This research used resources of the Advanced Photon Source, a U.S. Department of Energy (DOE) Office of Science User Facility operated for the DOE Office of Science by Argonne National Laboratory under Contract No. DE–AC02–06CH11357. The Advanced Light Source is supported by the Director, Office of Science, Office of Basic Energy Sciences, of the U.S. Department of Energy under Contract No. DE–AC02–05CH11231.

## Contributions by the Authors

J.C.B., S.Y.S., C.D.S. conceived of the project, led the initial data compilation and coordinated the writing. J.C.B., S.Y.S., C.D.S., M.E.C., J.L.S. contributed data, ideas and assisted with writing the final manuscript. D.Y.P. and M.F. assisted with synchrotron data acquisition and with writing the final manuscript.

## Conflict of Interest Statement

None declared.

## Supplementary Material

Supplementary Data

## Supporting Information

The following additional information is available in the online version of this article —

**Notes S1**. Seed structural diversity.

**Table S1**. List of specimens sampled and voucher information.

**Table S2**. Distribution of seed characters within Zingiberales.

**Table S3**. Environmental data for all extant Zingiberales taxa.

**Table S4**. Environmental data for Zingiberales included in the current study.

**Table S5**. Key to Zingiberales seeds.
