# Supplementary material for: Species diversity driven by morphological and ecological disparity: a case study of comparative seed morphology and anatomy across a large monocot order
Source: AoB Plants. 2016 Oct 26;8:plw063. doi: 10.1093/aobpla/plw063 (PMC5091906; doi:10.1093/aobpla/plw063)
Supplement: Supplementary Data [file supp_8_plw063_index.html]

Supplementary Data 

# Species diversity driven by morphological and ecological disparity: a case study of comparative seed morphology and anatomy across a large monocot order

## Supplementary Data

files

- Supplementary Data - zip file
