# Supplementary material for: Species diversity driven by morphological and ecological disparity: a case study of comparative seed morphology and anatomy across a large monocot order
Source: AoB Plants. 2016 Oct 26;8:plw063. doi: 10.1093/aobpla/plw063 (PMC5091906; doi:10.1093/aobpla/plw063)
Supplement: Supplementary Data [file supp_plw063_suppl_data.zip › 16044R2 Benedict_et_al_Zingiberales_seeds_supporting_information_all.doc]

RESEARCH ARTICLE

**Species diversity driven by morphological and ecological disparity: a case study of comparative seed morphology and anatomy across a large monocot order**

**SUPPORTING INFORMATION**

**Notes S1. Seed structural diversity**

All seeds studied were mature and scored for 51 characters (Table S1) described in detail below. These characters are adapted and modified from the characters used in Benedict *et al.* (2015a, b) to account for the greater anatomical variation in seeds of Zingiberales.

**1. Sclerenchymatous endocarp:** In Heliconiaceae, the seed coat is not the functionally protective layer of the seed coat and instead a sclerified endocarp is present to protect the embryo (e.g., Fig. 2k-n, S3a-d, S3f-j). Thus, many of the seed coat characters below are not applicable to these seeds (e.g., exotesta shape). Noting this very different diaspore construction, this family was scored for as many characters as possible. 0, absent; 1, present.

**2. Natural seed/ endocarp color:** Zingiberales seeds, when mature and dry, are often tan, red, or light brown (e.g., Fig. 3l, S7a, S7e, S5k), but can also be a dark brown to black (e.g., Fig. 3p, S7j, S8d, S8j), or even white. Character states are scored as follows: 0, white; 1, tan/ red/ light brown; 2, dark brown/ black.

**3. Seed surface:** The surfaces of Zingiberales seeds can be smooth, striate, verrucose (surfaces with small bumps), rugose (distinctly wrinkled surfaces), ruminate (rough, strongly wrinkled surface, like many *Heliconia*). Character states are scored as follows: 0, smooth; 1, striate; 2, verrucose; 3, rugose; 4, ruminate.

**4. Trichomes:** Trichomes may or may not be present on the surface of the seed coat or aril Fig. 2t, S4a, S4e, S4f, S4j, S4k, S4o). Character states are scored as follows: 0, absent; 1, present.

**5. Aril:** Arils and various fleshy, tubular, or disk-like appendages at the micropylar region of the seed have been given various names based on the particular region of the seed or funiculus from which they derive (Kapil *et al.* 1980). A detailed history and alternate classification is given by Kapil *et al.* (1980), in which they propose : in agreement with Corner (1976) : that all aril-like structures be called arils and terms such as arilloid, arillode, false aril, hilar aril, etc., be abandoned. They also propose that funicular arils, caruncles (derived from the exotesta, also called an exostome-aril), and strophioles (derived from the raphe tissue) be retained for descriptive purposes only. We adopt this interpretation of the definition of an aril, and choose to describe arils in terms of presence and absence without making a distinction of exact origin (integumentary, funicular, or raphe tissue) due to the limited developmental understanding of many taxa within the family. While the origin of the aril is not always clear, the extent of the aril may be useful in distinguishing between taxa. Arils may be solid structures confined to the micropylar end of the seed, may consist of many separate strands or a few thick lobes at the micropylar end; or may envelope more than half of a seed and be tightly adpressed, or not, to the seed coat. Character states are scored as follows: 0, absent; 1, present only at micropylar end of seed (e.g., Fig. 4a); 2, present covering part or most of seed, lacinate or not, easily separated from dry seeds (e.g., Fig. 4f); 3, present covering entire seed, often slimy or juicy when fresh and as a thin layer on dried material (e.g., Fig. S9p)

**6. and 7. Seed shape:** Seeds in Zingiberales vary considerably in their shape due, in part, to frequently tight packing within the fruit (J.C. Benedict and S.Y. Smith pers. obs.). Therefore, seed shape was documented for those seeds located closest to the middle of each fruit, showing the least compression.

**6. General seed shape:** Character states are scored as follows: 0, ellipsoid; 1, ovoid; 2, oblate (flattened at the poles of the seed); 3, polyhedral; 4, irregular.

**7. Seed contorted from arrangement in fruit:** Character states are scored as follows: 0, no contortion of seeds from tight packing in fruit (e.g., Fig. 2p, S4a); 1, seed shape contorted by tight packing in fruit (e.g., Fig. S2a).

**8. Seed length:** Seed length also varies with respect to location within the fruit and a binary character of “at least twice as long as wide” or “less than twice as long as wide” was used to generalize seed length. In seeds with large arils (e.g., *Orchidantha*, *Strelitzia*), the measurement was taken without the aril. Character states are scored as follows: 0, less than twice as long as wide; 1, at least twice as long as wide.

**9. Seed body taper at micropylar region:** In some seeds, the body has a slight decrease in width, or taper, towards the micropylar region. Character states are scored as follows: 0, absent; 1, present.

**10. Seed body taper at chalazal region:** In some seeds, the body has a slight decrease in width, or taper, towards the chalazal region. Character states are scored as follows: 0, absent; 1, present.

**11. Externally visible raphe:** During maturation of the seed, the anatropous ovules of Zingiberales taxa may produce an externally visible groove or ridge in the seed coat corresponding to the position of the raphe in the mature seed (e.g., Fig. 2f, S2f, S9p). Character states are scored as follows: 0, absent; 1, present, a single groove or ridge; 2, present, two grooves or ridges.

**12. External chalazal indentation:** As with the external raphe groove, in some Zingiberales the chalazal region of the seed has a distinctive circular indentation, termed a "sunken chalaza" in *Costus* (Grootjen and Bouman 1981). It is unclear if this structure is homologous for all Zingiberales seeds and future developmental work is needed to understand the evolution of this trait across the order. Character states are scored as follows: 0, absent; 1, present.

**13. Stomata in seed coat:** Stomata may or may not be present on the epidermis of the seed coat (e.g., Fig. 2e, S1e, S1j). Character states are scored as follows: 0, absent; 1, present.

**14. Micropylar region shape:** In longitudinal section, the micropylar region, sometimes including a hilar rim, operculum, and micropylar mesotestal proliferation of cells, can range from being conical, cylindrical, or not clearly defined or absent. Character states are scored as follows: 0, absent/not clearly defined; 1, conical; 2, cylindrical.

**15. Operculum:** An operculum is found within many Zingiberales and is conical to disk-shaped in longitudinal section. In Heliconiaceae the operculum is derived from endocarp tissue and not integumentary in origin like other Zingiberales. Character states are scored as follows: 0, absent; 1, present.

**16. Operculum layering:** The operculum is derived from mesotesta, endotesta, and/or inner integument, making them either homogeneous, or formed of two (or more) distinctive layers. In SRXTM images, the inner, (often endotesta-derived) layer often has an outer X-ray bright layer that forms a boundary with the outer mesotestal-derived layer; this varies from being extremely thin (e.g., Fig. 4h, S9h), to more substantial (e.g., Fig. 4m, S9r). An outer layer from mesotesta tends to be formed of larger cells, sometimes with intercellular spaces, and lacks the X-ray bright nature in SRXTM images (e.g., Fig. 4h, S9h). Character states are scored as follows: (–) no operculum present; 0, more or less homogeneous; 1, multilayered.

**17. Operculum position:** The operculum is in two different positions in Zingiberales seeds. It is commonly positioned at the centered and at the apex of the seed (e.g., Fig. 2b, S1b, S1f, S1k), but sometimes it is notably off center and present sub apically (e.g., Fig. 2m, S4c, S4h, S4t). Character states are scored as follows: (–) no operculum present; 0, more or less apical and centered; 1, subapical and off center.

**18. Micropylar collar:** The micropylar collar (labelled “MC” in figures) is a tube or cylinder of testal cells (see character 19) that expands into the embryo chamber, often creating two ‘v’ shapes below the operculum in longitudinal section (e.g., Fig. 2m, S7b). Some seeds appear to have shallowly or deeply infolded micropylar collars, but this character was found to be subjective in nature and thus was not included as a separate character. The apical portion of the micropylar collar is the attachment point for the operculum of many Zingiberales seeds and often surrounds the apical portion of the embryo. Character states are scored as follows: 0, absent; 1, present.

**19. Micropylar collar layering:** The micropylar collar is formed either from the endotesta or a combination of the endotesta and mesotesta. Liao and Wu (1996) recognized three types of micropylar collars based on large-volume mesotestal cells (“form A”), small-volume mesotestal cells (“form B”) or no mesotestal cells (“form C”). In our studies, the distinction of large and small mesotestal cells were found to be based on subtle differences; therefore two are recognized here, those that are formed from mesotesta and endotesta (=forms A or B) and those that are formed from endotesta only (=form C). Character states are scored as follows: (–), no micropylar collar present; 0, formed from endotesta only (e.g., Fig. 4m, S9r); 1, formed from endotesta and additional layers (e.g., Fig. S7g).

**20. Thickened micropylar collar:** The micropylar collar sometimes shows a distinctly thickened mesotesta in longitudinal section with respect to the rest of the seed coat. Character states are scored as follows: (–) no micropylar collar present; 0, absent; 1, present.

**21. Recurved micropylar collar:** The inner terminus of the micropylar collar ranges from being distinctly acute (strongly recurved) to weakly recurved, in longitudinal section. Character states are scored as follows: (–) no micropylar collar present; 0, weakly recurved (e.g., Fig. S7g); 1, strongly recurved (e.g., Fig. S9h).

**22. Hilar rim:** A hilar rim (labelled “HR” in figures) is an elongated tube of seed coat that forms a rim at the micropylar region of the seed (e.g. Fig. 2r, 4c, 4h, S7k). In longitudinal section it was previously described as “[it] produces the appearance of a pair of horns arising from the hilar end of the seed” (Manchester and Kress 1993: p. 1267). In some specimens the rim recurves slightly inwards, especially when the aril has been detached. Character states are scored as follows: 0, absent; 1, present.

**23. Hilar rim layering:** The hilar rim can be formed from the exotesta or a combination of the exotesta and mesotesta. Character states are scored as follows: (–) no hilar rim present; 0, formed from exotesta; 1, formed from exotesta and mesotesta.

**24. Micropylar mesotestal proliferation:** The mesotesta of the micropylar region of the seed may have a proliferation of cells to produce a mass of cells in the shape of a donut or cylinder (labelled “MMP” in figures; e.g., Fig S9m). In longitudinal section this proliferation of cells is adjacent to the operculum, above and offset from the micropylar collar. Character states are scored as follows: 0, absent; 1, present.

**25–27. Chalazal modifications:** Chalazal modifications of Zingiberales seeds are divided into two general forms: testal proliferations (masses of mesotestal cells that have undergone extra periclinal divisions in the chalazal region compared to the rest of the seed coat and contribute three or more rows of cells to the seed coat; characters 25 and 26) and chalazal chambers (empty cavities nested within the mesotesta of seed coats; character 27). Testal proliferations do not include raphe and chalazal pigment group cells. Two types of testal proliferations exist: i) a simple mass of mesotestal cells (labeled “CMP” in figures; e.g., Fig. S6l), here termed massive, and ii) a wall or column of endotestal and mesotestal cells that vertically divides the lower portion of the embryo cavity into two segments, termed here a columnar mesotestal proliferation. These two chalazal modifications are not mutually exclusive and taxa can have both a chalazal chamber and a proliferation of mesotestal cells.

**25. Massive chalazal testal proliferations:** Character states are scored as follows: 0, absent; 1, present.

**26. Columnar chalazal testal proliferations:** Character states are scored as follows: 0, absent; 1, present.

**27. Chalazal chamber:** Four distinct types of chalazal chambers (labelled “CC” in figures) have been identified in Zingiberales seeds, the *Alpinia*-type, the *Amomum*-type, the *Musa*-type, and the *Costus*-type. The *Alpinia*-type is typically lens-shaped and less than 1/3 the width of the seed (e.g., Fig. 4g, S9g) whereas the *Amomum*-type is more than 1/3 the width of the seed and often connects to (and becomes continuous with) the raphe canal in the seed (e.g., Fig. 4l, S9q). The *Costus*-type is circular or square in LS and is tightly surrounded by the endotesta (e.g. Fig. 2g, S2c, S2m, S2r). The *Musa*-type chamber is very large, cylindrical or slightly conical, and sometimes contains a mass of gelatinous cells (e.g., Fig. 3m, S7b, S7f; McGahan 1961). It is unclear whether these are homologous structures and further developmental studies are needed on this structure for the group. Character states are scored as follows: 0, absent; 1, *Alpinia*-type; 2, *Amomum*-type; 3, *Musa*-type; 4, *Costus*-type.

**28. Chalazal chamber column:** A column of sclerified cells projecting into the chalazal chamber may be present (labelled “CO” in figures) and has been described as a “bourrelet sclérifié au centre du disque inférieur” (sclerotized ridge in the center of the lower disk, Bouharmont 1963) or a central core of parenchyma in the chalazal mass (McGahan 1961). Character states are scored as follows: 0, absent; 1, present.

**29. Chalazal mucro:** A chalazal mucro– an abrupt, pointed termination of the seed (in contrast to character 10, which is a gradual tapering of the seed body) – was reported by Ridley (1909) where he suggested the structure (termed “terminal mucro”) was a modification for water and wind dispersal in *Burbidgea* Hook.f. It has since also been found in other Alpinioideae as well (Benedict *et al.* 2015a, b). The structure is composed of endotesta, mesotesta, and exotesta. Character states are scored as follows: 0, absent; 1, present.

**30. Mechanical layer thickness:** The mechanical layer of the diaspore (either endocarp or integumentary in origin) thickness is determined in transverse sections in the middle of the seed and is measured at the thinnest region of the seed coat. Character states are scored as follows: 0, 1–99 µm; 1, 100–199 µm; 2, ≥200 µm.

**31. Mechanical layer:** The layer(s) of the diaspore that contribute to the protection of the embryo. Character states are scored as follows: 0, outer integument; 1, inner and outer integument; 2, endocarp.

**32. Exotesta cell type:** The exotesta can be made of palisade cells, generally isodiametric cells, be poorly developed, or it can be absent. Character states are scored as follows: 0, palisade; 1, more or less isodiametric or cuboidal; 2, poorly developed or destroyed in mature seed.

**33. Uniform exotesta:** The exotesta is most commonly composed of a homogeneous layer of cells, but it can also be heterogeneous with cells that vary in shape as a result of irregular anticlinal divisions of the exotesta. Character states are scored as follows: 0, homogeneous; 1, heterogeneous.

**34. Multiseriate exotesta:** Previously described as a multiple epidermis by some authors (e.g., Wu and Liao 1995; Liao and Wu 2000), the exotesta is often uniseriate, but can be multiseriate with two or more cell layers. Character states are scored as follows: 0, absent; 1, present.

**35. Portion of exotesta palisade at chalaza:** In some seeds the exotesta is notably palisade at the chalaza but not in other regions of the seed (labelled “EPC” in figures; Fig. S2m). It was originally described by McGahan (1961) as a persistent epidermal fragment in *Musa balbisiana* Colla but has since been observed in other Zingiberales families (e.g., Fig. S2r). Character states are scored as follows: 0, absent; 1, present.

**36. Number of types of mesotestal cells:** The mesotesta, when differentiated, is composed of three cell types occurring in distinct layers in Zingiberales seeds (Liao and Wu 1996, 2000). These layers have been previously described as the hypodermis (directly beneath the exotesta), the translucent cell layer (beneath the hypodermis) and the pigment layer (beneath the translucent cell layer and above the endotesta). In some taxa, the three types can be discerned, whereas in others it is either absent, a single type, or two types. Character states are scored as follows: 0, absent; 1, one type; 2, two types; 3, three types.

**37. Endotestal cell thickness and shape:** The endotesta is the innermost layer of cells of the seed coat in most Zingiberales seeds. Its thickness and shape vary considerably and can range from very small square to rectangular sclerified cells that are less than 30 µm in thickness, to palisade sclerified cells 30 µm or greater in thickness, to a thin layer, <15 µm, of parenchyma cells. Character states are scored as follows: 0, absent; 1, thin parenchyma (<15 µm thickness); 2, short sclerenchyma (15–30 µm in thickness); 3, elongate sclerenchyma (≥30 µm in thickness).

**38. Endotestal gap location:** The endotesta has a small circular to ellipsoid interruption, often in the chalazal region of seed, and typically represents the point where the raphe terminates in the seed coat. In longitudinal section, this endotestal gap is filled by the chalazal pigment group so is not seen as a true void. The location of the gap varies from being at the base of the seed (e.g., Fig. 2r) to the side of the seed. Character states are scored as follows: 0, present at the chalazal end; 1, present on the side.

**39. Chalazal pigment group:** As noted in previous studies (Liao and Wu 1996, 2000), the chalazal pigment group (labelled “CPG” in figures; e.g., Fig. S9i) is a small collection of cells in the embryo cavity above the raphe and endotestal gap (e.g., Fig. 4i, S9i). Previously it was determined that members of Zingiberoideae have a discoid-shaped cpg, termed ‘crescent-shaped’ by Liao and Wu (2000), while members of Alpinioideae have 'trumpet-shaped' cpgs (Liao and Wu 2000) or otherwise non-discoid cpgs. Character states are scored as follows: 0, absent; 1, discoid-shaped; 2, non-discoid-shaped.

**40. Chalazal endotestal thickening:** In Strelitziaceae and some Lowiaceae, seeds have a distinctive endotestal thickening in the chalazal region (labelled “CET” in figures; e.g., Fig. 3S, S8b). It is either a thickening of a uniseriate endotesta (e.g., *Strelitzia*) or a multicellular endotesta (e.g., *Ravenala*). Character states are scored as follows: 0, absent; 1, present.

**41. Inner integument (tegmen):** In Musaceae and Cannaceae, the seed coat comprises both an outer and inner integument. In all other families within the order the inner integument is crushed during seed maturation and is not present in the mature seed. Character states are scored as follows: 0, absent; 1, present.

**42. Raphe canal:** The raphe in mature seeds is destroyed on some taxa, leaving a canal in the seed coat from the micropyle to the chalaza (labelled “RC” in figures; e.g., Fig. S9q). In some specimens the raphe canal terminates at (and merges with) the chalazal chamber, but can be differentiated from the chalazal chamber by being slightly smaller in diameter (e.g., Fig. 4l, S9q). Character states are scored as follows: 0, absent; 1, present.

**43. Perisperm canal:** In Marantaceae, mature seeds often have a distinct invagination of the seed coat extending from the micropyle to the chalaza (e.g., Fig. 3b, 3f-g, S5b, S5g), previously described as a perisperm channel or perisperm canal (Grootjen 1983; Andersson and Chase 2001). It is formed through the degeneration of the raphe during the early stages of seed development (Andersson 1981), but differs from the raphe canal (character 42) because of its large size and extensive modification of the testa to form the canal. Character states are scored as follows: 0, absent; 1, present.

**44. Perisperm canal shape:** The shape of the perisperm canal was originally identified by Schumann (1902), who recognized two forms, simple (not branched) and branched (Andersson 1981). Andersson further distinguished three types, simple (not branched; e.g., Fig. 3k, S6p-t), branched at the base (e.g., Fig. 3f-h, S6f-m), and branched throughout (e.g., Fig. 3i, S6a-c). We have adopted Andersson’s classification and recognize three forms. Character states are scored as follows: (–) inapplicable, no perisperm canal present; 0, simple (not distinctly branched); 1, basally branched; 2, branched throughout.

**45. Embryo length:** The embryos in most Zingiberales are elongate and extend for more than half the length of the seed, but in some taxa they are much shorter. Character states are scored as follows: 0, elongate; 1, short.

**46. Embryo shape:** The shape of the embryo ranges from being straight, L-shaped (with a sharp, nearly right-angled curve in the embryo that is less than 25% of the embryo length), J-shaped (with a smooth curve in the embryo that is ca. 50% of the embryo length). Commonly in Marantaceae, embryos curve completely around the perisperm canal and form a “U”, shape, which was also considered a J-shaped embryo. Character states are scored as follows: 0, straight; 1, L-shaped; 2, J-shaped.

**47. Embryo base:** Independent of shape, some taxa have embryos that are enlarged and bulbous, or forked at the base. Character states are scored as follows: 0, not differentiated; 1, bulbous; 2, forked.

**48. Embryo–seed coat contact:** The embryo, perisperm, and sometimes endosperm occupy the embryo cavity of Zingiberales seeds. The embryo can either be in direct contact with the seed coat or nested within endosperm/perisperm and not touching the innermost wall of the seed coat. Character states are scored as follows: 0, absent; 1, present.

**49. Well-developed perisperm:** Perisperm can be well-developed or not (character adopted from Kress 1990, 1996; Kress *et al.* 2001). Character states are scored as follows: 0, absent; 1, present.

**50. Endosperm composition:** Endosperm is either helobial or nuclear (character adopted from Kress 1990, 1996; Kress *et al.* 2001). Character states are scored as follows: 0, absent; 1, helobial; 2, nuclear.

**51. Basally proliferated endosperm:** Endosperm in some taxa is found in greater abundance surrounding the base of the embryo compared to the micropylar end. In some SRXTM images perisperm is often composed of large cells and endosperm is often darker and lacks observable cell walls. In other SRXTM images endosperm appears as small cells (perhaps nuclei) and individual perisperm cells are indistinguishable. In both cases endosperm always immediately surrounds the embryo and both the embryo and endosperm are nested within the perisperm. This character refers to the relative distribution of endosperm, and whether it is proliferated, or more abundant, toward the chalazal end of the seed as compared to the micropylar end. Character states are scored as follows: 0, absent; 1, present, weak or minimal amount; 2, present, strong or copious amount.

**LITERATURE CITED**

Andersson L. 1981. The neotropical genera of Marantaceae. Circumscription and Relationships. *Nordic Journal of Botany* 1: 218–245.

Andersson L, MW Chase MW. 2001. Phylogeny and classification of Marantaceae. *Botanical Journal of the Linnean Society* 135: 275–287.

Benedict JC, Smith SY, Collinson ME, Leong–Škorničková J, Specht CD, Fife JL, Marone F, Xiao X, Parkinson DY. 2015a. Evolutionary Significance of Seed Structure in Alpinioideae (Zingiberaceae). *Botanical Journal of the Linnean Society* 178: 441–466.

Benedict JC, Smith SY, Collinson ME, Leong–Škorničková J, Specht CD, Marone F, Xiao X, Parkinson DY. 2015b. Utility of seed morphology and anatomy and its utility in recognizing subfamilies and tribes of Zingiberaceae. *American Journal of Botany* 102(11): 1814–1841.

Bouharmont J. 1963. Evolution de l’ovule fécondé chez *Musa acuminata* Colla subsp. *burmannica* Simmonds. *La Cellule* 63: 261–279.

Corner EJH. 1976. The seeds of dicotyledons, vols. 1 and 2. Cambridge, UK: Cambridge University Press.

Grootjen CJ. 1983. Development of ovule and seed in Marantaceae. *Acta Botanica Neerlandica* 32: 69–86.

Grootjen CJ, Bouman F. 1981. Development of the ovule and seed in *Costus cuspidatus*, with special reference to the formation of the operculum. *Botanical Journal of the Linnean Society* 83: 27–39.

Hijmans RJ, Cameron SE, Parra JL, Jones PG, Jarvis A. 2005. Very high resolution interpolated climate surfaces for global land areas. *International Journal of Climatology* 25: 1965–1978.

Kapil RN, Bor J, Bouman F. 1980. Seed appendages in Angiosperms. I. Introduction. *Botanische Jahrbücher fur Systematik, Pflanzengeschichte und Pflanzengeographie* 101: 555–573.

Kress WJ. 1990. The Phylogeny and Classification of Zingiberales. *Annals of the Missouri Botanical Garden* 77(4): 698–721.

Kress WJ. 1996. Phylogeny of the Zingiberanae: Morphology and molecules. In: Wu TL, Wu QG, Chen ZY, eds. *Proceedings of the 2nd Symposium on the Family Zingiberaceae*. Guangzhou, China: Zhongshan University Press, 122–141.

Kress WJ, Prince LM, Hahn WJ, Zimmer EA. 2001. Unraveling the evolutionary radiation of the families of Zingiberales using morphological and molecular evidence. *Systematic Botany* 50: 926–944.

Liao JP, Wu QG. 1996. The significance of the seed anatomy of Chinese *Alpinia*. In: Wu TL, Wu QG, Chen ZY, eds. *Proceedings of the 2nd Symposium on the Family Zingiberaceae*. Guangzhou, China: Zhongshan University Press, 92–106.

Liao JP, Wu QG. 2000. A preliminary study of the seed anatomy of Zingiberaceae. *Botanical Journal of the Linnean Society* 134: 287–300.

Manchester SR, Kress WJ. 1993. *Ensete oregonense* sp. nov. from the Eocene of western North America and its phylogeographic significance. *American Journal of Botany* 80: 1264–1272.

McGahan MW. 1961. Studies on the seed of banana. I. Anatomy of the seed and Embryo of *Musa balbisiana.* *American Journal of Botany* 48: 230–238.

Olson DM, Dinerstein E, Wikramanayake ED, Burgess ND, Powell GVN, Underwood EC, D’Amico JA, Itoua I, Strand HE, Morrison JC *et al.,* 2001. Terrestrial ecoregions of the world: A new map of life on earth. *BioScience* 51(11): 933–938.

Ridley HN. 1909. Fruit of *Burbidgea*. *Journal of the Straits Branch of the Royal Asiatic Society* 53: 175–176.

Schumann K. 1902. Marantaceae. In: A. Engler, ed. *Das Pflanzenreich*, vol. 4. Leipzig, Germany: W. Engelmann, 1–184.

Thiers B. [continuously updated]. Index Herbariorum: A global directory of public herbaria and associated staff. New York Botanical Garden’s Virtual Herbarium. http://sweetgum.nybg.org/ih/ [accessed 01 January 2015].

Wu QG, Liao JP. 1995. Anatomy and histochemistry of the seeds of *Globba racemosa. Guihaia* 15(2): 146–153.

**Table S1** List of specimens sampled and voucher information. Herbarium abbreviations follow Index Herbariorum (Thiers continually updated).

| **Species** | **Voucher Information** | **Number of specimens visually analyzed / scanned using SRXTM** |
| --- | --- | --- |
| *Aframomum angustifolium* (Sonn.) K.Schum. | US, WJ Kress, 92-3171 | 1 seed/ 1 seed |
| *Aframomum chrysanthum* Lock | SING, GRC–173 | 10 seeds/ 1 seed |
| *Aframomum daniellii* (Hook.f.) K.Schum. | Delft University of Technology, JW van Loon | 1 seed/ 1 seed |
| *Aframomum melegueta* K.Schum. | US, J. Higgins 44 | 3 seeds/ 1 seed |
| *Alpinia aquatica* (Retz.) Roscoe | SING, GRC–22, and US, WJ Kress 05–7809 | 50+ seeds/ 3 seeds |
| *Alpinia boia* Seem. | US, WJ Kress 79–1071, and US, AC Smith 4087 | 10+ seeds/ 2 seeds |
| *Alpinia brevilabris* C.Presl | US, M Ramos 30411 | 5 seeds/ 1 seed |
| *Alpinia caerulea* (R.Br.) Benth. | SING, JLS–1660 | 20+ seeds/ 1 seed |
| *Alpinia carolinensis* Koidz. | US, DH Lorence 7907 | 10+ seeds/ 1 seed |
| *Alpinia conchigera* Griff. | SING, GRC–205 | 1 seed/ 1 seed |
| *Alpinia fax* (Thwaites) B.L.Burtt & R.M.Sm. | US, AHM Jayasuriya 1217 | 10+ seeds/ 1 fruit and 1 seed |
| *Alpinia galanga* (L.) Willd. | US, Shiu Ying Hu 6225 | 6 seeds/ 2 seeds |
| *Alpinia gigantifolia* (Elmer) R.M.Sm. | US, ADE Elmer 14603 | 4 seeds/ 1 seed |
| *Alpinia haenkei* C.Presl | US, ADE Elmer 17662 | 5 seeds/ 1 seed |
| *Alpinia japonica* (Thunb.) Miq. | NY, Muratailcitamura 639 | 1 seed/ 1 seed |
| *Alpinia javanica* Blume | SING, Umbai and Millard 1430 | 7 seeds/ 1 seed |
| *Alpinia katsumadai* Hayata | Delft University of Technology, JW van Loon | 1 seed/ 1 seed |
| *Alpinia luteocarpa* Elmer | US, Kress and Li 05–7785 | 3 seeds/ 1 seed |
| *Alpinia malaccensis* (Burm.f.) Roscoe | US, C Saldanha 14771 | 20 seeds/ 1 seed |
| *Alpinia nigra* (Gaertn.) B.L.Burtt | US, WJ Kress 00–6808 | 6 seeds/ 1 seed |
| *Alpinia oblongifolia* Hayata | US, J Wen 9436 | 2 seeds/ 1 seed |
| *Alpinia oceanica* Burkill | E, Stone and Streimann 10296 | 1 fruit/ 1 fruit |
| *Alpinia purpurata* (Vieill.) K.Schum. | E, AN Miller NGF 38482 | 1 fruit/ 1 fruit |
| *Alpinia rafflesiana* Wall. ex Baker | SING, Ridley s.n. | 8 seeds/ 1 seed |
| *Alpinia stachyodes* Hance | US, n.c., 1801 | 3 seeds/ 1 seed |
| *Alpinia zerumbet* (Pers.) B.L.Burtt & R.M.Sm. | US, Wen 9412, and US, Fosberg 38289 | 30+ seeds/ 3 seeds |
| *Amomum koenigii* J.F.Gmel. | SING, VNM–B–1443 | 4 seeds/ 1 seed |
| *Amomum lappaceum* Ridl. | SING, JLS–1667 | 50+ seeds/ 2 seeds |
| *Amomum ochreum* Ridl. | SING, JLS–1670 | 6 seeds/ 1 seed |
| *Amomum sericeum* Roxb. | SING, JLS–1273 | 20+ seeds/ 1 seed |
| *Aulotandra trigonocarpa* H.Perrier | K, M Bardot–Vaucoulon 1272 | 1 fruit/ 1 fruit |
| *Boesenbergia curtisii* (Baker) Schltr*.* | NY, Henderson 22874 | 1 seed/ 1 seed |
| *Burbidgea stenantha* Ridl. | SING, GRC–88 | 1 seed/ 1 seed |
| *Calathea inocephala* (Kuntze) T.Durand & B.D.Jacks | MICH, WA Schipp 330 | 1 seed/ 1 seed |
| *Calathea macrosepala* K.Schum. | MICH, H Kennedy *et al.,* 1461 | 2 seeds/ 1 seed |
| *Camptandra ovata* Ridl. | SING, JLS–1669 | 10+ seeds/ 1 seed |
| *Canna × generalis* L.H. Bailey & E.Z. Bailey | SING, s.n. | 1 seed/ 1 seed |
| *Canna flaccida* Salisb. | US, Kress s.n. | 3 seeds/ 1 seed |
| *Canna indica* L. | MICH, SY Smith s.n. (commercially purchased) and SING GRC–90 | 50+ seeds/ 2 seeds |
| *Canna iridiflora* Ruiz & Pav. | US, Plowman & Davis 4753 | 7 seeds/ 1 seed |
| *Canna paniculata* Ruiz & Pav. | US, WJ Kress 90-2963 | 3 seeds/ 1 seed |
| *Canna tuerckheimii* Kraenzl. | US, WJ Kress 77-720 | 10 seeds/ 1 seed |
| *Cautleya gracilis* (Sm.) Dandy | MO, K Larsen 46744 | 4 seeds/ 1 seed |
| *Cautleya spicata* (Sm.) Baker | MICH, JC Benedict s.n. (commercially purchased) | 10+ seeds/ 1 seed |
| *Chamaecostus congestiflorus* (Rich. ex L.F. Gagnep.) C.D. Specht & D.W. Stev. | NY, Prevost 1895 | 10+ seeds/ 1 seed |
| *Chamaecostus cuspidatus* (Nees & Mart.) C.D.Specht & D.W.Stev. | E, 1708-55 | 6 seeds/ 1 seed |
| *Cheilocostus tonkinensis* Gagnep. | SING, JLS-831 | 20+ seeds/ 1 seed |
| *Costus dubius* (Afzel.) K. Schum. | SING, 20050730 | 50+ seeds/ 2 seeds |
| *Costus guanaiensis* Rusby | MICH, SY Smith s.n. (commercially purchased) | 5 seeds/ 1 seed |
| *Costus hirsutus* C.Presl | MICH, CI Lei 161 | 1 fruit/ 1 fruit |
| *Costus lucanusianus* J.Braun & K.Schum. | SING, s.n. | 1 seed/ 1 seed |
| *Costus megalobractea* K.Schum. | Delft University of Technology, JW van Loon | 1 seed/ 1 seed |
| *Costus pulverulentus* C.Presl | SING, GRC-131 | 10 seeds/ 1 seed |
| *Costus spiralis* (Jacq.) Roscoe | MICH, SY Smith s.n. (commercially purchased) | 5 seeds/ 1 seed |
| *Ctenanthe lanceolata* Petersen | US, s.n. | 1 seed/ 1 seed |
| *Curcuma montana* Roxb. | SING, JLS–73474 | 1 seed/ 1 seed |
| *Curcuma pierreana* Gagnep. | SING, Ly–489 | 1 seed/ 1 seed |
| *Dimerocostus argenteus* (Ruiz & Pav.) Maas | US, WJ Kress 5915 | 2 seeds/ 1 seed |
| *Dimerocostus strobilaceus* Kuntze | US, Madison *et al.,* 5038 | 10 seeds/ 1 seed |
| *Distichochlamys citrea* M.F.Newman | SING, JLS–1615 | 1 seed/ 1 seed |
| *Donax canniformis* (G.Forst.) K.Schum | US, RS Toroes 1928 | 3 seeds/ 1 seed |
| *Elettaria cardamomum* (L.) Maton | MICH, JC Benedict s.n. (commercially purchased) | 50+ fruits/ 1 fruit |
| *Elettariopsis unifolia* (Gagnep.) M.F.Newman | MO, JF Maxwell 00–390 | 2 seeds/ 1 seed |
| *Ensete glaucum* (Roxb.) Cheesman | US, WJ Kress 83-1554; MICH, SY Smith s.n. (commercially purchased) | 8 seeds/ 2 seeds |
| *Ensete lasiocarpum* (Franch.) Cheesman | MICH, SY Smith s.n. (commercially purchased) | 5 seeds/ 1 seed |
| *Ensete perrieri* (Claverie) Cheesman | AAU, 20.809 | 2 seeds/ 1 seed |
| *Ensete superbum* (Roxb.) Cheesman | US, WJ Kress s.n. | 2 seeds/ 1 seed |
| *Ensete ventricosum* (Welw.) Cheesman | AAU, 20.806 | 5 seeds/ 1 seed |
| *Etlingera elatior* (Jack) R.M.Sm. | SING, SNG–56 | 20+ seeds/ 1 seed |
| *Etlingera linguiformis* (Roxb.) R.M.Sm. | US, WJ Kress, M Bordelon, T Htum 02–7044 | 20+ seeds/ 1 seed |
| *Etlingera yunnanensis* (T.L.Wu & S.J.Chen) R.M.Sm. | SING, JLS–1717 | 10+ seeds/ 1 seed |
| *Gagnepainia harmandii* (Baill.) K.Schum. | SING, GRC–132 | 2 seeds/ 1 seed |
| *Geocharis aurantiaca* Ridl. | SING, Corner 32777 | 9 seeds/ 1 seed |
| *Geostachys densiflora* Ridl. | SING, JLS–1662 | 10+ seeds/ 1 seed |
| *Globba aurea* Elmer | MICH, HH Bartlett 15543 | 2 seeds/ 1 seed |
| *Globba maculata* Blume | MICH, HH Bartlett 7544 | 3 seeds/ 1 seed |
| *Globba pendula* Roxb. | NYBG, Rahmat Si Toroes 3668 | 1 fruit/ 1 fruit |
| *Globba sessiliflora* Sims | SING, JLS–1957 | 7 seeds/ 1 seed |
| *Globba spathulata* Roxb. | US, WJ Kress 01–6914 | 2 seeds/ 1 seed |
| *Haumania liebrechtsiana* (De Wild. & T.Durand) J.Léonard | MO, J Leonard 15 | 1 seed/ 1 seed |
| *Hedychium coronarium* J.Koenig | MICH, A Shilom Tan 1771 | 2 seeds/ 1 seed |
| *Hedychium gardnerianum* Sheppard ex Ker Gawl. | MICH, SY Smith s.n. (commercially purchased) | 10 seeds/ 1 seed |
| *Hedychium hasseltii* Blume | US, T Wood 94–3700 | 100+ seeds/ 1 seed |
| *Hedychium muluense* R.M.Sm. | SING, JLS–54 | 20+ seeds/ 1 seeds |
| *Heliconia angusta* Vell. | US, WJ Kress 90-2954 | 1 seed/ 1 seed |
| *Heliconia bihai* (L.) L. | AAU, 20.802-1 | 4 seeds/ 1 seed |
| *Heliconia bourgaeana* Petersen | MICH, Gentle 2117 | 3 seeds/ 1 seed |
| *Heliconia brenneri* Abalo & G. Morales | US, WJ Kress 89-2857 | 4 seeds/ 1 seed |
| *Heliconia chartacea* Lane ex Barreiros | US, WJ Kress 89-2880 | 4 seeds/ 1 seed |
| *Heliconia griggsiana* L.B.Sm. | MICH, SY Smith s.n. (commercially purchased) | 5 seeds/ 1 seed |
| *Heliconia imbricata* (Kuntze) Baker | US, WJ Kress 79-1101 | 3 seeds/ 1 seed |
| *Heliconia latispatha* Benth. | MICH, SY Smith s.n. (commercially purchased) | 10 seeds/ 1 seed |
| *Heliconia longiflora* R.R.Sm. | US, Laurito 5989; MICH, SY Smith s.n. (commercially purchased) | 3 seeds/ 2 seeds |
| *Heliconia metallica* Planch. & Linden ex Hook. | US, Herrera 1728 | 3 seeds/ 1 seed |
| *Heliconia papuana* W.J.Kress | US, WJ Kress 83-1544 | 7 seeds/ 1 seed |
| *Heliconia pendula* Wawra | US, WJ Kress 91-3195 | 1 seed/ 1 seed |
| *Heliconia solomonensis* W.J.Kress | US, WJ Kress 79-1053 | 4 seeds/ 1 seed |
| *Heliconia stricta* Huber | MO, s.n. | 2 fruits/ 1 fruit |
| *Heliconia subulata* Ruiz & Pav. | US, WJ Kress 89-2898 | 2 seeds/ 1 seed |
| *Heliconia vellerigera* Poepp. | US, WJ Kress 77-78 | 5 seeds/ 1 seed |
| *Heliconia velutina* L.Andersson | US, 91-3279 | 2 seeds/ 1 seed |
| *Hellenia speciosa* (J. Koenig) Govaerts | SING, JLS-816 | 50+ seeds/ 2 seeds |
| *Hemiorchis* sp. | US, WJ Kress 01–6884 | 10 seeds/ 1 seed |
| *Hornstedtia conica* Ridl. | SING, SNG–35 | 20+ seeds/ 1 seed |
| *Hornstedtia leonurus* (J.Koenig) Retz. | SING, SNG–174 | 20+ seeds/ 1 seed |
| *Hornstedtia scottiana* (F.Muell.) K.Schum. | US, WJ Kress 80–1129 | 10 seeds/ 1 seed |
| *Hylaeanthe unilateralis* (Poepp. & Endl.) A.M.E.Jonker & Jonker | MO, A Rodriguez 1523 | 1 seed/ 1 seed |
| *Hypselodelphys scandens* Louis & Mullend. | MO, Jean Louis 7082 | 1 seed/ 1 seed |
| *Indianthus virgatus* (Roxb.) Suksathan & Borchs. | SING, 19990772 | 6 seeds/ 1 seed |
| *Ischnosiphon inflatus* L.Andersson | MO, Mori & J.Kallunki 1807 | 1 seed/ 1 seed |
| *Kaempferia pulchra* Ridl. | K, Rabil 296 | 1 seed/ 1 seed |
| *Maranta arundinacea* L. | MICH, JL Luteyn & M Lebron-Luteyn s.n. | 2 seeds/ 1 seed |
| *Marantochloa leucantha* (K.Schum.) Milne-Redh. | SING, 20011702 | 12 seeds/ 1 seed |
| *Megaphrynium macrostachyum* (K.Schum.) Milne-Redh. | MO, Breteler 6336 | 1 fruit and 1 seed/ 1 seed |
| *Monocostus uniflorus* (Poepp. ex Petersen) Maas | US, Jose Schunke V 9783 | 1 seed/ 1 seed |
| *Monolophus sikkimensis* (King ex Baker) Veldkamp & Mood | SING, Wallich s.n. | 5 seeds/ 1 seed |
| *Monotagma laxum* (Poepp. & Endl.) K.Schum. | MO, R Vasques, R Rojas & A Pena 22590 | 2 seeds/ 1 seed |
| *Musa × paradisiaca* L. | AAU, 3931 | 6 seeds/ 1 seed |
| *Musa acuminata* Colla | MICH, SY Smith s.n. (commercially purchased) | 10 seeds/ 1 seed |
| *Musa acuminata* subsp. *malaccensis* (Ridl.) N.W.Simmonds | MICH, JN Milsum s.n. | 3 seeds/ 1 seed |
| *Musa balbisiana* Colla | AAU, s.n. | 6 seeds/ 1 seed |
| *Musa coccinea* Andrews | US, WJ Kress 94-3602 | 1 fruit and 20+ seeds/ 1 fruit and 1 seed |
| *Musa mannii* H.Wendl. ex Baker | AAU, 11727 | 1 seed/ 1 seed |
| *Musa peekelii* Lauterb. | AAU, s.n. | 1 fruit and 20+ seeds/ 1 seed |
| *Musa sakaiana* Meekiong, Ipor & Tawan | MICH, SY Smith s.n. (commercially purchased) | 5 seeds/ 2 seeds |
| *Musa schizocarpa* N.W.Simmonds | K, RJ Johns 8140 | 5 seeds/ 1 seed |
| *Musa sikkimensis* Kurz | MICH, JC Benedict s.n. (commercially purchased) | 6 seeds/ 1 seed |
| *Musa textilis* Née | MICH, SY Smith s.n. (commercially purchased) | 5 seeds/ 1 seed |
| *Musa velutina* H.Wendl. & Drude | MICH, SY Smith s.n. (commercially purchased) | 6 seeds/ 1 seed |
| *Newmania* sp. | SING, JLS–1646 | 5 seeds/ 1 seed |
| *Orchidantha maxillarioides* (Ridl.) K.Schum. | SING, JLS-639 | 2 seeds/ 1 seed |
| *Orchidantha sabahensis* A.L.Lamb & L.B.Pedersen | SING, JLS s.n. | 2 seeds/ 1 seed |
| *Orchidantha stercorea* H.Ð.Trần & Škorničk. | SING, JLS-550 | 3 seeds/ 1 seed |
| *Orchidantha vietnamica* K.Larsen | SING, JLS-1115 | 1 seed/ 1 seed |
| *Phenakospermum guyannense* (A.Rich.) Endl. ex Miq. | MICH, BW Nelson 1153 | 1 seed/ 1 seed |
| *Phrynium imbricatum* Roxb. | SING, JLS-1405 | 2 seeds/ 1 seed |
| *Phrynium interruptum* (K.Schum.) Suksathan & Borchs. | MO, ADE Elmer 11068 | 1 fruit/ 1 seed |
| *Phrynium* sp. | SING, JLS s.n. | 14 seeds/ 1 seed |
| *Plagiostachys escritorii* Elmer | NY, Elmer 16216 | 1 seed/ 1 seed |
| *Plagiostachys philippinensis* Ridl. | NY, Ramos and Edaño 75626 | 1 seed/ 1 seed |
| *Pleiostachya pittieri* Rowlee ex Standl. | MO, S Mori and J Kallunki 3360 | 2 seeds/ 1 seed |
| *Pleuranthodium* sp. | US, TG Hartley 10989 | 3 seeds/ 1 seed |
| *Ravenala madagascariensis* Sonn. | SING, JLS s.n. | 5 seeds/ 1 seed |
| *Renealmia lucida* Maas | SING, JLS–1019 | 1 seed/ 1 seed |
| *Renealmia occidentalis* (Sw.) Sweet | MICH, J Vera Santos 2513 | 2 fruits with 10+ seeds/ 1 seed |
| *Riedelia corallina* (K.Schum.) Valeton | NY, Annable 3639 | 1 seed/ 1 seed |
| *Riedelia* sp. | SING, JLS–428 | 10+ seeds/ 1 seed |
| *Roscoea alpina* Royle | AAU, 1013 | 50+ seeds/ 1 seed |
| *Sarcophrynium brachystachyum* (Benth.) K.Schum. | SING, 20002554 | 8 seeds/ 1 seed |
| *Sarcophrynium prionogonium* (K.Schum.) K.Schum. | NY, Reitsma 2231 | 1 fruit with 3 seeds/ 1 fruit with 3 seeds |
| *Siamanthus siliquosus* K.Larsen & J.Mood | US, WJ Kress 99-6358 | 2 seeds/ 1 seed |
| *Siliquamomum tonkinense* Baill. | SING, VNM-B-1469 | 10+ seeds/ 1 seed |
| *Siphonochilus aethiopicus* (Schweinf.) B.L.Burtt | MO, P Kuchar 22948 | 5 seeds/ 1 seed |
| *Siphonochilus kirkii* (Hook.f.) B.L.Burtt | MO, AB Katende K1880 | 3 seeds/ 1 seed |
| *Stachyphrynium sumatranum* (Miq.) K.Schum | SING, Forman and Blewett 1200 | 2 seeds/ 1 seed |
| *Strelitzia nicolai* Regel & K.Koch | US, s.n. | 1 seed/ 1 seed |
| *Strelitzia reginae* Banks | MICH, SY Smith s.n. (commercially purchased) | 5 seeds/ 1 seed |
| *Stromanthe stromanthoides* (J.F. Macbr.) L. Andersson | MICH, CM Belshaw 3568 | 1 seed/ 1 seed |
| *Tapeinochilus* sp. | NY, Clemens 1965 | 1 fruit with 50+ seeds/ 1 fruit with 50+ seeds |
| *Thalia geniculata* L. | SING, GRC-61 | 14 seeds/ 1 seed |
| *Thaumatococcus daniellii* (Benn.) Benth. | SING, JLS s.n. | 6 seeds/ 1 seed |
| *Trachyphrynium braunianum* (K.Schum.) Baker | MO, JB Hall 42304 | 2 seeds/ 2 seeds |
| *Vanoverberghia sepulchrei* Merr. | NY, Ramos and Edaño 45045 | 1 fruit with 50+ seeds/ 1 fruit with 50+ seeds |
| *Zingiber larsenii* Theilade | SING, JLS–1270 | 18 seeds/ 1 seed |
| *Zingiber officinale* Roscoe | Delft University of Technology, JW van Loon | 1 seed/ 1 seed |
| *Zingiber spectabile* Griff. | Delft University of Technology, JW van Loon | 1 seed/ 1 seed |
| *Zingiber thorelii* Gagnep. | SING, JLS–1271 | 15 seeds/ 1 seed |

**Table S2** Distribution of seed characters within Zingiberales. Character numbers are as follows (see SUPPORTING INFORMATION for definitions): (1) Sclerenchymatous endocarp (2) Natural seed/endocarp color; (3) Seed surface; (4) Trichomes on seed coat or aril; (5) Aril; (6) General seed shape; (7) Seeds contorted from arrangement in fruit; (8) Seed length; (9) Seed body taper at micropyle; (10) Seed body taper at chalaza; (11) Externally visible raphe; (12) External chalazal indentation; (13) Stomata in seed coat; (14) Micropylar region shape; (15) Operculum; (16) Operculum layering; (17) Operculum position; (18) Micropylar collar; (19) Micropylar collar layering; (20) Thickened micropylar collar; (21) Recurved micropylar collar; (22) Hilar rim; (23) Hilar rim layering; (24) Micropylar mesotesta proliferation; (25) Massive chalazal testal proliferations; (26) Columnar chalazal testal proliferations; (27) Chalazal chamber; (28) Chalazal chamber column; (29) Chalazal mucro; (30) Mechanical layer thickness; (31) Mechanical layer; (32) Exotesta cell type; (33) Uniform exotesta; (34) Multiseriate exotesta; (35) Portion of exotesta palisade at chalaza; (36) Mesotesta layer number; (37) Endotesta cell shape and thickness; (38) Endotestal gap location; (39) Chalazal pigment group; (40) Chalazal endotestal thickening; (41) Inner integument; (42) Raphe canal; (43) Perisperm canal; (44) Perisperm canal shape; (45) Embryo length; (46) Embryo shape; (47) Embryo base; (48) Embryo–seed coat contact; (49) Well-developed perisperm; (50) Endosperm composition; (51) Basally proliferated endosperm. (–) character not applicable, (?) character state unknown. CI = consistency index; HI = homoplasy index; RI = retention index.

| Character number | 1 | 2 | 3 | 4 | 5 | 6 | 7 | 8 | 9 | 10 | 11 | 12 | 13 | 14 | 15 | 16 | 17 | 18 | 19 | 20 | 21 | 22 | 23 | 24 | 25 |
| --- | --- | --- | --- | --- | --- | --- | --- | --- | --- | --- | --- | --- | --- | --- | --- | --- | --- | --- | --- | --- | --- | --- | --- | --- | --- |
| CI | 1 | 0.07 | 0.38 | 0.17 | 0.23 | 0.08 | 0.04 | 0.04 | 0.03 | 0.03 | 0.13 | 0.08 | 1 | 0.11 | 0.25 | 0.05 | 0.33 | 0.2 | 0.04 | 0.06 | 0.06 | 0.09 | 0.17 | 0.09 | 0.06 |
| RI | 1 | 0.46 | 0.56 | 0.64 | 0.63 | 0.38 | 0.48 | 0.26 | 0.59 | 0.49 | 0.53 | 0.67 | 1 | 0.71 | 0.67 | 0.54 | 0.9 | 0.87 | 0.59 | 0.24 | 0.53 | 0.81 | 0 | 0.38 | 0.57 |
| HI | 0 | 0.93 | 0.62 | 0.83 | 0.77 | 0.92 | 0.96 | 0.96 | 0.97 | 0.97 | 0.87 | 0.92 | 0 | 0.89 | 0.75 | 0.95 | 0.67 | 0.8 | 0.96 | 0.94 | 0.94 | 0.91 | 0.83 | 0.91 | 0.94 |
| **Cannaceae** |  |  |  |  |  |  |  |  |  |  |  |  |  |  |  |  |  |  |  |  |  |  |  |  |  |
| *Canna flaccida* | 0 | 2 | 0 | 0 | 0 | 1 | 0 | 0 | 0 | 0 | 0 | 0 | 1 | 0 | 1 | 1 | 0 | 1 | 1 | 0 | 1 | 0 | - | 0 | 0 |
| *Canna indica* | 0 | 2 | 0 | 0 | 0 | 1 | 0 | 0 | 0 | 0 | 0 | 0 | 1 | 0 | 1 | 1 | 0 | 1 | 1 | 0 | 1 | 0 | - | 0 | 0 |
| *Canna iridiflora* | 0 | 2 | 0 | 0 | 0 | 0 | 0 | 0 | 1 | 1 | 0 | 0 | 1 | 0 | 1 | 1 | 0 | 1 | 1 | 0 | 1 | 0 | - | 0 | 0 |
| *Canna paniculata* | 0 | 2 | 0 | 0 | 0 | 1 | 0 | 0 | 0 | 1 | 0 | 0 | 1 | 0 | 1 | 1 | 0 | 1 | 1 | 0 | 1 | 0 | - | 0 | 0 |
| *Canna tuerckheimii* | 0 | 2 | 0 | 0 | 0 | 0 | 0 | 0 | 1 | 1 | 0 | 0 | 1 | 0 | 1 | 1 | 0 | 1 | 1 | 0 | 1 | 0 | - | 0 | 0 |
| *Canna x generalis* | 0 | 2 | 0 | 0 | 0 | 0 | 0 | 0 | 0 | 0 | 0 | 0 | 1 | 0 | 1 | 1 | 0 | 1 | 1 | 0 | 1 | 0 | - | 0 | 0 |
| **Costaceae** |  |  |  |  |  |  |  |  |  |  |  |  |  |  |  |  |  |  |  |  |  |  |  |  |  |
| *Chamaecostus congestiflorus* | 0 | 2 | 2 | 0 | 1 | 3 | 1 | 0 | 0 | 1 | 1 | 1 | 0 | 0 | 1 | 1 | 0 | 1 | 0 | 0 | 1 | 0 | - | 0 | 0 |
| *Chamaecostus cuspidatus* | 0 | 2 | 2 | 0 | 1 | 0 | 1 | 0 | 1 | 1 | 1 | 1 | 0 | 1 | 1 | 0 | 0 | 1 | 1 | 0 | 1 | 0 | - | 0 | 0 |
| *Cheilocostus tonkinensis* | 0 | 2 | 3 | 0 | 1 | 3 | 1 | 0 | 0 | 0 | 1 | 1 | 0 | 1 | 1 | 0 | 0 | 1 | 0 | 0 | 1 | 0 | - | 0 | 0 |
| *Costus dubius* | 0 | 2 | 3 | 0 | 2 | 0 | ? | 0 | 0 | 0 | 1 | 1 | 0 | 1 | 1 | 1 | 0 | 1 | 0 | 0 | 1 | 0 | - | 0 | 0 |
| *Costus guanaiensis* | 0 | 2 | 1 | 0 | 1 | 3 | 1 | 0 | 1 | 0 | 1 | 1 | 0 | 1 | 1 | 1 | 0 | 1 | 1 | 0 | 1 | 0 | - | 0 | 0 |
| *Costus hirsutus* | 0 | 2 | 0 | 0 | 1 | 1 | 1 | 0 | 1 | 0 | 1 | 1 | 0 | 0 | 1 | 1 | 0 | 1 | 0 | 0 | 1 | 0 | - | 0 | 0 |
| *Costus lucanusianus* | 0 | 2 | 1 | 0 | 2 | 0 | 0 | 0 | 0 | 0 | 1 | 1 | 0 | 1 | 1 | 1 | 0 | 1 | 0 | 0 | 1 | 0 | - | 0 | 0 |
| *Costus megalobractea* | 0 | ? | ? | 0 | 2 | 0 | 0 | 0 | 1 | 0 | ? | ? | 0 | 1 | 1 | 0 | 0 | 1 | 0 | 0 | 1 | 0 | - | 0 | 0 |
| *Costus pulverulentus* | 0 | 2 | 2&3 | 0 | 1 | 0 | 1 | 0 | 1 | 1 | 1 | 1 | 0 | 0 | 1 | 1 | 0 | 1 | 1 | 0 | 1 | 0 | - | 0 | 0 |
| *Costus spiralis* | 0 | 2 | 0 | 0 | 1 | 3 | 1 | 0 | 1 | 0 | 1 | 1 | 0 | 1 | 1 | 1 | 0 | 1 | 1 | 0 | 1 | 0 | - | 0 | 0 |
| *Dimerocostus argenteus* | 0 | 2 | 0 | 0 | 1 | 3 | 1 | 1 | 0 | 0 | 0 | 1 | 0 | 1 | 1 | 0 | 0 | 1 | 1 | 0 | 1 | 0 | - | 0 | 0 |
| *Dimerocostus strobilaceus* | 0 | 2 | 2 | 0 | ? | 1 | 1 | 0 | 0 | 1 | 0 | 1 | 0 | 0 | 1 | 1 | 0 | 1 | 1 | 0 | 1 | 0 | - | 0 | 0 |
| *Hellenia speciosa* | 0 | 2 | 0 | 0 | 1 | 3 | 1 | 0 | 0 | 0 | 1 | 1 | 0 | 0 | 1 | 0 | 0 | 1 | 0 | 0 | 1 | 0 | - | 0 | 0 |
| *Monocostus uniflorus* | 0 | 1 | 1&2 | 0 | 2 | 3 | 1 | 0 | 0 | 1 | 1 | 1 | 0 | 1 | 1 | 0 | 0 | 1 | 1 | 0 | 1 | 0 | - | 0 | 0 |
| *Tapeinochilus* sp. | 0 | 2 | 1 | 0 | 1 | 3 | 1 | 0 | 0 | 0 | 0 | 1 | 0 | 1 | 1 | 1 | 0 | 1 | 1 | 0 | 1 | 0 | - | 0 | 0 |
| **Heliconiaceae** |  |  |  |  |  |  |  |  |  |  |  |  |  |  |  |  |  |  |  |  |  |  |  |  |  |
| *Heliconia angusta* | 1 | 1 | 4 | 0 | 0 | 0 | 0 | 0 | 1 | 0 | 0 | 0 | 0 | 0 | 1 | 1 | 1 | 0 | - | - | - | 0 | - | 0 | 0 |
| *Heliconia bihai* | 1 | 1 | 4 | 0 | 0 | 0 | 0 | 1 | 1 | 1 | 0 | 0 | 0 | 0 | 1 | 1 | 1 | 0 | - | - | - | 0 | - | 0 | 0 |
| *Heliconia bourgaeana* | 1 | 1 | 4 | 0 | 0 | 0 | 0 | 1 | 1 | 0 | 0 | 0 | 0 | 0 | 1 | 1 | 1 | 0 | - | - | - | 0 | - | 0 | 0 |
| *Heliconia brenneri* | 1 | 0 | 4 | 0 | 0 | 0 | 0 | 0 | 1 | 1 | 0 | 0 | 0 | 0 | 1 | 1 | 1 | 0 | - | - | - | 0 | - | 0 | 0 |
| *Heliconia chartacea* | 1 | 0 | 4 | 0 | 0 | 1 | 0 | 0 | 1 | 1 | 0 | 0 | 0 | 0 | 1 | 1 | 1 | 0 | - | - | - | 0 | - | 0 | 0 |
| *Heliconia griggsiana* | 1 | 0 | 4 | 0 | 0 | 4 | 0 | 0 | 1 | 0 | 0 | 0 | 0 | 0 | 1 | 1 | 1 | 0 | - | - | - | 0 | - | 0 | 0 |
| *Heliconia imbricata* | 1 | 0 | 4 | 0 | 0 | 1 | 0 | 0 | 1 | 1 | 0 | 0 | 0 | 0 | 1 | 1 | 1 | 0 | - | - | - | 0 | - | 0 | 0 |
| *Heliconia latispatha* | 1 | 1 | 4 | 0 | 0 | 0 | 0 | 0 | 1 | 0 | 0 | 0 | 0 | 0 | 1 | 1 | 1 | 0 | - | - | - | 0 | - | 0 | 0 |
| *Heliconia longiflora* | 1 | 1 | 4 | 0 | 0 | 0 | 0 | 0 | 0 | 1 | 0 | 0 | 0 | 0 | 1 | 1 | 1 | 0 | - | - | - | 0 | - | 0 | 0 |
| *Heliconia metallica* | 1 | 0 | 4 | 0 | 0 | 1 | 0 | 0 | 1 | 0 | 0 | 0 | 0 | 0 | 1 | 1 | 1 | 0 | - | - | - | 0 | - | 0 | 0 |
| *Heliconia papuana* | 1 | 1 | 4 | 0 | 0 | 1 | 0 | 1 | 1 | 1 | 0 | 0 | 0 | 0 | 1 | 1 | 1 | 0 | - | - | - | 0 | - | 0 | 0 |
| *Heliconia pendula* | 1 | 1 | 4 | 0 | 0 | 0 | 0 | 0 | 1 | 1 | 0 | 0 | 0 | 0 | 1 | 1 | 1 | 0 | - | - | - | 0 | - | 0 | 0 |
| *Heliconia solomonensis* | 1 | 0 | 4 | 0 | 0 | 1 | 0 | 1 | 1 | 1 | 0 | 0 | 0 | 0 | 1 | 1 | 1 | 0 | - | - | - | 0 | - | 0 | 0 |
| *Heliconia stricta* | 1 | 1 | 4 | 0 | 0 | 0 | 0 | 1 | 1 | 0 | 0 | 0 | 0 | 0 | 1 | 1 | 1 | 0 | - | - | - | 0 | - | 0 | 0 |
| *Heliconia subulata* | 1 | 0 | 4 | 0 | 0 | 0 | 0 | 0 | 1 | 1 | 0 | 0 | 0 | 0 | 1 | 1 | 1 | 0 | - | - | - | 0 | - | 0 | 0 |
| *Heliconia vellerigera* | 1 | 0 | 4 | 0 | 0 | 0 | 0 | 0 | 1 | 1 | 0 | 0 | 0 | 0 | 1 | 1 | 1 | 0 | - | - | - | 0 | - | 0 | 0 |
| *Heliconia velutina* | 1 | 1 | 4 | 0 | 0 | 0 | 0 | 0 | 1 | 0 | 0 | 0 | 0 | 0 | 1 | 1 | 1 | 0 | - | - | - | 0 | - | 0 | 0 |
| **Lowiaceae** |  |  |  |  |  |  |  |  |  |  |  |  |  |  |  |  |  |  |  |  |  |  |  |  |  |
| *Orchidantha maxillarioides* | 0 | 1 | 1 | 1 | 2 | 1 | 0 | 0 | 1 | 0 | 0 | 0 | 0 | 0 | 1 | 0 | 0 | 0 | - | - | - | 1 | 1 | 0 | 0 |
| *Orchidantha sabahensis* | 0 | 1 | 1&2 | 1 | 2 | 1 | 0 | 0 | 1 | 0 | 0 | 0 | 0 | 0 | 1 | 0 | 0 | 0 | - | - | - | 1 | 1 | 0 | 0 |
| *Orchidantha stercorea* | 0 | 0 | 0 | 1 | 2 | 1 | 0 | 0 | 0 | 0 | 0 | 0 | 0 | 0 | 1 | 0 | 0 | 0 | - | - | - | 1 | 1 | 0 | ? |
| *Orchidantha vietnamica* | 0 | 1 | 1&2 | 1 | 2 | 1 | 0 | 0 | 1 | 0 | ? | ? | 0 | 0 | 1 | 0 | 0 | 0 | - | - | - | 1 | 1 | 0 | 0 |
| **Marantaceae** |  |  |  |  |  |  |  |  |  |  |  |  |  |  |  |  |  |  |  |  |  |  |  |  |  |
| *Calathea inocephala* | 0 | 2 | 2 | 0 | 1 | 0 | 0 | 1 | 0 | 0 | 0 | 0 | 0 | 0 | 1 | 0 | 0 | 1 | 0 | 0 | 1 | 0 | - | 0 | 0 |
| *Calathea macrosepala* | 0 | 2 | 3 | 0 | 1 | 0 | 0 | 0 | 1 | 0 | 0 | 0 | 0 | 0 | 1 | 0 | 0 | 1 | 0 | 0 | 1 | 0 | - | 0 | 0 |
| *Ctenanthe lanceolata* | 0 | 1 | 3 | 0 | 1 | 0 | 0 | 0 | 0 | 1 | 0 | 0 | 0 | 0 | 1 | 0 | 0 | 1 | 0 | 0 | 1 | 0 | - | 0 | 0 |
| *Donax canniformis* | 0 | 1 | 3 | 0 | 0 | 0 | 0 | 0 | 0 | 0 | 0 | 0 | 0 | 0 | 1 | 0 | 1 | 1 | 0 | 0 | 1 | 0 | - | 0 | 0 |
| *Haumania liebrechtsiana* | 0 | 1 | 0 | 0 | 1 | 1 | 0 | 0 | 0 | 0 | 0 | 0 | 0 | 0 | 1 | 0 | 0 | 1 | 0 | 0 | 1 | 0 | - | 0 | 0 |
| *Hylaeanthe unilateralis* | 0 | 1 | 3 | 0 | 1 | 1 | 0 | 0 | 1 | 0 | 0 | 0 | 0 | 0 | 1 | 1 | 0 | 1 | 0 | 0 | 1 | 0 | - | 0 | 0 |
| *Hypselodelphys scandens* | 0 | 2 | 0 | 0 | 1 | 1 | 0 | 0 | 0 | 1 | 0 | 0 | 0 | 0 | 1 | 0 | 0 | 1 | 0 | 0 | 1 | 0 | - | 0 | 0 |
| *Indianthus virgatus* | 0 | 1 | 3 | 0 | 1 | 0 | 0 | 0 | 1 | 0 | 0 | 0 | 0 | 0 | 1 | 0 | 0 | 1 | 0 | 0 | 1 | 0 | - | 0 | 0 |
| *Ischnosiphon inflatus* | 0 | 2 | 0 | 0 | 1 | 0 | 0 | 1 | 0 | 1 | 0 | 0 | 0 | 0 | 1 | 0 | 0 | 1 | 0 | 0 | 1 | 0 | - | 0 | 0 |
| *Maranta arundinacea* | 0 | 2 | 3 | 0 | 1 | 1 | 0 | 0 | 0 | 0 | 0 | 0 | 0 | 0 | 1 | 0 | 0 | 1 | 0 | 0 | 1 | 0 | - | 0 | 0 |
| *Marantochloa leucantha* | 0 | 2 | 3 | 0 | 1 | 1 | 0 | 0 | 0 | 0 | 0 | 0 | 0 | 0 | 1 | 0 | 1 | 1 | 0 | 0 | 1 | 0 | - | 0 | 0 |
| *Megaphrynium macrostachyum* | 0 | 1 | 3 | 0 | 1 | 1 | 0 | 0 | 0 | 1 | 0 | 0 | 0 | 0 | 1 | 0 | 0 | 1 | 0 | 0 | 1 | 0 | - | 0 | 0 |
| *Monotagma laxum* | 0 | 2 | 4 | 0 | 1 | 0 | 0 | 1 | 0 | 1 | 0 | 0 | 0 | 0 | 1 | 0 | 0 | 1 | 0 | 0 | 1 | 0 | - | 0 | 0 |
| *Phrynium* sp. | 0 | 1 | 4 | 0 | 1 | 0 | 0 | 1 | 0 | 0 | 0 | 0 | 0 | 0 | 1 | 0 | 1 | 1 | 0 | 0 | 1 | 0 | - | 0 | 0 |
| *Phrynium imbricatum* | 0 | 2 | 4 | 0 | 1 | 0 | 0 | 0 | 0 | 0 | 0 | 0 | 0 | 0 | 1 | 1 | 1 | 1 | 0 | 0 | 1 | 0 | - | 0 | 0 |
| *Phrynium interruptum* | 0 | 2 | 4 | 0 | 1 | 1 | 0 | 0 | 0 | 1 | 0 | 0 | 0 | 0 | 1 | 0 | 1 | 1 | 0 | 0 | 1 | 0 | - | 0 | 0 |
| *Pleiostachya pittieri* | 0 | ? | 3 | 0 | 1 | 0 | 0 | 1 | 0 | 1 | 0 | 0 | 0 | 0 | 1 | 0 | 0 | 1 | 0 | 0 | 1 | 0 | - | 0 | 0 |
| *Sarcophrynium brachystachyum* | 0 | 2 | 3 | 0 | 1 | 4 | 0 | 0 | 0 | 0 | 0 | 0 | 0 | 0 | 1 | 0 | 0 | 1 | 0 | 0 | 1 | 0 | - | 0 | 0 |
| *Stachyphrynium sumatranum* | 0 | ? | 3 | 0 | 1 | 1 | 0 | 0 | 0 | 1 | 0 | 0 | 0 | 0 | 1 | 0 | 0 | 1 | 0 | 0 | 1 | 0 | - | 0 | 0 |
| *Stromanthe stromanthoides* | 0 | 1 | 3 | 0 | 1 | 0 | 0 | 0 | 0 | 0 | 0 | 0 | 0 | 0 | 1 | 0 | 0 | 1 | 1 | 0 | 1 | 0 | - | 0 | 0 |
| *Thalia geniculata* | 0 | 2 | 0 | 0 | 1 | 0 | 0 | 0 | 0 | 1 | 0 | 0 | 0 | 0 | 1 | 0 | 0 | 1 | 0 | 0 | 1 | 0 | - | 0 | 0 |
| *Thaumatococcus daniellii* | 0 | 2 | 2 | 0 | 1 | 1 | 0 | 0 | 0 | 1 | 0 | 0 | 0 | 0 | 1 | 0 | 0 | 1 | 0 | 0 | 1 | 0 | - | 0 | 0 |
| *Trachyphrynium braunianum* | 0 | 2 | 0 | 0 | 1 | 1 | 0 | 0 | 0 | 1 | 0 | 0 | 0 | 0 | 1 | 0 | 0 | 1 | 0 | 0 | 1 | 0 | - | 0 | 0 |
| **Musaceae** |  |  |  |  |  |  |  |  |  |  |  |  |  |  |  |  |  |  |  |  |  |  |  |  |  |
| *Musa acuminata* | 0 | 2 | 2 | 0 | 3 | 1 | 0 | 0 | 1 | 0 | 0 | 0 | 0 | 2 | 1 | 1 | 0 | 1 | 1 | 1 | 0 | 1 | 1 | 0 | 0 |
| *Musa acuminata subsp. malaccensis* | 0 | 2 | 2 | 0 | 3 | 2 | 1 | 0 | 1 | 0 | 0 | 1 | 0 | 2 | 1 | 1 | 0 | 1 | 1 | 0 | 0 | 1 | 1 | 0 | 0 |
| *Musa balbisiana* | 0 | 2 | 2 | 0 | 3 | 1 | 0 | 0 | 1 | 0 | 0 | 1 | 0 | 2 | 1 | 1 | 0 | 1 | 1 | 0 | 0 | 1 | 1 | 0 | 0 |
| *Musa coccinea* | 0 | 1 | 3 | ? | 3 | 2 | 0 | 0 | 1 | 0 | 0 | 1 | 0 | 2 | 1 | 1 | 0 | 1 | 1 | 0 | 0 | 1 | 1 | 0 | 0 |
| *Musa mannii* | 0 | 1 | 3 | 0 | 3 | 2 | 0 | 0 | 1 | 0 | 0 | 1 | 0 | 2 | 1 | 1 | 0 | 1 | 1 | 1 | 0 | 1 | 1 | 0 | 0 |
| *Musa peekelii* | 0 | 2 | 3 | 0 | 3 | 2 | 1 | 0 | 0 | 0 | 1 | 0 | 0 | 2 | 1 | 1 | 0 | 1 | 1 | 0 | 0 | 0 | - | 0 | 0 |
| *Musa sakaiana* | 0 | 2 | 3 | 0 | 3 | 1 | 1 | 0 | 0 | 0 | 0 | 1 | 0 | 2 | 1 | 1 | 0 | 1 | 1 | 0 | 0 | 0 | - | 0 | 0 |
| *Musa schizocarpa* | 0 | 2 | 3 | 0 | 3 | 2 | 0 | 0 | 1 | 0 | 0 | 1 | 0 | 2 | 1 | 1 | 0 | 1 | 1 | 0 | 0 | 1 | 1 | 0 | 0 |
| *Musa sikkimensis* | 0 | 1 | 3 | 0 | 3 | 2 | 0 | 0 | 1 | 0 | 0 | 0 | 0 | 2 | 1 | 1 | 0 | 1 | 1 | 1 | 0 | 1 | 1 | 0 | 0 |
| *Musa textilis* | 0 | 2 | 2 | 0 | 3 | 1 | 0 | 0 | 0 | 1 | 0 | 1 | 0 | 2 | 1 | 1 | 0 | 1 | 1 | 0 | 0 | 0 | - | 0 | 0 |
| *Musa velutina* | 0 | 2 | 2 | 0 | 3 | 2 | 0 | 0 | 0 | 1 | 1 | 0 | 0 | 2 | 1 | 1 | 0 | 1 | 1 | 0 | 0 | 1 | 1 | 0 | 0 |
| *Musa × paradisiaca* | 0 | 2 | 3 | 0 | 3 | 2 | 0 | 0 | 0 | 0 | 0 | 1 | 0 | 2 | 1 | 1 | 0 | 1 | 1 | 1 | 0 | 1 | 1 | 0 | 0 |
| *Ensete glaucum* | 0 | 2 | 0 | 0 | 3 | 0 | 0 | 0 | 0 | 1 | 0 | 1 | 0 | 2 | 1 | 1 | 0 | 1 | 1 | 1 | 0 | 1 | 1 | 0 | 0 |
| *Ensete lasiocarpum* | 0 | 2 | 0 | 0 | 3 | 1 | 1 | 0 | 0 | 0 | 0 | 1 | 0 | 2 | 1 | 1 | 0 | 1 | 1 | 0 | 0 | 1 | 1 | 0 | 0 |
| *Ensete perrieri* | 0 | 2 | 3 | 0 | 3 | 1 | 0 | 0 | 1 | 0 | 0 | 1 | 0 | 2 | 1 | 1 | 0 | 1 | 1 | 0 | 0 | 1 | 1 | 0 | 0 |
| *Ensete superbum* | 0 | 1 | 0 | 0 | 3 | 1 | 0 | 0 | 1 | 0 | 0 | 1 | 0 | 2 | 1 | 1 | 0 | 1 | 1 | 1 | 0 | 1 | 1 | 1 | 0 |
| *Ensete ventricosum* | 0 | 2 | 0 | 0 | 3 | 1 | 0 | 0 | 0 | 0 | 0 | 1 | 0 | 2 | 1 | 1 | 0 | 1 | 1 | 1 | 0 | 1 | 1 | 0 | 0 |
| **Strelitziaceae** |  |  |  |  |  |  |  |  |  |  |  |  |  |  |  |  |  |  |  |  |  |  |  |  |  |
| *Phenakospermum guyannense* | 0 | 2 | 3 | 0 | 2 | 1 | 0 | 0 | 1 | 0 | 0 | 0 | 0 | 0 | 0 | - | - | 0 | - | - | - | 0 | - | 0 | 1 |
| *Ravenala madagascariensis* | 0 | 2 | 3 | 0 | 2 | 2 | 0 | 0 | 1 | 0 | 0 | 0 | 0 | 0 | 0 | - | - | 0 | - | - | - | 0 | - | 0 | 0 |
| *Strelitzia nicolai* | 0 | 2 | 1 | 0 | 2 | 1 | 0 | 0 | 1 | 1 | 0 | 0 | 0 | 0 | 0 | - | - | 0 | - | - | - | 0 | - | 1 | 0 |
| *Strelitzia reginae* | 0 | 2 | 1 | 0 | 2 | 0 | 0 | 0 | 1 | 0 | 0 | 0 | 0 | 0 | 0 | - | - | 0 | - | - | - | 0 | - | 1 | 0 |
| **Zingiberaceae** |  |  |  |  |  |  |  |  |  |  |  |  |  |  |  |  |  |  |  |  |  |  |  |  |  |
| *Aframomum angustifolium* | 0 | 2 | 1 | 0 | 3 | 0 | 0 | 1 | 1 | 1 | 0 | 0 | 0 | 2 | 1 | 1 | 0 | 1 | 1 | 0 | 1 | 1 | 1 | 1 | 1 |
| *Aframomum chrysanthum* | 0 | 2 | 1 | 0 | 3 | 0 | 0 | 0 | 1 | 1 | 0 | 0 | 0 | 2 | 1 | 1 | 0 | 1 | 1 | 0 | 1 | 1 | 1 | 1 | 1 |
| *Aframomum daniellii* | 0 | 2 | 1 | 0 | 3 | 0 | 0 | 0 | 1 | 1 | 0 | 0 | 0 | 2 | 1 | 1 | 0 | 1 | 1 | 0 | 1 | 1 | 0 | 1 | 1 |
| *Aframomum melegueta* | 0 | 2 | 1 | 0 | 3 | 0 | 0 | 0 | 1 | 1 | 0 | 0 | 0 | 2 | 1 | 1 | 0 | 1 | 1 | 0 | 1 | 1 | 0 | 1 | 1 |
| *Alpinia aquatica* | 0 | 2 | 2 | 0 | 3 | 2 | 0 | 0 | 0 | 0 | 1 | 1 | 0 | 0 | 1 | 1 | 0 | 1 | 1 | 1 | 1 | 0 | - | 0 | 0 |
| *Alpinia boia* | 0 | 1 | 1 | 0 | 3 | 1 | 0 | 0 | 1 | 0 | 0 | 0 | 0 | 0 | 1 | 1 | 0 | 1 | 0 | 0 | 1 | 0 | - | 0 | 1 |
| *Alpinia brevilabris* | 0 | 2 | 2 | 0 | 3 | 0 | 0 | 0 | 0 | 0 | 1 | 1 | 0 | 0 | 1 | 1 | 0 | 1 | 1 | 0 | 1 | 0 | - | 0 | 0 |
| *Alpinia caerulea* | 0 | 2 | 1&3 | 0 | 3 | 0 | 1 | 0 | 0 | 1 | 1 | 1 | 0 | 0 | 1 | 1 | 0 | 1 | 1 | 1 | 1 | 0 | - | 0 | 0 |
| *Alpinia carolinensis* | 0 | 2 | 1 | 0 | 3 | 1 | 1 | 0 | 0 | 1 | 0 | 0 | 0 | 0 | 1 | 1 | 0 | 1 | 1 | 1 | 1 | 0 | - | 0 | 1 |
| *Alpinia conchigera* | 0 | 2 | 1 | 0 | 3 | 2 | 0 | 0 | 0 | 0 | 0 | 0 | 0 | 0 | 1 | 1 | 0 | 1 | 1 | 1 | 0 | 0 | - | 0 | 1 |
| *Alpinia fax* | 0 | 1 | 1 | 0 | 3 | 0 | 0 | 0 | 0 | 0 | 0 | 0 | 0 | 0 | 1 | 1 | 0 | 1 | 0 | 0 | 1 | 0 | - | 0 | 1 |
| *Alpinia galanga* | 0 | 2 | 1 | 0 | 3 | 2 | ? | 0 | 0 | 0 | 0 | 0 | 0 | 0 | 1 | 0 | 0 | 1 | 1 | 1 | 0 | 0 | - | 0 | 1 |
| *Alpinia gigantifolia* | 0 | 2 | 1&2 | 0 | 3 | 1 | 1 | 0 | 1 | 0 | 0 | 0 | 0 | 0 | 1 | 0 | 0 | 1 | 1 | 1 | 0 | 0 | - | 0 | 0 |
| *Alpinia haenkei* | 0 | 1 | 1 | 0 | 3 | 0 | 1 | 0 | 0 | 0 | 2 | 0 | 0 | 0 | 1 | 1 | 0 | 1 | 1 | 1 | 1 | 0 | - | 0 | 0 |
| *Alpinia japonica* | 0 | 1 | 1 | 0 | 3 | 0 | ? | 0 | 0 | 0 | 0 | 0 | 0 | 0 | 1 | 1 | 0 | 1 | 0 | 0 | 1 | 0 | - | 0 | 1 |
| *Alpinia javanica* | 0 | 2 | 3 | 0 | 3 | 1 | 0 | 0 | 1 | 0 | 0 | 0 | 0 | 0 | 1 | 1 | 0 | 1 | 0 | 0 | 0 | 0 | - | 0 | 0 |
| *Alpinia katsumadai* | 0 | 1 | 1 | 0 | 3 | 0 | 0 | 1 | 0 | 0 | 2 | 0 | 0 | 0 | 1 | 1 | 0 | 1 | 1 | 0 | 1 | 0 | - | 0 | 1 |
| *Alpinia luteocarpa* | 0 | 1 | 2 | 0 | 3 | 0 | 1 | 0 | 0 | 1 | 0 | 0 | 0 | 0 | 1 | 1 | 0 | 1 | 1 | 0 | 1 | 0 | - | 0 | 0 |
| *Alpinia malaccensis* | 0 | 1 | 2 | 0 | 3 | 0 | 1 | 1 | 0 | 0 | 2 | 0 | 0 | 0 | 1 | 1 | 0 | 1 | 1 | 0 | 1 | 0 | - | 0 | 1 |
| *Alpinia nigra* | 0 | 1 | 2 | 0 | 3 | 2 | 0 | 0 | 0 | 0 | 1 | 0 | 0 | 0 | 1 | 1 | 0 | 1 | 1 | 1 | 1 | 0 | - | 0 | 1 |
| *Alpinia oblongifolia* | 0 | 1 | 2 | 0 | 3 | 2 | 1 | 0 | 0 | 0 | 2 | 0 | 0 | 0 | 1 | 1 | 0 | 1 | 0 | 0 | 0 | 0 | - | 0 | 0 |
| *Alpinia oceanica* | 0 | 1 | 1 | 0 | 3 | 1 | 1 | 0 | 0 | 1 | 0 | 0 | 0 | 0 | 1 | 1 | 0 | 1 | 1 | 1 | 1 | 0 | - | 0 | 0 |
| *Alpinia purpurata* | 0 | 1 | 1&3 | 0 | 3 | 0 | 0 | 1 | 0 | 1 | 0 | 0 | 0 | 0 | 1 | 1 | 0 | 1 | 1 | 0 | 1 | 0 | - | 1 | 1 |
| *Alpinia rafflesiana* | 0 | 2 | 1&3 | 0 | 3 | 1 | 0 | 0 | 0 | 0 | 0 | 0 | 0 | 0 | 1 | 1 | 0 | 1 | 0 | 0 | 1 | 0 | - | 0 | 0 |
| *Alpinia stachyodes* | 0 | 2 | 1 | 0 | 3 | 2 | 0 | 0 | 0 | 0 | 1 | 1 | 0 | 0 | 1 | 1 | 0 | 1 | 1 | 1 | 0 | 0 | - | 0 | 1 |
| *Alpinia zerumbet* | 0 | 0 | 2 | 0 | 3 | 1 | 1 | 0 | 0 | 0 | 2 | 0 | 0 | 0 | 1 | 1 | 0 | 1 | 1 | 1 | 0 | 0 | - | 0 | 0 |
| *Amomum koenigii* | 0 | 2 | 1 | 0 | 3 | 0 | 0 | 0 | 1 | 0 | 0 | 0 | 0 | 0 | 1 | 1 | 0 | 1 | 0 | 0 | 1 | 0 | - | 0 | 1 |
| *Amomum lappaceum* | 0 | 1 | 2 | 0 | 3 | 0 | 1 | 1 | 0 | 0 | 0 | 0 | 0 | 0 | 1 | 1 | 0 | 1 | 0 | 0 | 1 | 0 | - | 0 | 1 |
| *Amomum ochreum* | 0 | 2 | 1 | 0 | 3 | 0 | 1 | 1 | 1 | 0 | 0 | 0 | 0 | 0 | 1 | 1 | 0 | 1 | 1 | 0 | 1 | 1 | 0 | 0 | 1 |
| *Amomum sericeum* | 0 | 2 | 1&2 | 0 | 3 | 1 | 0 | 0 | 1 | 1 | 1 | 0 | 0 | 0 | 1 | 0 | 0 | 1 | 0 | 0 | 0 | 0 | - | 0 | 1 |
| *Aulotandra trigonocarpa* | 0 | ? | ? | 1 | 2 | 0 | 0 | 1 | 1 | 1 | ? | ? | 0 | 1 | 1 | 0 | 0 | 0 | - | - | - | 1 | 1 | 0 | 0 |
| *Boesenbergia acuminata* | 0 | 0 | 1 | 0 | 3 | 0 | 0 | 1 | 1 | 1 | 0 | 0 | 0 | 2 | 1 | 0 | 0 | 1 | 0 | 0 | 1 | 1 | 1 | 0 | 1 |
| *Burbidgea stenantha* | 0 | 1 | 1 | 0 | 3 | 0 | 0 | 1 | 0 | 1 | 0 | 0 | 0 | 0 | 1 | 1 | 0 | 1 | 0 | 0 | 1 | 1 | 0 | 1 | 0 |
| *Camptandra ovata* | 0 | 1 | 2 | 1 | 3 | 0 | 0 | 1 | 1 | 1 | 0 | 0 | 0 | 1 | 1 | 0 | 0 | 1 | - | - | - | 1 | 1 | 0 | 0 |
| *Cautleya gracilis* | 0 | 2 | 2 | 0 | 3 | 3 | 1 | 0 | 0 | 0 | 0 | 0 | 0 | 1 | 1 | 0 | 0 | 0 | - | - | - | 1 | 1 | 0 | 0 |
| *Cautleya spicata* | 0 | 2 | 2 | 0 | 3 | 0 | 0 | 0 | 1 | 1 | 0 | 0 | 0 | 2 | 1 | 0 | 0 | 1 | 1 | 0 | 1 | 1 | 1 | 0 | 0 |
| *Curcuma montana* | 0 | 1 | 1 | 0 | 3 | 0 | 0 | 1 | 1 | 1 | 0 | 0 | 0 | 2 | 1 | 1 | 0 | 1 | 0 | 0 | 1 | 1 | 0 | 0 | 0 |
| *Curcuma pierreana* | 0 | 1 | 1&2 | 0 | 3 | 2 | 1 | 0 | 1 | 1 | 0 | 0 | 0 | 2 | 1 | 1 | 0 | 1 | 0 | 0 | 1 | 1 | 1 | 0 | 1 |
| *Distichochlamys citrea* | 0 | 2 | 1&2 | 0 | 3 | 0 | 0 | 1 | 1 | 1 | 0 | 0 | 0 | 2 | 0 | - | - | 1 | 0 | 0 | 0 | 1 | 1 | 0 | 0 |
| *Elettaria cardamomum* | 0 | 2 | 3 | 0 | 3 | 0 | 0 | 0 | 0 | 0 | 1 | 0 | 0 | 0 | 1 | 1 | 0 | 1 | 0 | 0 | 1 | 0 | - | 0 | 1 |
| *Elettariopsis unifolia* | 0 | 2 | 1 | 0 | 3 | 0 | 0 | 0 | 1 | 1 | 1 | 0 | 0 | 0 | 1 | 1 | 0 | 1 | 0 | 0 | 0 | 0 | - | 0 | 1 |
| *Etlingera elatior* | 0 | 2 | 1 | 0 | 3 | 0 | 0 | 0 | 1 | 1 | 1 | 1 | 0 | 0 | 1 | 0 | 0 | 1 | 0 | 0 | 0 | 0 | - | 0 | 0 |
| *Etlingera linguiformis* | 0 | 1 | 2 | 0 | 3 | 1 | 1 | 0 | 1 | 0 | 1 | 1 | 0 | 0 | 1 | 1 | 0 | 1 | 0 | 0 | 0 | 0 | - | 0 | 0 |
| *Etlingera yunnanensis* | 0 | 1 | 2 | 0 | 1 | 1 | 1 | 0 | 1 | 0 | 1 | 1 | 0 | 0 | 1 | 0 | 0 | 1 | 0 | 0 | 0 | 0 | - | 0 | 0 |
| *Gagnepainia harmandii* | 0 | 1 | 1&2 | 1 | 3 | 0 | 0 | 0 | 0 | 0 | 0 | 1 | 0 | 2 | ? | ? | ? | 1 | 1 | 0 | 0 | 1 | 1 | 0 | 0 |
| *Geocharis aurantiaca* | 0 | 1 | 1 | 0 | 3 | 3 | 1 | 0 | 0 | 1 | 1 | 1 | 0 | 0 | 1 | 1 | 0 | 1 | 0 | 0 | 1 | 0 | - | 0 | 1 |
| *Geostachys densiflora* | 0 | 1 | 2 | 0 | 2 | 1 | 1 | 0 | 0 | 1 | 1 | 0 | 0 | 0 | 1 | 1 | 0 | 1 | 0 | 0 | 1 | 0 | - | 0 | 1 |
| *Globba aurea* | 0 | 1 | 1 | 1 | 2 | 0 | 1 | 1 | 1 | 1 | 0 | 0 | 0 | 1 | 1 | 1 | 0 | 1 | 1 | 0 | 1 | 1 | 1 | 1 | 1 |
| *Globba maculata* | 0 | 1 | 1&3 | 1 | 1 | 1 | 1 | 0 | 1 | 1 | 0 | 0 | 0 | 2 | 1 | 1 | 0 | 1 | 0 | 0 | 1 | 1 | 0 | 1 | 1 |
| *Globba pendula* | 0 | 1 | 1 | 1 | 1 | 1 | 1 | 0 | 0 | 1 | 0 | 0 | 0 | ? | 1 | 0 | 0 | 1 | 1 | 0 | 0 | 1 | 1 | 1 | 1 |
| *Globba sessiliflora* | 0 | 1 | 1 | 1 | 3 | 1 | 1 | 0 | 0 | 1 | 0 | 0 | 0 | 1 | 1 | 1 | 0 | 1 | 1 | 0 | 0 | 1 | 1 | 1 | 0 |
| *Globba spathulata* | 0 | 1 | 1&2 | 1 | 2 | 1 | 0 | 1 | 1 | 1 | 0 | 0 | 0 | 1 | 1 | 0 | 0 | 0 | - | - | - | 1 | 1 | 0 | 1 |
| *Hedychium coronarium* | 0 | 1 | 1 | 0 | 2 | 1 | 0 | 0 | 1 | 1 | 0 | 0 | 0 | 1 | 0 | - | - | 1 | 1 | 1 | 0 | 1 | 1 | 0 | 0 |
| *Hedychium gardnerianum* | 0 | 1 | 2 | 0 | 2 | 1 | 1 | 0 | 1 | 0 | 0 | ? | 0 | 1 | 0 | - | - | 1 | 1 | 1 | 1 | 1 | 1 | 0 | 0 |
| *Hedychium hasseltii* | 0 | 1 | 2 | 0 | 2 | 1 | 0 | 1 | 1 | 0 | 0 | 0 | 0 | 1 | 0 | - | - | 1 | 1 | 0 | 1 | 1 | 1 | 0 | 1 |
| *Hedychium muluense* | 0 | 1 | 2 | 0 | 2 | 0 | 0 | 1 | 0 | 1 | 0 | 0 | 0 | 1 | 0 | - | - | 1 | 1 | 0 | 0 | 1 | 1 | 1 | 0 |
| *Hemiorchis* sp. | 0 | 1 | 1&2 | 1 | 2 | 1 | 1 | 0 | 1 | 0 | 0 | 1 | 0 | 2 | 0 | - | - | 1 | 1 | 0 | 0 | 1 | 1 | 0 | 0 |
| *Hornstedtia conica* | 0 | 1 | 1 | 0 | 2 | 0 | ? | 0 | 1 | 0 | 1 | 1 | 0 | 1 | 1 | 1 | 0 | 1 | 1 | 0 | 1 | 0 | - | 0 | 1 |
| *Hornstedtia leonurus* | 0 | 1 | 1 | 0 | 2 | 0 | 1 | 0 | 0 | 0 | 1 | 1 | 0 | 0 | 1 | 1 | 0 | 1 | 0 | 0 | 0 | 0 | - | 0 | 0 |
| *Hornstedtia scottiana* | 0 | 2 | 1&2 | 0 | 2 | 0 | 1 | 0 | 0 | 0 | 1 | 1 | 0 | 0 | 1 | 1 | 0 | 1 | 0 | 0 | 0 | 0 | - | 0 | 0 |
| *Kaempferia pulchra* | 0 | 1 | 1&2 | 0 | 1 | 1 | 0 | 0 | 1 | 0 | 0 | 1 | 0 | 2 | 1 | 1 | 0 | 1 | 0 | 0 | 0 | 1 | 1 | 1 | 1 |
| *Monolophus sikkimensis* | 0 | 1 | 2 | 1 | 2 | 0 | 0 | 1 | 1 | 1 | 0 | 0 | 0 | 1 | 1 | 1 | 0 | 0 | - | - | - | 1 | 1 | 0 | 0 |
| *Newmania* sp. | 0 | 2 | 3 | 0 | 2 | 0 | 0 | 1 | 1 | 1 | 0 | 0 | 0 | 2 | 1 | 0 | 0 | 1 | 0 | 0 | 1 | 1 | 1 | 0 | 1 |
| *Plagiostachys escritorii* | 0 | 1 | 1 | 0 | 2 | 1 | 1 | 0 | 0 | 1 | 0 | 0 | 0 | 0 | 1 | 1 | 0 | 1 | 1 | ? | ? | 0 | - | 0 | 1 |
| *Plagiostachys philippinensis* | 0 | 1 | 1 | 0 | 2 | 4 | 1 | 0 | 0 | 1 | 0 | 0 | 0 | 0 | 1 | 1 | 0 | 1 | 0 | 0 | 1 | 0 | - | 0 | 1 |
| *Pleuranthodium sp.* | 0 | 2 | 1 | 0 | 2 | 2 | 1 | 0 | 0 | 0 | 0 | 1 | 0 | 0 | 1 | 1 | 0 | 1 | 1 | 1 | 0 | 0 | - | 0 | 1 |
| *Renealmia lucida* | 0 | 2 | 1 | 0 | 2 | 0 | 0 | 0 | 0 | 1 | 0 | 0 | 0 | 2 | 1 | 1 | 0 | 1 | 1 | 1 | 0 | 1 | 1 | 0 | 1 |
| *Renealmia occidentailis* | 0 | 1 | 1 | 0 | 2 | 1 | 1 | 0 | 0 | 1 | 0 | 0 | 0 | 0 | 1 | 1 | 0 | 1 | 1 | 1 | 1 | 1 | 1 | 0 | 1 |
| *Riedelia corallina* | 0 | 1 | 1 | 0 | 2 | 0 | 1 | 0 | 1 | 1 | 0 | 0 | 0 | 1 | 1 | 1 | 0 | 1 | 1 | 1 | 1 | 0 | - | 0 | 0 |
| *Riedelia sp.* | 0 | 1 | 1 | 0 | 2 | 0 | 0 | 0 | 1 | 1 | 0 | 0 | 0 | 1 | 1 | 0 | 0 | 1 | 1 | 0 | 0 | 1 | 1 | 0 | 0 |
| *Roscoea alpina* | 0 | 2 | 1&2 | 0 | ? | 0 | 0 | 0 | 1 | 1 | 0 | 0 | 0 | 2 | 1 | 1 | 0 | 1 | 0 | 0 | 1 | 1 | 1 | 0 | 0 |
| *Siamanthus siliquosus* | 0 | 2 | 2 | 0 | 2 | 1 | 0 | 0 | 0 | 1 | 0 | 0 | 0 | 0 | 1 | 1 | 0 | 1 | 0 | 0 | 1 | 0 | - | 0 | 0 |
| *Siliquamomum tonkinense* | 0 | 1&2 | 1 | 0 | 2 | 0 | 0 | 1 | 0 | 1 | 1 | 0 | 0 | 0 | 1 | 0 | 0 | 1 | 1 | 0 | 1 | 1 | 1 | 1 | 0 |
| *Siphonochilus aethiopicus* | 0 | 1 | 1 | 0 | 2 | 1 | 0 | 0 | 1 | 1 | 1 | 0 | 0 | 1 | 1 | 0 | 0 | 0 | - | - | - | 1 | 1 | 0 | 0 |
| *Siphonochilus kirkii* | 0 | 1 | 1 | 0 | 2 | 1 | 0 | 0 | 1 | 0 | 1 | 0 | 0 | 1 | 1 | 0 | 0 | 0 | - | - | - | 1 | 1 | 0 | 0 |
| *Vanoverberghia sepulchrei* | 0 | 1 | 1 | 0 | 2 | 0/1 | 1 | 0 | 0 | 1 | 0 | 0 | 0 | 0 | 1 | 1 | 0 | 1 | 1 | 0 | 1 | 0 | - | 0 | 1 |
| *Zingiber larsenii* | 0 | 2 | 1&3 | 0 | 2 | 0 | 1 | 1 | 1 | 0 | 0 | 0 | 0 | 1 | 1 | 1 | 0 | 1 | 1 | 0 | 1 | 1 | 1 | 0 | 0 |
| *Zingiber officinale* | 0 | 2 | 2 | 1 | 1 | 0 | 0 | 1 | 1 | 1 | 0 | 0 | 0 | 1 | 1 | 1 | 0 | 1 | 1 | 0 | 1 | 1 | 1 | 0 | 0 |
| *Zingiber spectabile* | 0 | 2 | 1 | 0 | 1 | 0 | 0 | 0 | 1 | 1 | 0 | 0 | 0 | 1 | 1 | 1 | 0 | 1 | 1 | 1 | 1 | 1 | 1 | 0 | 0 |
| *Zingiber thorelii* | 0 | 2 | 1&2 | 0 | 1 | 0 | 0 | 1 | 0 | 1 | 0 | 0 | 0 | 1 | 1 | 1 | 0 | 1 | 1 | 0 | 0 | 1 | 1 | 1 | 0 |

| Character number | 26 | 27 | 28 | 29 | 30 | 31 | 32 | 33 | 34 | 35 | 36 | 37 | 38 | 39 | 40 | 41 | 42 | 43 | 44 | 45 | 46 | 47 | 48 | 49 | 50 | 51 |
| --- | --- | --- | --- | --- | --- | --- | --- | --- | --- | --- | --- | --- | --- | --- | --- | --- | --- | --- | --- | --- | --- | --- | --- | --- | --- | --- |
| CI | 1 | 0.13 | 0.33 | 0.5 | 0.08 | 1 | 0.07 | 0.03 | 0.25 | 0.33 | 0.09 | 0.17 | 0.13 | 0.5 | 0.33 | 0.5 | 0.04 | 1 | 0.33 | 0.2 | 0.04 | 0.17 | 0.25 | 0.17 | 1 | 0.17 |
| RI | 1 | 0.53 | 0.75 | 0 | 0.71 | 1 | 0.46 | 0.29 | 0.4 | 0.86 | 0.64 | 0.81 | 0.81 | 0.975 | 0.6 | 0.94 | 0.57 | 1 | 0.33 | 0.71 | 0.47 | 0.55 | 0 | 0.71 | 1 | 0.87 |
| HI | 0 | 0.87 | 0.67 | 0.5 | 0.92 | 0 | 0.93 | 0.97 | 0.75 | 0.67 | 0.91 | 0.83 | 0.87 | 0.5 | 0.67 | 0.5 | 0.96 | 0 | 0.67 | 0.8 | 0.96 | 0.83 | 0.75 | 0.83 | 0 | 0.83 |
| **Cannaceae** |  |  |  |  |  |  |  |  |  |  |  |  |  |  |  |  |  |  |  |  |  |  |  |  |  |  |
| *Canna flaccida* | 0 | 0 | 0 | 0 | 1 | 0 | 0 | 1 | 0 | 0 | 1 | 2 | 0 | 0 | 0 | ? | 0 | 0 | - | 0 | 2 | 0 | 0 | 1 | 2 | 0 |
| *Canna indica* | 0 | 0 | 0 | 0 | 1 | 0 | 0 | 0 | 0 | 0 | 1 | ? | 0 | 0 | 0 | 1 | 0 | 0 | - | 0 | 2 | 0 | 0 | 1 | 2 | 0 |
| *Canna iridiflora* | 0 | 0 | 0 | 0 | 1 | 0 | 0 | 1 | 0 | 0 | 1 | 2 | 0 | 0 | 0 | 1 | 0 | 0 | - | 0 | 1 | 0 | 0 | 1 | 2 | 0 |
| *Canna paniculata* | 0 | 0 | 0 | 0 | 1 | 0 | 0 | 0 | 0 | 0 | 1 | ? | 0 | 0 | 0 | 1 | 0 | 0 | - | 0 | 2 | 0 | 0 | 1 | 2 | 0 |
| *Canna tuerckheimii* | 0 | 0 | 0 | 0 | 1 | 0 | 0 | 1 | 0 | 0 | 1 | 2 | 0 | 0 | 0 | 1 | 0 | 0 | - | 0 | 0 | 0 | 0 | 1 | 2 | 0 |
| *Canna x generalis* | 0 | 0 | 0 | 0 | 1 | 0 | 0 | 0 | 0 | 0 | 1 | ? | 0 | 0 | 0 | 1 | 0 | 0 | - | 0 | 0 | 0 | 0 | 1 | 2 | 0 |
| **Costaceae** |  |  |  |  |  |  |  |  |  |  |  |  |  |  |  |  |  |  |  |  |  |  |  |  |  |  |
| *Chamaecostus congestiflorus* | 0 | 4 | 0 | 0 | 0 | 0 | 2 | 0 | 0 | 1 | 1 | 3 | 1 | 2 | 0 | 0 | 0 | 0 | - | 0 | 0 | 0 | 0 | 1 | 1 | 1 |
| *Chamaecostus cuspidatus* | 0 | 4 | 0 | 0 | 0 | 0 | 1 | 0 | 0 | 1 | 1 | 3 | 1 | 2 | 0 | 0 | 0 | 0 | - | 0 | 0 | 0 | 0 | 1 | 1 | 0 |
| *Cheilocostus tonkinensis* | 0 | 4 | 0 | 0 | 0 | 0 | 2 | 1 | 0 | 1 | 0 | 3 | 1 | 2 | 0 | 0 | 0 | 0 | - | 0 | 0 | 0 | 0 | 1 | 1 | 1 |
| *Costus dubius* | 0 | 4 | 0 | 0 | 0 | 0 | 2 | 1 | 0 | 1 | 0 | 3 | 1 | 2 | 0 | 0 | 0 | 0 | - | 0 | 0 | 0 | 1 | 1 | 1 | 1 |
| *Costus guanaiensis* | 0 | 4 | 0 | 0 | 0 | 0 | 2 | 1 | 0 | 1 | 0 | 3 | 1 | 2 | 0 | 0 | 0 | 0 | - | 0 | 0 | 0 | 0 | 1 | 1 | 1 |
| *Costus hirsutus* | 0 | 4 | 0 | 0 | 0 | 0 | 2 | 1 | 0 | 1 | 0 | 3 | 1 | 2 | 0 | 0 | 0 | 0 | - | 0 | 0 | 0 | 0 | 1 | 1 | 1 |
| *Costus lucanusianus* | 0 | 4 | 0 | 0 | 0 | 0 | 2 | 1 | 0 | 1 | 0 | 3 | 1 | 2 | 0 | 0 | 0 | 0 | - | 0 | 0 | 0 | ? | 1 | 1 | 1 |
| *Costus megalobractea* | 0 | 4 | 0 | 0 | 0 | 0 | 2 | 1 | 0 | 1 | 0 | 3 | 1 | 2 | 0 | 0 | 0 | 0 | - | 0 | 0 | 0 | 0 | 1 | 1 | 1 |
| *Costus pulverulentus* | 0 | 4 | 0 | 0 | 0 | 0 | 2 | 0 | 0 | 1 | 1 | 3 | 1 | 2 | 0 | 0 | 0 | 0 | - | 0 | 0 | 0 | 0 | 1 | 1 | 0 |
| *Costus spiralis* | 0 | 4 | 0 | 0 | 0 | 0 | 2 | 1 | 0 | 1 | 0 | 3 | 1 | 2 | 0 | 0 | 0 | 0 | - | 0 | 0 | 0 | 1 | 1 | 1 | 1 |
| *Dimerocostus argenteus* | 0 | 4 | 0 | 0 | 0 | 0 | 1 | 0 | 0 | 1 | 0 | 3 | 1 | 2 | 0 | 0 | 0 | 0 | - | 0 | 0 | 0 | 0 | 1 | 1 | 1 |
| *Dimerocostus strobilaceus* | 0 | 4 | 0 | 0 | 0 | 0 | 1 | 0 | 0 | 1 | 1 | 3 | 1 | 2 | 0 | 0 | 0 | 0 | - | 0 | 0 | 0 | 0 | 1 | 1 | 1 |
| *Hellenia speciosa* | 0 | 4 | 0 | 0 | 0 | 0 | 2 | 1 | 0 | 1 | 0 | 3 | 1 | 2 | 0 | 0 | 0 | 0 | - | 0 | 0 | 0 | 0 | 1 | 1 | 1 |
| *Monocostus uniflorus* | 0 | 4 | 0 | 0 | 0 | 0 | 0&1 | 1 | 0 | 1 | 0 | 3 | 1 | 2 | 0 | 0 | 1 | 0 | - | 0 | 0 | 0 | 0 | 1 | 1 | 1 |
| *Tapeinochilus* sp. | 0 | 4 | 0 | 0 | 0 | 0 | 1 | 1 | 0 | 1 | 0 | 3 | 1 | 2 | 0 | 0 | 1 | 0 | - | 0 | 0 | 0 | 0 | 1 | 1 | 1 |
| **Heliconiaceae** |  |  |  |  |  |  |  |  |  |  |  |  |  |  |  |  |  |  |  |  |  |  |  |  |  |  |
| *Heliconia angusta* | 0 | 0 | 0 | 0 | 2 | 2 | 2 | 0 | 0 | 0 | 0 | 0 | 0 | 0 | 0 | 0 | 0 | 0 | - | 0 | 0 | 0 | 0 | 1 | 2 | 0 |
| *Heliconia bihai* | 0 | 0 | 0 | 0 | 2 | 2 | 2 | 0 | 0 | 0 | 0 | 0 | 0 | 0 | 0 | 0 | 0 | 0 | - | 0 | 2 | 0 | 0 | 1 | 2 | 0 |
| *Heliconia bourgaeana* | 0 | 0 | 0 | 0 | 2 | 2 | 2 | 0 | 0 | 0 | 0 | 0 | 0 | 0 | 0 | 0 | 0 | 0 | - | 0 | 2 | 0 | 0 | 1 | 2 | 0 |
| *Heliconia brenneri* | 0 | 0 | 0 | 0 | 2 | 2 | 2 | 0 | 0 | 0 | 0 | 0 | 0 | 0 | 0 | 0 | 0 | 0 | - | ? | ? | ? | ? | 1 | 2 | ? |
| *Heliconia chartacea* | 0 | 0 | 0 | 0 | 2 | 2 | 2 | 0 | 0 | 0 | 0 | 0 | 0 | 0 | 0 | 0 | 0 | 0 | - | 0 | 1 | 0 | 0 | 1 | 2 | 0 |
| *Heliconia griggsiana* | 0 | 0 | 0 | 0 | 2 | 2 | 2 | 0 | 0 | 0 | 0 | 0 | 0 | 0 | 0 | 0 | 0 | 0 | - | 0 | 2 | 0 | 0 | 1 | 2 | 0 |
| *Heliconia imbricata* | 0 | 0 | 0 | 0 | 2 | 2 | 2 | 0 | 0 | 0 | 0 | 0 | 0 | 0 | 0 | 0 | 0 | 0 | - | 0 | 2 | 0 | 0 | 1 | 2 | 0 |
| *Heliconia latispatha* | 0 | 0 | 0 | 0 | 2 | 2 | 2 | 0 | 0 | 0 | 0 | 0 | 0 | 0 | 0 | 0 | 0 | 0 | - | 0 | 2 | 0 | 0 | 1 | 2 | 0 |
| *Heliconia longiflora* | 0 | 0 | 0 | 0 | 2 | 2 | 2 | 0 | 0 | 0 | 0 | 0 | 0 | 0 | 0 | 0 | 0 | 0 | - | 0 | 2 | 0 | 0 | 1 | 2 | 0 |
| *Heliconia metallica* | 0 | 0 | 0 | 0 | 2 | 2 | 2 | 0 | 0 | 0 | 0 | 0 | 0 | 0 | 0 | 0 | 0 | 0 | - | 0 | 1 | 0 | 0 | 1 | 2 | 0 |
| *Heliconia papuana* | 0 | 0 | 0 | 0 | 2 | 2 | 2 | 0 | 0 | 0 | 0 | 0 | 0 | 0 | 0 | 0 | 0 | 0 | - | 0 | 1 | 0 | 0 | 1 | 2 | 0 |
| *Heliconia pendula* | 0 | 0 | 0 | 0 | 2 | 2 | 2 | 0 | 0 | 0 | 0 | 0 | 0 | 0 | 0 | 0 | 0 | 0 | - | 0 | 2 | 0 | 0 | 1 | 2 | 0 |
| *Heliconia solomonensis* | 0 | 0 | 0 | 0 | 2 | 2 | 2 | 0 | 0 | 0 | 0 | 0 | 0 | 0 | 0 | 0 | 0 | 0 | - | 0 | 2 | 0 | 0 | 1 | 2 | 0 |
| *Heliconia stricta* | 0 | 0 | 0 | 0 | 1 | 2 | 2 | 0 | 0 | 0 | 0 | 0 | 0 | 0 | 0 | 0 | 0 | 0 | - | 0 | 0 | 0 | 0 | 1 | 2 | 0 |
| *Heliconia subulata* | 0 | 0 | 0 | 0 | 2 | 2 | 2 | 0 | 0 | 0 | 0 | 0 | 0 | 0 | 0 | 0 | 0 | 0 | - | ? | ? | ? | ? | 1 | 2 | ? |
| *Heliconia vellerigera* | 0 | 0 | 0 | 0 | 2 | 2 | 2 | 0 | 0 | 0 | 0 | 0 | 0 | 0 | 0 | 0 | 0 | 0 | - | 0 | 1 | 0 | 0 | 1 | 2 | 0 |
| *Heliconia velutina* | 0 | 0 | 0 | 0 | 2 | 2 | 2 | 0 | 0 | 0 | 0 | 0 | 0 | 0 | 0 | 0 | 0 | 0 | - | 0 | 2 | 0 | 0 | 1 | 2 | 0 |
| **Lowiaceae** |  |  |  |  |  |  |  |  |  |  |  |  |  |  |  |  |  |  |  |  |  |  |  |  |  |  |
| *Orchidantha maxillarioides* | 0 | 0 | 0 | 0 | 1 | 0 | 1 | 1 | 0 | 0 | 2 | 3 | 0 | 0 | 1 | 0 | 0 | 0 | - | 0 | 2 | 0 | 0 | 0 | 2 | 0 |
| *Orchidantha sabahensis* | 0 | 0 | 0 | 0 | 1 | 0 | 1 | 1 | 0 | 0 | 2 | 3 | 0 | 0 | 1 | 0 | 0 | 0 | - | ? | ? | ? | ? | 0 | 2 | 0 |
| *Orchidantha stercorea* | 0 | 0 | 0 | 0 | 1 | 0 | 2 | 1 | 0 | 0 | 2 | 3 | 1 | 0 | 0 | 0 | 0 | 0 | - | ? | ? | ? | ? | 0 | 2 | 0 |
| *Orchidantha vietnamica* | 0 | 0 | 0 | 0 | 1 | 0 | 1 | 1 | 0 | 0 | 2 | 3 | 1 | 0 | 0 | 0 | 0 | 0 | - | ? | ? | ? | ? | 0 | 2 | ? |
| **Marantaceae** |  |  |  |  |  |  |  |  |  |  |  |  |  |  |  |  |  |  |  |  |  |  |  |  |  |  |
| *Calathea inocephala* | 0 | 0 | 0 | 0 | 0 | 0 | 2 | 1 | 0 | 0 | 0 | 3 | 0 | 0 | 0 | 0 | 0 | 1 | 0 | 0 | 2 | 0 | 0 | 1 | 2 | 0 |
| *Calathea macrosepala* | 0 | 0 | 0 | 0 | 0 | 0 | 2 | 1 | 0 | 0 | 0 | 3 | 0 | 0 | 0 | 0 | 0 | 1 | 0 | 0 | 2 | 0 | 0 | 1 | 2 | 0 |
| *Ctenanthe lanceolata* | 0 | 0 | 0 | 0 | 0 | 0 | 1 | 1 | 0 | 0 | 1 | 3 | 0 | 0 | 0 | 0 | 0 | 1 | 1 | 0 | 2 | 0 | 0 | 1 | 2 | 0 |
| *Donax canniformis* | 0 | 0 | 0 | 0 | 1 | 0 | 2 | 1 | 0 | 0 | 0 | 3 | 0 | 0 | 0 | 0 | 0 | 1 | 2 | 0 | 2 | 0 | 0 | 1 | 2 | 0 |
| *Haumania liebrechtsiana* | 0 | 0 | 0 | 0 | 2 | 0 | ? | ? | ? | ? | 1 | 3 | 0 | 0 | 0 | 0 | 0 | 1 | 2 | 0 | 2 | 0 | 0 | 1 | 2 | 0 |
| *Hylaeanthe unilateralis* | 0 | 0 | 0 | 0 | 0 | 0 | ? | ? | ? | 0 | ? | 2 | 0 | 0 | 0 | 0 | 0 | 1 | 0 | 0 | 2 | 0 | 0 | 1 | 2 | 0 |
| *Hypselodelphys scandens* | 0 | 0 | 0 | 0 | 1 | 0 | 2 | 0 | 0 | 0 | 1 | 3 | 0 | 0 | 0 | 0 | 0 | 1 | 0 | 0 | 2 | 0 | 0 | 1 | 2 | 0 |
| *Indianthus virgatus* | 0 | 0 | 0 | 0 | 1 | 0 | 2 | 1 | 0 | 0 | 0 | 3 | 0 | 0 | 0 | 0 | 0 | 1 | 1 | 0 | 2 | 0 | 0 | 1 | 2 | 0 |
| *Ischnosiphon inflatus* | 0 | 0 | 0 | 0 | 1 | 0 | 2 | 0 | 0 | 0 | 1 | 3 | 0 | 0 | 0 | 0 | 0 | 1 | 0 | 0 | 2 | 0 | 0 | 1 | 2 | 0 |
| *Maranta arundinacea* | 0 | 0 | 0 | 0 | 1 | 0 | 1 | 1 | 0 | 0 | 1 | 3 | 0 | 0 | 0 | 0 | 0 | 1 | 1 | 0 | 2 | 0 | 0 | 1 | 2 | 0 |
| *Marantochloa leucantha* | 0 | 0 | 0 | 0 | 1 | 0 | 2 | 1 | 0 | 0 | 1 | 3 | 0 | 0 | 0 | 0 | 0 | 1 | 0 | 0 | 2 | 0 | 0 | 1 | 2 | 0 |
| *Megaphrynium macrostachyum* | 0 | 0 | 0 | 0 | 1 | 0 | 2 | 0 | 0 | 0 | 1 | 3 | 0 | 0 | 0 | 0 | 0 | 1 | 0 | 0 | 2 | 0 | 0 | 1 | 2 | 0 |
| *Monotagma laxum* | 0 | 0 | 0 | 0 | 1 | 0 | 1 | 0 | 0 | 0 | 1 | 3 | 0 | 0 | 0 | 0 | 0 | 1 | 0 | 0 | 1 | 0 | 0 | 1 | 2 | 0 |
| *Phrynium* sp. | 0 | 0 | 0 | 0 | 1 | 0 | 1 | 1 | 0 | 0 | 0 | 3 | 0 | 0 | 0 | 0 | 0 | 1 | 0 | 0 | 2 | 0 | 0 | 1 | 2 | 0 |
| *Phrynium imbricatum* | 0 | 0 | 0 | 0 | 1 | 0 | 1 | 0 | 0 | 0 | 1 | 3 | 0 | 0 | 0 | 0 | 0 | 1 | 0 | 0 | 1 | 0 | 0 | 1 | 2 | 0 |
| *Phrynium interruptum* | 0 | 0 | 0 | 0 | 1 | 0 | 2 | 0 | 0 | 0 | 1 | 3 | 0 | 0 | 0 | 0 | 0 | 1 | 0 | 0 | 2 | 0 | 0 | 1 | 2 | 0 |
| *Pleiostachya pittieri* | 0 | 0 | 0 | 0 | 0 | 0 | ? | ? | 0 | 0 | 1 | 3 | 0 | 0 | 0 | 0 | 0 | 1 | 0 | 0 | 2 | 0 | 0 | 1 | 2 | 0 |
| *Sarcophrynium brachystachyum* | 0 | 0 | 0 | 0 | 1 | 0 | 1 | 1 | 0 | 0 | 1 | 3 | 0 | 0 | 0 | 0 | 0 | 1 | 2 | 0 | 2 | 0 | 0 | 1 | 2 | 0 |
| *Stachyphrynium sumatranum* | 0 | 0 | 0 | 0 | 0 | 0 | 2 | 0 | 0 | 0 | 1 | 2 | 0 | 0 | 0 | 0 | 0 | 1 | 0 | 0 | 2 | 0 | 0 | 1 | 2 | 0 |
| *Stromanthe stromanthoides* | 0 | 0 | 0 | 0 | 0 | 0 | 1 | 1 | 0 | 0 | 1 | 3 | 0 | 0 | 0 | 0 | 0 | 1 | 1 | 0 | 2 | 0 | 0 | 1 | 2 | 0 |
| *Thalia geniculata* | 0 | 0 | 0 | 0 | 1 | 0 | 2 | 0 | 0 | 0 | 1 | 3 | 0 | 0 | 0 | 0 | 0 | 1 | 2 | 0 | 2 | 0 | 0 | 1 | 2 | 0 |
| *Thaumatococcus daniellii* | 0 | 0 | 0 | 0 | 2 | 0 | 2 | 0 | 0 | 0 | 1 | 3 | 0 | 0 | 0 | 0 | 0 | 1 | ? | ? | ? | ? | ? | 1 | 2 | ? |
| *Trachyphrynium braunianum* | 0 | 0 | 0 | 0 | 0 | 0 | ? | ? | ? | ? | ? | 3 | 0 | 0 | 0 | 0 | 0 | 1 | 0 | 0 | 2 | 0 | 0 | 1 | 2 | 0 |
| **Musaceae** |  |  |  |  |  |  |  |  |  |  |  |  |  |  |  |  |  |  |  |  |  |  |  |  |  |  |
| *Musa acuminata* | 0 | 3 | 1 | 0 | 2 | 1 | 2 | 1 | 0 | 1 | 3 | 2 | 1 | 2 | 0 | 1 | 1 | 0 | - | 1 | 0 | 1 | 0 | 0 | 2 | 0 |
| *Musa acuminata subsp. malaccensis* | 0 | 3 | 1 | 0 | 1 | 1 | 2 | 1 | 0 | ? | ? | ? | 1 | ? | 0 | 1 | 1 | 0 | - | ? | ? | ? | ? | 0 | 2 | 0 |
| *Musa balbisiana* | 0 | 3 | ? | 0 | 2 | 1 | 2 | 0 | 0 | 0 | 2 | 2 | 1 | 2 | 0 | 1 | 1 | 0 | - | 1 | 0 | 1 | 0 | 0 | 2 | 0 |
| *Musa coccinea* | 0 | 3 | 1 | 0 | 1 | 1 | 2 | 1 | 0 | ? | 2 | 2 | 1 | 2 | 0 | 1 | 1 | 0 | - | 1 | 0 | 1 | 0 | 0 | 2 | 0 |
| *Musa mannii* | 0 | 3 | 1 | 0 | 2 | 1 | 2 | 0 | 0 | 0 | 2 | ? | 1 | 2 | 0 | 1 | 1 | 0 | - | 1 | 0 | 1 | 0 | 1 | 2 | 0 |
| *Musa peekelii* | 0 | 3 | 0 | 0 | 2 | 1 | 1 | 0 | 0 | ? | 2 | 2 | 1 | 2 | 0 | 1 | 1 | 0 | - | 1 | 0 | 1 | 0 | 0 | 2 | 0 |
| *Musa sakaiana* | 0 | 3 | 0 | 0 | 2 | 1 | 2 | 1 | 0 | 1 | 2 | 2 | 0 | 2 | 0 | 1 | 1 | 0 | - | 1 | 0 | 1 | 0 | 0 | 2 | 0 |
| *Musa schizocarpa* | 0 | 3 | 1 | 0 | 2 | 1 | 2 | 0 | 0 | 0 | 2 | 2 | 1 | 2 | 0 | 1 | 1 | 0 | - | 1 | 0 | 1 | 0 | 1 | 2 | 0 |
| *Musa sikkimensis* | 0 | 3 | 1 | 0 | 2 | 1 | 2 | 0 | 0 | 0 | 2 | 2 | 1 | 2 | 0 | 1 | 1 | 0 | - | 1 | 0 | 1 | 0 | 1 | 2 | 0 |
| *Musa textilis* | 0 | 3 | 1 | 0 | 2 | 1 | 2 | 1 | 0 | 1 | 2 | 2 | 1 | 2 | 0 | 1 | 1 | 0 | - | 1 | 0 | 1 | 0 | 0 | 2 | 0 |
| *Musa velutina* | 0 | 3 | 1 | 0 | 2 | 1 | 2 | 1 | 0 | ? | 2 | 2 | 1 | ? | 0 | 1 | 1 | 0 | - | 1 | 0 | 1 | 0 | 0 | 2 | 0 |
| *Musa × paradisiaca* | 0 | 3 | ? | 0 | 2 | 1 | 2 | 1 | 0 | 0 | 2 | ? | 1 | 2 | 0 | 1 | 1 | 0 | - | ? | ? | ? | ? | 0 | 2 | ? |
| *Ensete glaucum* | 0 | 3 | 1 | 0 | 2 | 1 | 2 | 1 | 0 | 0 | 2 | ? | 1 | 2 | 0 | 1 | 1 | 0 | - | 1 | 0 | 1 | 0 | 0 | 2 | 0 |
| *Ensete lasiocarpum* | 0 | 3 | 0 | 0 | 2 | 1 | 2 | 0 | 0 | 0 | 2 | ? | 1 | 2 | 0 | 1 | 1 | 0 | - | 1 | 0 | 1 | 0 | 0 | 2 | 0 |
| *Ensete perrieri* | 0 | 3 | 1 | 0 | 2 | 1 | 2 | 0 | 0 | 0 | 2 | ? | 1 | 2 | 0 | 1 | 1 | 0 | - | 1 | 0 | 1 | 0 | 0 | 2 | 0 |
| *Ensete superbum* | 0 | 3 | 1 | 0 | 2 | 1 | 1 | 0 | 0 | 0 | 1 | ? | 1 | 2 | 0 | 1 | 1 | 0 | - | ? | ? | ? | ? | ? | 2 | ? |
| *Ensete ventricosum* | 0 | 3 | 0 | 0 | 2 | 1 | 2 | 0 | 0 | 0 | 2 | ? | 1 | 2 | 0 | 1 | 1 | 0 | - | 1 | 0 | 1 | 0 | 0 | 2 | 0 |
| **Strelitziaceae** |  |  |  |  |  |  |  |  |  |  |  |  |  |  |  |  |  |  |  |  |  |  |  |  |  |  |
| *Phenakospermum guyannense* | 0 | 0 | 0 | 0 | 1 | 0 | 1 | 1 | 0 | 0 | 3 | 3 | 0 | 0 | 1 | 0 | 0 | 0 | - | 0 | 0 | 0 | 0 | 0 | 2 | 0 |
| *Ravenala madagascariensis* | 0 | 0 | 0 | 0 | 2 | 0 | 1 | 1 | 0 | 0 | 1 | 3 | 0 | 0 | 1 | 0 | 0 | 0 | - | 0 | 2 | 0 | 0 | 0 | 2 | 0 |
| *Strelitzia nicolai* | 0 | 0 | 0 | 0 | 1 | 0 | 2 | 0 | 0 | 0 | 1 | 3 | 0 | 0 | 1 | 0 | 0 | 0 | - | 0 | 0 | 0 | 0 | 1 | 2 | 0 |
| *Strelitzia reginae* | 0 | 0 | 0 | 0 | 1 | 0 | 1 | 0 | 0 | 0 | 1 | 3 | 0 | 0 | 1 | 0 | 0 | 0 | - | 0 | 0 | 0 | 0 | 0 | 2 | 0 |
| **Zingiberaceae** |  |  |  |  |  |  |  |  |  |  |  |  |  |  |  |  |  |  |  |  |  |  |  |  |  |  |
| *Aframomum angustifolium* | 0 | 0 | 0 | 0 | 1 | 0 | 0 | 1 | 0 | 0 | 3 | 2 | 1 | 2 | 0 | 0 | 1 | 0 | - | 0 | 2 | 0 | 0 | 1 | 1 | 2 |
| *Aframomum chrysanthum* | 0 | 1 | 0 | 0 | 1 | 0 | 0 | 1 | 0 | 0 | 3 | 2 | 2 | 2 | 0 | 0 | 1 | 0 | - | 0 | 0 | 0 | 0 | 1 | 1 | 2 |
| *Aframomum daniellii* | 0 | 0 | 0 | 0 | 1 | 0 | 0 | 0 | 0 | 0 | 3 | 2 | 1 | 2 | 0 | 0 | 0 | 0 | - | 0 | 0 | 1 | 0 | 1 | 1 | 2 |
| *Aframomum melegueta* | 0 | 0 | 0 | 0 | 1 | 0 | 0 | 0 | 0 | 0 | 3 | 2 | 1 | 2 | 0 | 0 | 0 | 0 | - | 0 | 0 | 1 | 0 | 1 | 1 | 2 |
| *Alpinia aquatica* | 0 | 0 | 0 | 0 | 0 | 0 | 2 | 0 | 0 | 0 | 2 | 2 | 2 | 2 | 0 | 0 | 0 | 0 | - | 0 | 1 | 0 | 0 | 1 | 1 | 2 |
| *Alpinia boia* | 0 | 2 | 0 | 0 | 1 | 0 | 0 | 1 | 0 | 0 | 2 | 3 | 2 | 2 | 0 | 0 | 1 | 0 | - | 0 | 1 | 0 | 1 | 1 | 1 | 2 |
| *Alpinia brevilabris* | 0 | 0 | 0 | 0 | 0 | 0 | 1 | 1 | 0 | 0 | 2 | 2 | 2 | 2 | 0 | 0 | 0 | 0 | - | 0 | 1 | 0 | 0 | 1 | 1 | 2 |
| *Alpinia caerulea* | 0 | 0 | 0 | 0 | 0 | 0 | 0 | 0 | 0 | 0 | 2 | 2 | 2 | 2 | 0 | 0 | 1 | 0 | - | 0 | 2 | 0 | 0 | 1 | 1 | 2 |
| *Alpinia carolinensis* | 0 | 1 | 0 | 0 | 1 | 0 | 0 | 0 | 0 | 0 | 2 | 2 | 2 | ? | 0 | 0 | 1 | 0 | - | 0 | 2 | 0 | 0 | 1 | 1 | 2 |
| *Alpinia conchigera* | 0 | 0 | 0 | 0 | 1 | 0 | 0 | 0 | 0 | 0 | 2 | 3 | 1 | 2 | 0 | 0 | ? | 0 | - | 1 | 2 | 1 | 0 | 1 | 1 | 2 |
| *Alpinia fax* | 0 | 1 | 0 | 0 | 1 | 0 | 1 | 0 | 0 | 0 | 3 | 3 | 2 | 2 | 0 | 0 | 1 | 0 | - | 0 | 2 | 0 | 0 | 1 | 1 | 2 |
| *Alpinia galanga* | 0 | 0 | 0 | 0 | 1 | 0 | 0 | 0 | 0 | 0 | 3 | 3 | 1 | 2 | 0 | 0 | 1 | 0 | - | 0 | 2 | 0 | 0 | 1 | 1 | 2 |
| *Alpinia gigantifolia* | 0 | 0 | 0 | 0 | 0 | 0 | 1 | 1 | 0 | 0 | 3 | ? | 1 | 2 | 0 | 0 | 1 | 0 | - | 0 | 2 | 0 | 0 | 1 | 1 | 2 |
| *Alpinia haenkei* | 1 | 1 | 0 | 0 | 1 | 0 | 2 | 1 | 0 | 0 | ? | 3 | 1 | 2 | 0 | 0 | 1 | 0 | - | 0 | 0 | 2 | 0 | 1 | 1 | 2 |
| *Alpinia japonica* | 0 | 1 | 0 | 0 | 1 | 0 | 2 | 1 | 0 | 0 | 2 | 3 | 2 | 2 | 0 | 0 | 1 | 0 | - | 0 | 0 | 2 | 0 | 1 | 1 | 2 |
| *Alpinia javanica* | 0 | 2 | 0 | 0 | 0 | 0 | 2 | 0 | 0 | 0 | ? | 2 | 1 | 2 | 0 | 0 | 0 | 0 | - | 1 | 2 | 0 | 0 | ? | 1 | ? |
| *Alpinia katsumadai* | 1 | 0 | 0 | 0 | 0 | 0 | 1 | 0 | 0 | 0 | 2 | 2 | 1 | 2 | 0 | 0 | 1 | 0 | - | 0 | 0 | 2 | 0 | 1 | 1 | 2 |
| *Alpinia luteocarpa* | 0 | 0 | 0 | 0 | 0 | 0 | 1 | 0 | 0 | 0 | 3 | 2 | 2 | 2 | 0 | 0 | 0 | 0 | - | 0 | 1 | 0 | 0 | 1 | 1 | 2 |
| *Alpinia malaccensis* | 1 | 2 | 0 | 0 | 1 | 0 | 0 | 0 | 0 | 0 | 2 | 3 | 1 | 2 | 0 | 0 | 1 | 0 | - | 0 | 0 | 2 | 0 | 1 | 1 | 2 |
| *Alpinia nigra* | 0 | 0 | 0 | 0 | 1 | 0 | 1 | 0 | 0 | 0 | 3 | 2 | 1 | 2 | 0 | 0 | 0 | 0 | - | 0 | 0 | 1 | 0 | 1 | 1 | 2 |
| *Alpinia oblongifolia* | 1 | 2 | 0 | 0 | 0 | 0 | 1 | 0 | 0 | 0 | 2 | ? | 1 | 2 | 0 | 0 | 1 | 0 | - | 0 | 0 | 1 | 0 | 1 | 1 | 2 |
| *Alpinia oceanica* | 0 | 0 | 0 | 0 | 0 | 0 | 1 | 0 | 0 | 0 | 3 | 2 | 2 | 2 | 0 | 0 | 0 | 0 | - | 0 | 1 | 0 | 0 | 1 | 1 | 2 |
| *Alpinia purpurata* | 0 | 0 | 0 | 0 | 0 | 0 | 1 | 0 | 0 | 0 | 3 | 2 | 2 | 2 | 0 | 0 | 0 | 0 | - | 0 | 1 | 0 | 0 | 1 | 1 | 2 |
| *Alpinia rafflesiana* | 0 | 2 | 0 | 0 | 1 | 0 | 2 | 0 | 0 | 0 | 1 | 3 | 2 | 2 | 0 | 0 | 1 | 0 | - | 0 | 0 | 2 | 0 | 1 | 1 | 2 |
| *Alpinia stachyodes* | 0 | 0 | 0 | 0 | 1 | 0 | 0 | 0 | 0 | 0 | 3 | 3 | 1 | 2 | 0 | 0 | 1 | 0 | - | 0 | 2 | 0 | 0 | 1 | 1 | 2 |
| *Alpinia zerumbet* | 1 | 2 | 0 | 0 | 1 | 0 | 2 | 1 | 0 | 0 | 2 | 2 | 1 | 2 | 0 | 0 | 1 | 0 | - | 0 | 0 | 2 | 0 | 1 | 1 | 2 |
| *Amomum koenigii* | 0 | 0 | 0 | 0 | 2 | 0 | 1 | 0 | 0 | 0 | 2 | 3 | 2 | 2 | 0 | 0 | 0 | 0 | - | 0 | 0 | 0 | 0 | 1 | 1 | 2 |
| *Amomum lappaceum* | 0 | 0 | 0 | 0 | 1 | 0 | 1 | 0 | 0 | 0 | 2 | 2 | 2 | 2 | 0 | 0 | 0 | 0 | - | 0 | 0 | 0 | 0 | 1 | 1 | 2 |
| *Amomum ochreum* | 0 | 1 | 0 | 0 | 2 | 0 | 0 | 0 | 0 | 0 | 2 | 2 | 2 | 2 | 0 | 0 | 1 | 0 | - | 0 | 0 | 0 | 0 | 1 | 1 | 2 |
| *Amomum sericeum* | 0 | 2 | 0 | 0 | 0 | 0 | 0 | 1 | 0 | 0 | 1 | 3 | 2 | 2 | 0 | 0 | 1 | 0 | - | 0 | 2 | 0 | 0 | 1 | 1 | 2 |
| *Aulotandra trigonocarpa* | 0 | 0 | 0 | ? | 0 | 0 | 0 | 0 | 0 | 0 | ? | 1 | 1 | 1 | 0 | 0 | ? | 0 | - | ? | ? | ? | ? | ? | 1 | ? |
| *Boesenbergia acuminata* | 0 | 2 | 0 | 0 | 0 | 0 | 1 | 0 | 1 | 0 | 1 | 1 | 1 | 1 | 0 | 0 | ? | 0 | - | 0 | 2 | 0 | 0 | 1 | 1 | 2 |
| *Burbidgea stenantha* | 0 | 0 | 0 | 1 | 0 | 0 | 0 | 0 | 0 | 0 | 2 | 2 | 2 | 2 | 0 | 0 | 1 | 0 | - | 0 | 0 | 0 | 0 | 1 | 1 | 1 |
| *Camptandra ovata* | 0 | 2 | 0 | 0 | 0 | 0 | 1 | 0 | 0 | 0 | 2 | 1 | 1 | 1 | 0 | 0 | 0 | 0 | - | 0 | 0 | 0 | 0 | 1 | 1 | 2 |
| *Cautleya gracilis* | 0 | 0 | 0 | 0 | 0 | 0 | 2 | 0 | 0 | 0 | 2 | 1 | 1 | 1 | 0 | 0 | 0 | 0 | - | 0 | 2 | 0 | 0 | 1 | 1 | 2 |
| *Cautleya spicata* | 0 | 2 | 0 | 0 | 0 | 0 | 1 | 0 | 0 | 0 | 2 | 1 | 1 | 1 | 0 | 0 | 1 | 0 | - | 0 | 2 | 0 | 0 | 1 | 1 | 2 |
| *Curcuma montana* | 0 | 1 | 0 | 0 | 0 | 0 | 0 | 1 | 0 | 0 | 2 | 1 | 1 | 1 | 0 | 0 | 0 | 0 | - | 0 | 2 | 0 | 0 | 1 | 1 | 2 |
| *Curcuma pierreana* | 0 | 0 | 0 | 0 | 0 | 0 | 2 | 0 | 0 | 0 | 2 | 1 | 1 | 1 | 0 | 0 | 1 | 0 | - | 0 | 1 | 0 | 0 | 1 | 1 | 2 |
| *Distichochlamys citrea* | 0 | 0 | 0 | 0 | 0 | 0 | 2 | 0 | 0 | 0 | ? | 1 | 1 | 1 | 0 | 0 | 0 | 0 | - | 0 | 2 | 0 | 0 | 1 | 1 | 1 |
| *Elettaria cardamomum* | 0 | 0 | 0 | 0 | 0 | 0 | 0 | 0 | 0 | 0 | 2 | ? | 2 | ? | 0 | 0 | 1 | 0 | - | 0 | 1 | 0 | 0 | 1 | 1 | 2 |
| *Elettariopsis unifolia* | 0 | 2 | 0 | 0 | 1 | 0 | 1 | 0 | 0 | 0 | 1 | 3 | 1 | 2 | 0 | 0 | 1 | 0 | - | 0 | 1 | 1 | 0 | 1 | 1 | 2 |
| *Etlingera elatior* | 0 | 0 | 0 | 0 | 0 | 0 | 2 | 1 | 0 | 0 | 0 | 3 | 1 | 2 | 0 | 0 | 0 | 0 | - | 0 | 1 | 0 | 0 | 1 | 1 | 2 |
| *Etlingera linguiformis* | 0 | 0 | 0 | 0 | 0 | 0 | 2 | 1 | 0 | 0 | 0 | 3 | 1 | 2 | 0 | 0 | 0 | 0 | - | 0 | 1 | 0 | 0 | 1 | 1 | 2 |
| *Etlingera yunnanensis* | 0 | 0 | 0 | 0 | 0 | 0 | 2 | 0 | 0 | 0 | 0 | 3 | 1 | 2 | 0 | 0 | 0 | 0 | - | 0 | 0 | 0 | 0 | 1 | 1 | 2 |
| *Gagnepainia harmandii* | 0 | 1 | 0 | 0 | 0 | 0 | 1 | 0 | 0 | 0 | 1 | 1 | 1 | 1 | 0 | 0 | 0 | 0 | - | 0 | 0 | 0 | 0 | 0 | 1 | 0 |
| *Geocharis aurantiaca* | 0 | 0 | 0 | 0 | 0 | 0 | 2 | ? | 0 | 0 | 2 | 3 | 2 | 2 | 0 | 0 | 1 | 0 | - | 0 | 2 | 0 | 0 | 1 | 1 | 2 |
| *Geostachys densiflora* | 0 | 2 | 0 | 0 | 1 | 0 | 0 | 1 | 0 | 0 | 3 | 3 | 2 | 2 | 0 | 0 | 1 | 0 | - | 0 | 1 | 0 | 0 | 1 | 1 | 2 |
| *Globba aurea* | 0 | 2 | 0 | 0 | 0 | 0 | 0 | 0 | 1 | 0 | 2 | 1 | 1 | 1 | 0 | 0 | 1 | 0 | - | 0 | 1 | 0 | 0 | 1 | 1 | 0 |
| *Globba maculata* | 0 | 2 | 0 | 0 | 0 | 0 | 0 | 0 | 1 | 0 | 2 | 1 | 2 | 1 | 0 | 0 | 1 | 0 | - | 0 | 0 | 0 | 0 | 1 | 1 | 2 |
| *Globba pendula* | 0 | 1 | 0 | 0 | 0 | 0 | 1 | 0 | 1 | 0 | 2 | 1 | 1 | 1 | 0 | 0 | 1 | 0 | - | 0 | 2 | 0 | 0 | 1 | 1 | 2 |
| *Globba sessiliflora* | 0 | 2 | 0 | 0 | 0 | 0 | 1 | 0 | 1 | 0 | 2 | 1 | 1 | 1 | 0 | 0 | 1 | 0 | - | 0 | 1 | 0 | 0 | 1 | 1 | 2 |
| *Globba spathulata* | 0 | 0 | 0 | 0 | 0 | 0 | 1 | 0 | 0 | 0 | 1 | 1 | 1 | 1 | 0 | 0 | 0 | 0 | - | 0 | 0 | 0 | 0 | 1 | 1 | 2 |
| *Hedychium coronarium* | 0 | 2 | 0 | 0 | 0 | 0 | 0 | 0 | 0 | 0 | 2 | 1 | 1 | 1 | 0 | 0 | 0 | 0 | - | 0 | 1 | 0 | 0 | 1 | 1 | 2 |
| *Hedychium gardnerianum* | 0 | 0 | 0 | 0 | 0 | 0 | 0 | 0 | 0 | 0 | 2 | 1 | 1 | 1 | 0 | 0 | ? | 0 | - | 0 | 1 | 0 | 0 | 1 | 1 | 2 |
| *Hedychium hasseltii* | 0 | 1 | 0 | 0 | 0 | 0 | 2 | 0 | 0 | 0 | 2 | 1 | 1 | 1 | 0 | 0 | 0 | 0 | - | 0 | 1 | 0 | 0 | 1 | 1 | 2 |
| *Hedychium muluense* | 0 | 2 | 0 | 0 | 0 | 0 | 1 | 0 | 0 | 0 | 2 | 1 | 1 | 1 | 0 | 0 | 1 | 0 | - | 0 | 2 | 0 | 0 | 1 | 1 | 1 |
| *Hemiorchis* sp. | 0 | 0 | 0 | 0 | 0 | 0 | 1 | 1 | 0 | 0 | 0 | 1 | 1 | 1 | 0 | 0 | 0 | 0 | - | 1 | 0 | 0 | 1 | 1 | 1 | 0 |
| *Hornstedtia conica* | 0 | 0 | 0 | 0 | 0 | 0 | 0 | 0 | 0 | 0 | 1 | 3 | 1 | 2 | 0 | 0 | 1 | 0 | - | 0 | 1 | 0 | 0 | 1 | 1 | 2 |
| *Hornstedtia leonurus* | 0 | 0 | 0 | 0 | 0 | 0 | 2 | 0 | 0 | 0 | 1 | 3 | 1 | 2 | 0 | 0 | 0 | 0 | - | 0 | 1 | 0 | 0 | 1 | 1 | 2 |
| *Hornstedtia scottiana* | 0 | 0 | 0 | 0 | 1 | 0 | 2 | 0 | 0 | 0 | 2 | 3 | 1 | 2 | 0 | 0 | 1 | 0 | - | 0 | 0 | 0 | 0 | 1 | 1 | 2 |
| *Kaempferia pulchra* | 0 | 0 | 0 | 0 | 0 | 0 | 1 | 0 | 0 | 0 | 1 | 1 | 1 | 1 | 0 | 0 | 1 | 0 | - | 0 | 0 | 1 | 0 | 1 | 1 | ? |
| *Monolophus sikkimensis* | 0 | 0 | 0 | 0 | 0 | 0 | 2 | 0 | 0 | 0 | 1 | 1 | 1 | 1 | 0 | 0 | 0 | 0 | - | 1 | 1 | 1 | 0 | 1 | 1 | 2 |
| *Newmania* sp. | 0 | 2 | 0 | 0 | 0 | 0 | 0 | 0 | 0 | 0 | 2 | 1 | ? | 1 | 0 | 0 | 1 | 0 | - | 0 | 0 | 1 | 0 | 1 | 1 | 1 |
| *Plagiostachys escritorii* | 0 | 2 | 0 | 0 | 0 | 0 | 0 | 1 | 0 | 0 | 2 | 2 | 2 | 2 | 0 | 0 | 1 | 0 | - | 0 | 0 | 0 | 0 | 1 | 1 | 2 |
| *Plagiostachys philippinensis* | 0 | 2 | 0 | 0 | 0 | 0 | 0 | 0 | 0 | 0 | ? | 2 | 2 | ? | 0 | 0 | 1 | 0 | - | 0 | 0 | 0 | 0 | 1 | 1 | ? |
| *Pleuranthodium sp.* | 0 | 0 | 0 | 0 | 0 | 0 | 0 | 0 | 0 | 0 | 2 | 2 | 1 | 2 | 0 | 0 | 1 | 0 | - | 0 | 0 | 0 | 0 | 1 | 1 | 2 |
| *Renealmia lucida* | 0 | 1 | 0 | 0 | 1 | 0 | 0 | 0 | 0 | 0 | 2 | 2 | 1 | 2 | 0 | 0 | 1 | 0 | - | 0 | 1 | 0 | 0 | 1 | 1 | 2 |
| *Renealmia occidentailis* | 0 | 2 | 0 | 0 | 0 | 0 | 2 | 1 | 0 | 0 | 1 | 2 | 1 | 2 | 0 | 0 | 1 | 0 | - | 0 | 2 | 0 | 0 | 1 | 1 | 2 |
| *Riedelia corallina* | 0 | 0 | 0 | 0 | 0 | 0 | 0 | 0 | 0 | 0 | 2 | 2 | 2 | 2 | 0 | 0 | 0 | 0 | - | 0 | 2 | 0 | 0 | 1 | 1 | 1 |
| *Riedelia sp.* | 0 | 0 | 0 | 0 | 0 | 0 | 0 | 0 | 0 | 0 | 1 | 2 | 2 | 2 | 0 | 0 | 0 | 0 | - | 0 | 1 | 0 | 0 | 1 | 1 | 1 |
| *Roscoea alpina* | 0 | 0 | 0 | 0 | 0 | 0 | 2 | 0 | 0 | 0 | 3 | 1 | 1 | 1 | 0 | 0 | 0 | 0 | - | 0 | 1 | 0 | 0 | 1 | 1 | 2 |
| *Siamanthus siliquosus* | 0 | 0 | 0 | 0 | 0 | 0 | 0 | 1 | 0 | 0 | 2 | 2 | 1 | 2 | 0 | 0 | 0 | 0 | - | 0 | 2 | 0 | 0 | 1 | 1 | 2 |
| *Siliquamomum tonkinense* | 0 | 0 | 0 | 1 | 0 | 0 | 0 | 0 | 0 | 0 | 2 | 3 | 2 | 2 | 0 | 0 | 1 | 0 | - | 0 | 0 | 0 | 0 | 1 | 1 | 1 |
| *Siphonochilus aethiopicus* | 0 | 2 | 0 | 0 | 0 | 0 | 0 | 0 | 0 | 0 | 2 | 1 | 1 | 1 | 0 | 0 | 1 | 0 | - | 0 | 1 | 0 | 1 | 1 | 1 | 2 |
| *Siphonochilus kirkii* | 0 | 2 | 0 | 0 | 0 | 0 | 1 | 0 | 1 | 0 | 2 | 1 | 1 | 1 | 0 | 0 | 1 | 0 | - | ? | ? | ? | ? | 1 | 1 | 2 |
| *Vanoverberghia sepulchrei* | 0 | 0 | 0 | 0 | 0 | 0 | 0 | 0 | 0 | 0 | 2 | 2 | 2 | 2 | 0 | 0 | 1 | 0 | - | 0 | 2 | 0 | 0 | 1 | 1 | 2 |
| *Zingiber larsenii* | 0 | 1 | 0 | 0 | 0 | 0 | 0 | 0 | 0 | 0 | 2 | 1 | 1 | 1 | 0 | 0 | 1 | 0 | - | 0 | 2 | 0 | 0 | 1 | 1 | 2 |
| *Zingiber officinale* | 0 | 0 | 0 | 0 | 0 | 0 | 1 | 0 | 0 | 0 | 2 | 1 | 1 | 1 | 0 | 0 | 0 | 0 | - | 0 | 2 | 0 | 0 | 1 | 1 | 2 |
| *Zingiber spectabile* | 0 | 1 | 0 | 0 | 0 | 0 | 0 | 0 | 0 | 0 | 2 | 1 | 1 | 1 | 0 | 0 | 1 | 0 | - | 0 | 1 | 0 | 0 | 1 | 1 | 2 |
| *Zingiber thorelii* | 0 | 2 | 0 | 0 | 0 | 0 | 0 | 0 | 0 | 0 | 2 | 1 | 1 | 1 | 0 | 0 | 1 | 0 | - | 0 | 1 | 0 | 0 | 1 | 1 | 2 |

**Table S3** Environmental data for all extant Zingiberales taxa where occurrence data is included in GBIF (accessed August 31^st^, 2015) combined with WorldClim Global Climate variables (Hijmans *et al.,* 2005), and Terrestrial Ecoregions of the World (TEOW) biome categories (Olson *et al.,* 2001). The biome category “other” is a combination of desert and xeric shrublands, flooded grasslands and savannas, mangroves, mediterranean forests, woodlands, and scrubs, as well as montane grasslands and shrublands.

|  | **Cannaceae** | **Costaceae** | **Heliconiaceae** | | **Lowiaceae** | **Marantaceae** | | **Musaceae** | **Strelitziaceae** | **Zingiberaceae** | |
| --- | --- | --- | --- | --- | --- | --- | --- | --- | --- | --- | --- |
| Occurrences | 1490 | 7534 | 9652 | | 18 | 20256 | | 363 | 156 | 15840 | |
| **Biome (in %)** |  |  |  | |  |  | |  |  |  | |
| Tropical and Subtropical | 92 | 98 | 98 | | 100 | 97 | | 94 | 89 | 86 | |
| Temperate | 1 | 0 | 0 | | 0 | 0 | | 4 | 0 | 12 | |
| Other* | 7 | 2 | 2 | | 0 | 3 | | 2 | 11 | 2 | |
| **Altitude Data (in m)** |  |  |  | |  |  | |  |  |  | |
| Average Altitude | 690 | 441 | 522 | | 263 | 286 | | 519 | 324 | 640 | |
| Minimum Altitude | -3 | -1 | -1 | | 13 | -17 | | 5 | 5 | -17 | |
| Maximum Altitude | 4596 | 4739 | 4849 | | 1463 | 4600 | | 2483 | 1661 | 4624 | |
| Range in Altitude | 4599 | 4740 | 4850 | | 145 | 4617 | | 2478 | 1656 | 4641 | |
| Standard Deviation of Altitude | 763 | 497 | 581 | | 326 | 428 | | 561.8 | 364.35 | 663 | |
| **Precipitation Data (in mm)** |  |  |  |  | | |  |  |  | |  |
| Average Driest Month | 50 | 70 | 74 | | 92 | 69 | | 69 | 57 | 74 | |
| Minimum Driest Month | 0 | 0 | 0 | | 4 | 0 | | 0 | 1 | 0 | |
| Maximum Driest Month | 360 | 524 | 503 | | 189 | 523 | | 303 | 225 | 525 | |
| Range of Driest Month | 360 | 524 | 503 | | 185 | 523 | | 303 | 224 | 525 | |
| Standard Deviation of Driest Month | 44.3 | 71 | 63 | | 65 | 66.159 | | 72.99 | 48.47 | 72.18 | |
| Average Wettest Month | 276 | 379 | 371 | | 364 | 355 | | 381 | 287 | 349 | |
| Minimum Wettest Month | 14 | 63 | 57 | | 252 | 57 | | 105 | 52 | 63 | |
| Maximum Wettest Month | 805 | 1245 | 976 | | 678 | 1509 | | 1651 | 539 | 2483 | |
| Range of Wettest Month | 791 | 1183 | 919 | | 426 | 1452 | | 1546 | 487 | 2420 | |
| Standard Deviation of Wettest Month | 123.14 | 140 | 133 | | 115 | 135.31 | | 149.69 | 111.74 | 141.98 | |
| Average Annual Precipitation | 1747 | 2535 | 2540 | | 2456 | 2391 | | 2343 | 1940 | 2305 | |
| Minimum Annual Precipitation | 38 | 240 | 236 | | 1506 | 236 | | 561 | 477 | 264 | |
| Maximum Annual Precipitation | 6099 | 9240 | 8163 | | 3103 | 8792 | | 4980 | 3951 | 8575 | |
| Range of Annual Precipitation | 6061 | 9000 | 7927 | | 1597 | 8556 | | 4419 | 3474 | 8311 | |
| Standard Deviation of Annual Precipitation | 767.94 | 1024 | 937 | | 545 | 995.32 | | 967.31 | 813.61 | 937.91 | |
| **Temperature Data (in °C)** |  |  |  |  | | |  |  |  | |  |
| Average Warmest Month | 30.2 | 30.8 | 30.3 | | 30.8 | 31.1 | | 30.3 | 30.7 | 29.4 | |
| Minimum Warmest Month | 13.2 | 8.1 | 8.9 | | 25.8 | 6 | | 20.1 | 22.7 | 11.6 | |
| Maximum Warmest Month | 38 | 40.7 | 36.2 | | 32.8 | 40.7 | | 40.7 | 36 | 40.8 | |
| Range of Warmest Month | 24.8 | 32.6 | 27.3 | | 7 | 34.7 | | 20.6 | 13.3 | 29.2 | |
| Standard Deviation of Warmest Month | 3.71 | 2.8 | 3.1 | | 1.6 | 2.48 | | 2.98 | 3.12 | 3.68 | |
| Average Coldest Month | 13.6 | 17.7 | 17.4 | | 18.3 | 18 | | 14.3 | 15.8 | 14.4 | |
| Minimum Coldest Month | -8.9 | -6.1 | -12.4 | | 9.8 | -10 | | -2.4 | 1.4 | -14.7 | |
| Maximum Coldest Month | 23.2 | 24 | 23.4 | | 23.3 | 23.8 | | 23.3 | 22.7 | 24.1 | |
| Range of Coldest Month | 32.1 | 30.1 | 35.8 | | 13.5 | 33.8 | | 25.7 | 21.3 | 38.8 | |
| Standard Deviation of Coldest Month | 4.82 | 3.4 | 3.7 | | 5.5 | 3.3 | | 5.55 | 5.53 | 6.09 | |
| Average Mean Annual Temperature | 22 | 24.2 | 23.8 | | 24.8 | 24.4 | | 22.7 | 23.5 | 22.1 | |
| Minimum Mean Annual Temperature | 4.3 | 2.3 | 2.3 | | 19 | 0.7 | | 12.4 | 13.8 | 0.5 | |
| Maximum Mean Annual Temperature | 27.9 | 29.5 | 28.7 | | 27.1 | 28.6 | | 27.6 | 28.1 | 29.3 | |
| Range of Mean Annual Temperature | 23.6 | 27.2 | 26.4 | | 8.1 | 27.9 | | 15.2 | 14.3 | 28.8 | |
| Standard Deviation of Mean Annual Temperature | 3.65 | 2.6 | 3 | | 2.4 | 2.45 | | 3.39 | 3.7 | 4 | |

**Table S4** Environmental data for Zingiberales included in the current study based on GBIF occurrence data (accessed August 31^st^, 2015) combined with WorldClim Global Climate variables (Hijmans *et al.,* 2005), and Terrestrial Ecoregions of the World (TEOW) biome categories (Olson *et al.,* 2001). The biome category “other” is a combination of desert and xeric shrublands, flooded grasslands and savannas, mangroves, mediterranean forests, woodlands, and scrubs, as well as montane grasslands and shrublands.

|  | **Cannaceae** | **Costaceae** | **Heliconiaceae** | **Lowiaceae** | **Marantaceae** | **Musaceae** | **Strelitziaceae** | **Zingiberaceae** |
| --- | --- | --- | --- | --- | --- | --- | --- | --- |
| Occurrences | 935 | 2234 | 2134 | 3 | 3274 | 87 | 130 | 3806 |
| **Biome (in %)** |  |  |  |  |  |  |  |  |
| Tropical  and Subtropical | 95 | 97 | 98 | 100 | 96 | 94 | 94 | 64 |
| Temperate | 0 | 0 | 0 | 0 | 0 | 1 | 0 | 34 |
| Other* | 5 | 3 | 2 | 0 | 4 | 5 | 6 | 2 |
| **Altitude Data (in m)** |  |  |  |  |  |  |  |  |
| Average Altitude | 690 | 384 | 445 | 282 | 347 | 668 | 253 | 582 |
| Minimum Altitude | -3 | 0 | -1 | 278 | -17 | 7 | 5 | -17 |
| Maximum Altitude | 4596 | 4739 | 4596 | 291 | 4516 | 2370 | 1661 | 4624 |
| Range in Altitude | 4599 | 4739 | 4597 | 13 | 4533 | 2363 | 1656 | 4641 |
| Standard Deviation of Altitude | 763 | 460 | 522 | 8 | 426.7 | 636.9 | 294 | 679.05 |
| **Precipitation Data (in mm)** |  |  |  |  |  |  |  |  |
| Average Driest Month | 50 | 66 | 75 | 104 | 56 | 54 | 62 | 72 |
| Minimum Driest Month | 0 | 0 | 0 | 104 | 0 | 0 | 1 | 0 |
| Maximum Driest Month | 360 | 516 | 412 | 105 | 523 | 303 | 225 | 521 |
| Range of Driest Month | 360 | 516 | 412 | 1 | 523 | 303 | 224 | 521 |
| Standard Deviation of Driest Month | 44.3 | 63 | 63 | 1 | 69.35 | 63.82 | 50.46 | 64.49 |
| Average Wettest Month | 276 | 374 | 376 | 260 | 350 | 333 | 309 | 311 |
| Minimum Wettest Month | 14 | 63 | 57 | 257 | 86 | 105 | 69 | 69 |
| Maximum Wettest Month | 805 | 1246 | 902 | 261 | 1107 | 1651 | 539 | 1346 |
| Range of Wettest Month | 791 | 1183 | 845 | 4 | 1021 | 1546 | 470 | 1277 |
| Standard Deviation of Wettest Month | 123.14 | 142 | 135 | 2 | 133.63 | 205.07 | 102.13 | 129.74 |
| Average Annual Precipitation | 1747 | 2466 | 2569 | 2363 | 2202 | 2001 | 2107 | 2094 |
| Minimum Annual Precipitation | 38 | 240 | 236 | 2349 | 462 | 561 | 629 | 424 |
| Maximum Annual Precipitation | 6099 | 9240 | 7437 | 2370 | 8792 | 4980 | 3951 | 7419 |
| Range of Annual Precipitation | 6061 | 9000 | 7201 | 21 | 8330 | 4419 | 3322 | 6995 |
| Standard Deviation of Annual Precipitation | 767.94 | 962 | 897 | 12 | 1050.87 | 993.77 | 762.27 | 897.78 |
| **Temperature Data (in °C)** |  |  |  |  |  |  |  |  |
| Average Warmest Month | 30.2 | 30.9 | 30.7 | 30.2 | 31.9 | 29.8 | 31.4 | 28.5 |
| Minimum Warmest Month | 13.2 | 8.1 | 13.2 | 30.1 | 12.6 | 22.6 | 22.7 | 11.6 |
| Maximum Warmest Month | 38 | 40.7 | 36.1 | 30.2 | 40.7 | 37 | 36 | 40.7 |
| Range of Warmest Month | 24.8 | 32.6 | 22.9 | 1 | 28.1 | 14.4 | 13.3 | 29.1 |
| Standard Deviation of Warmest Month | 3.71 | 2.6 | 2.7 | 0.1 | 2.66 | 3.06 | 2.87 | 3.36 |
| Average Coldest Month | 13.6 | 17.9 | 18 | 21.4 | 17.9 | 13.6 | 17.3 | 11.5 |
| Minimum Coldest Month | -8.9 | -6.1 | -8.2 | 21.4 | -10 | 2.1 | 1.4 | -12.4 |
| Maximum Coldest Month | 23.2 | 24 | 23.4 | 21.4 | 23.8 | 23.3 | 22.7 | 24 |
| Range of Coldest Month | 32.1 | 30.1 | 31.6 | 0 | 33.8 | 21.2 | 21.3 | 36.4 |
| Standard Deviation of Coldest Month | 4.82 | 3.7 | 3.3 | 0 | 3.53 | 6.1 | 4.53 | 6.14 |
| Average Mean Annual Temperature | 22 | 24.3 | 24.2 | 25.6 | 24.8 | 22.2 | 24.4 | 20.5 |
| Minimum Mean Annual Temperature | 4.3 | 2.3 | 4.3 | 25.6 | 4.4 | 13.4 | 15.1 | 0.5 |
| Maximum Mean Annual Temperature | 27.9 | 29.5 | 28.4 | 25.6 | 28.6 | 27.5 | 28.1 | 28.2 |
| Range of Mean Annual Temperature | 23.6 | 27.2 | 24.1 | 0 | 24.2 | 14.1 | 13 | 27.7 |
| Standard Deviation of Mean Annual Temperature | 3.65 | 2.6 | 2.6 | 0 | 2.36 | 3.61 | 3.09 | 3.9 |

**Table S5** Key to Zingiberales seeds.

| 1. | Seeds in thickened endocarp. | Heliconiaceae |
| --- | --- | --- |
| 1’. | Seeds otherwise. | 2. |
| 2. | Seeds with stomata, usually dark brown or black in color. | Cannaceae |
| 2’. | Seeds otherwise. | 3. |
| 3. | Seeds with trichomes, but lack an external chalazal indentation and taper in the chalazal region. | Lowiaceae |
| 3’. | Seeds without trichomes, or if trichomes are present (e.g., *Globba*, Zingiberaceae) seeds taper in the chalazal region or have an external chalazal indentation. | 4. |
| 4. | Seeds with a perisperm canal. | Marantaceae |
| 4’. | Seeds without a perisperm canal. | 5. |
| 5. | Seeds dark brown or black in color, arillate, and lack an operculum (aril brightly colored blue, red, or orange). | Strelitziaceae |
| 5’. | Seeds otherwise, often with a distinctive operculum at micropyle. | 6. |
| 6. | Seeds with a thickened, weakly recurved micropylar collar, a short bulbous embryo, and a *Musa*-type chalazal chamber. | Musaceae |
| 6’. | Seeds otherwise, and if a chalazal chamber is present, it is not of the *Musa*-type. | 7. |
| 7. | Seeds with a strongly recurved micropylar collar, elongate straight embryos, and a *Costus*-type chalazal chamber. | Costaceae |
| 7’. | Seeds otherwise, either lacking a chalazal chamber, or if chalazal chamber is present, it is never of the *Costus*-type. | Zingiberaceae |
